# Chemoselective Generation of the 1,3-Benzdiyne Synthon

**DOI:** 10.1021/acs.joc.6c00616

**Published:** 2026-09-07

**Authors:** Fredrik Barnå, Manoj D. Patil, Fredric J. L. Ingner, Andreas Orthaber, Paul J. Gates, Lukasz T. Pilarski

**Affiliations:** † Department of Chemistry for Life Sciences, 8097 Uppsala University, Box 576, 75123 Uppsala, Sweden; ‡ Department of Chemistry − Ångström Laboratory, Uppsala University, Box 523, 75120 Uppsala, Sweden; § School of Chemistry, 1980 University of Bristol, Cantock’s Close, Clifton, Bristol BS8 1TS, United Kingdom

## Abstract

A versatile 1,3-benzdiyne precursor is reported that enables chemoselective, sequential generation of aryne triple bonds on the same aromatic ring under mild conditions. This is achieved from a single aromatic ring bearing an iodonium unit—enabling access to one aryne triple bond via deprotonation—and a “Kobayashi”-type *ortho*-silylaryl triflate unit, from which the second aryne triple bond can be released using fluoride. Each triple bond undergoes regioselective trapping. DFT studies reveal high-to-very high levels of ground state distortion in each aryne system. The approach leverages the unique reactivity of arynes to construct the framework of prized fluoride-activated precursors used in the next step. This strategy grants modular access to otherwise difficult-to-generate 1,2,3,4-substitution motifs.

## Graphical abstract

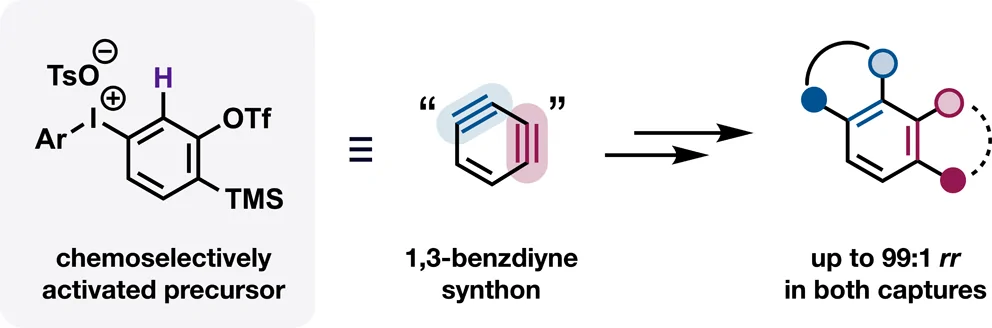

## Introduction

The trapping of aryne triple bonds is a powerful tactic for decorating vicinal carbons on (hetero)­arenes. It allows the regioselective introduction of triel-, adamantogen-, pnictogen-, chalcogen- and halogen-based groups in a remarkable range of combinations. This versatility has greatly benefited from the emergence of convenient precursors that can be activated using fluoride^1^ or mild bases.^2,3^ Arynes have served as key intermediates in the syntheses of bioactive compound libraries,^4^ natural products,^5^ polycyclic systems,^6^ biaryls,^7^ polymers,^8^ organometallic complexes^9^ and other valuable frameworks.^10^ Unfortunately, preparing elaborated aryne precursors that allow such versatility can be laborious; many otherwise impressive new aryne transformations are still often showcased on a handful of simple frameworks.

To advance aryne chemistry beyond conventional vicinal difunctionalization, several approaches to access benzdiyne and benztriyne synthons (Figure 1A), have been pursued.^11^ All involve the sequential generation of triple bonds on the same ring, either in one-pot procedures or as separate steps. Routes to the 1,3-benzdiyne (Figure 1B) synthon have been notably sparse and have been mostly reliant on harsh bases (*e.g*., NaNH_2_ for precursor **1a** ^12^ or BuLi for **1b** ^13^) or additional steps to prepare each precursor component prior to aryne release.^14^ Precursor **1c** is activated under mild conditions using fluoride to give **2a**.^15^ However, regioselectivity requires the differentiated kinetics of fluoride activation offered by different silanes (by contrast, **1d** gives **2b**).

**Figure 1:**
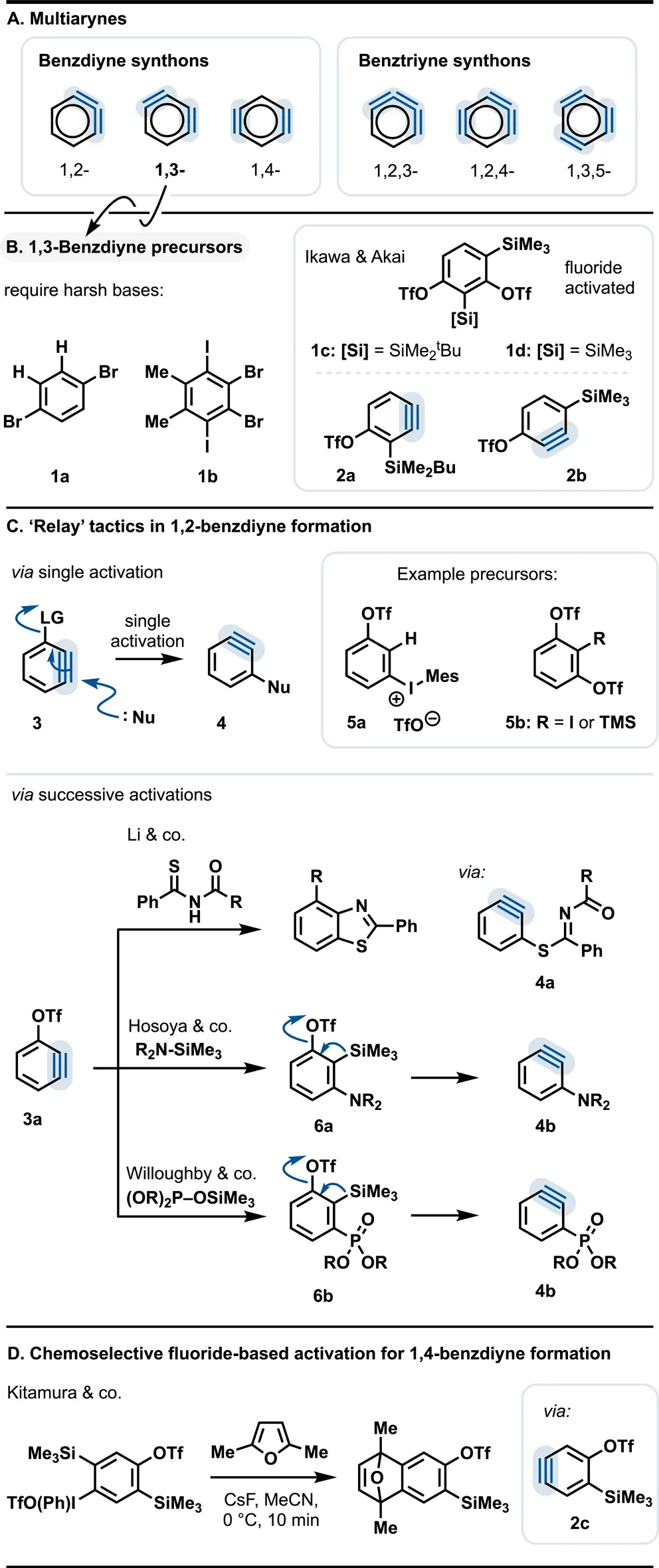
Multiarynes, approaches to 1,3-benzdiynes and benzdiynes from chemoselective/sequential activation.

Aryne precursors featuring two nucleofuges have offered a notable advances in versatility. The ‘relay’ approach to 1,2-benzdiynes (via **3** to **4**, Figure 1C and D) marks a particularly elegant design.^16^ For example, precursors **5a** and **5b** can give **3**, nucleophilic attack upon which leads to the elimination of the C3 leaving group to reveal the second triple bond (**4**). Li and co-workers used this approach to generate substituted benzothiazoles via **4a**.^16d^ Hosoya and Willoughby have shown that tethering a silane to the nucleophile (attack on **3a**) can give **6**, which are themselves fluoride-activated aryne precursors.^16a–16c^ These mark valuable examples of aryne reactivity being used to directly construct the framework of a fluoride-activated precursor. Kitamura and co-workers demonstrated that chemoselective activation by fluoride is possible for both 1,3- and 1,4-benzdiyne-based building blocks in which the elimination of a phenyliodonio group is preferred over that of triflate (Figure 1D). This allows the elaboration of the Kobayashi precursor backbone via cycloaddition reactions (e.g., of **2c**).^16e^

## Results and Discussion

We envisaged a 1,3-benzdiyne precursor, **8**, from which two triple bonds might be generated chemoselectively via either 1) regioselective deprotonation at the interstitial C–H *ortho* to the iodonium nucleofuge^16g,17^ to give **9a** or, 2) F^–^-induced activation of the *ortho-*silyl triflate moiety in **5** to give **9b**. We obtained **8** from boryl Kobayashi-type aryne precursor **7** (61% over 4 steps), which our group and that of Hosoya have previously shown to be a versatile building block for elaborating the precursor backbone.^18^ The tosylate counterion granted easy precipitation of **8**, and a convenient ^1^H NMR handle.

Preliminary DFT calculations indicated a remarkable distortion of the internal angles at C1 and C2 in **9a** (Δθ = θ_C1_ – θ_C2_ = 23.8°, Scheme 1), which is predictive of very high C1 regioselectivity in nucleophilic trapping reactions, as per Garg and Houk’s Aryne Distortion Model.^19^ By contrast, **9b** was predicted to have a C1-biased distortion of only 3.9°, which anticipates poor regioselectivity and underscores the dramatically reduced influence of remote regiocontrolling groups (*cf*. 3-OTf-benzyne vs 4-OTf-benzyne, Scheme 1—See Supporting information (SI) for details). Additionally, attempts to generate and trap **9b** from **8** using a variety of fluoride sources (CsF, Me_4_NF, and Bu_4_NF) invariably led to complex mixtures, including those complicated by the *ipso* substitution of the iodonium group. However, using conditions optimized via a Design of Experiments (DoE) approach^20^ (see SI for details), aryne **9a** could be generated and intercepted with a range of arynophiles **10** (Scheme 2). We prioritized difunctionalizing arynophiles to prevent elimination of triflate from **9a**, which would revert our system to an iteration of the relay methodology (*cf*. Figure 1C). We also sought to avoid mechanistic confusion from aryne intermediate trapping and *ipso* substitution of the iodonium group that arise from the use of simple nucleophiles.^21^ C1,C2-Difunctionalization of **9a** also affords 1,2,3,4-tetrasubstituted arenes, which can be challenging to access.^17c,22^

**Scheme 1:**
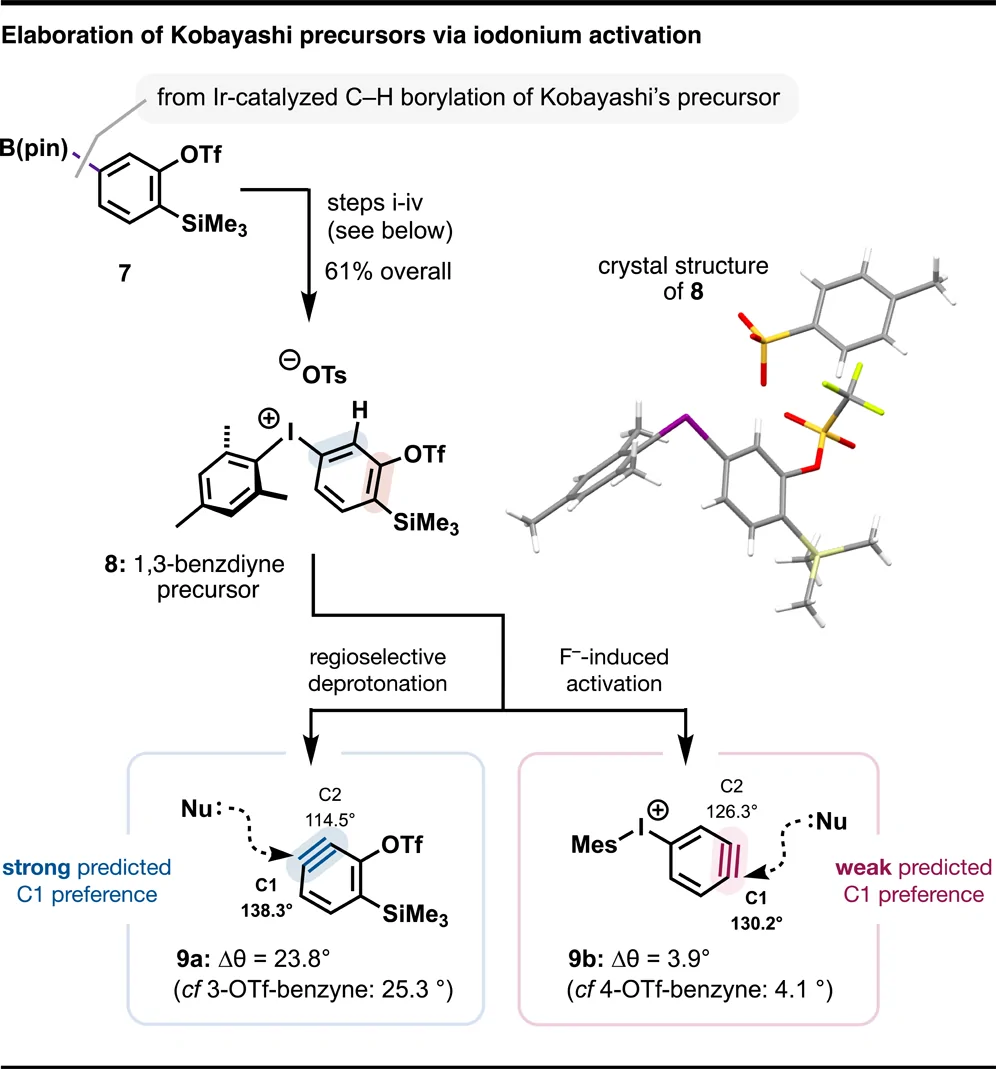
Synthetic Route to 1,3-Benzdiyne Precursor **8** and the Attendant Regioselectivity Rationale Based on the Aryne Distortion Model^a^

a Synthesis of **8**: (i) Diethanolamine (1.2 equiv), Et_2_O, 20 °C, 19 h, (ii) HCl_(aq)_ (1 M), Et_2_O, 20 °C, 10 min (iii) (Diacetoxy)­iodomesitylene (1.1 equiv), BF_3_·Et_2_O (3.0 equiv), CH_2_Cl_2_, 0–20 °C, 19 h, (iv) NaOTs_(aq)_ (2 M), CH_2_Cl_2_, 20 °C, 10 min. Calculations were performed at the PBEh-3c level of theory (see SI for details).

**Scheme 2:**
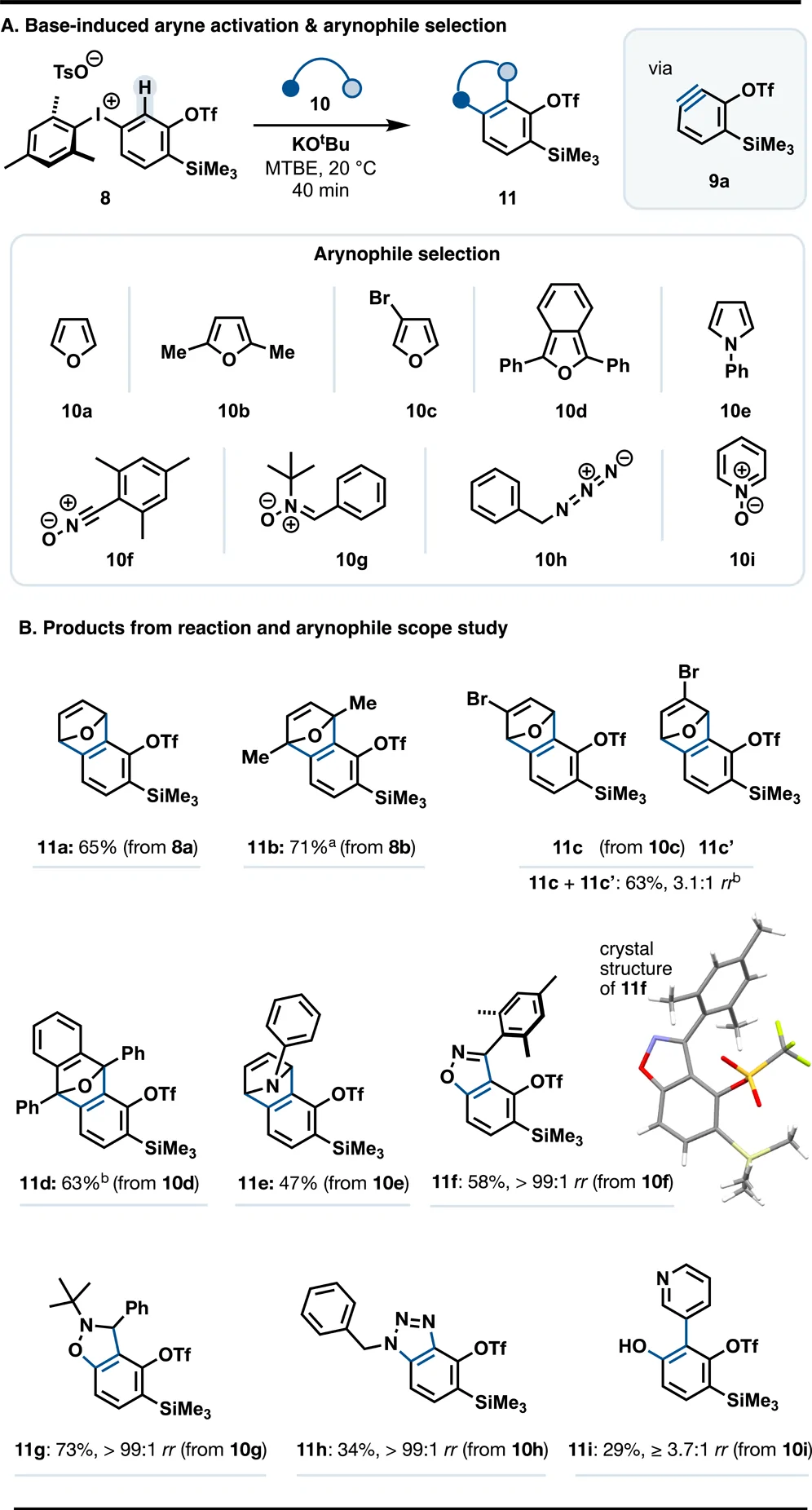
Base-Enabled Activation and Trapping of 1,3-Benzdiyne Precursor **8** ^b^ ^,^ ^c^ ^,^ ^a^

a 0.3 mmol scale **8**.
b 1.0 mmol scale **8**.
c General Conditions: **8** (0.2–1.0 mmol), 10 (2.0 equiv), KO^t^Bu (1.6 equiv), MTBE (0.04 M), 20 °C, 40 min. Isolated yields. Major/only detected regioisomer shown.

Initial reactions with **10a**–**e** gave the corresponding [4 + 2] cycloaddition products **11a**–**e** in moderate to good yields, generally confirming the viability of this approach. Unsymmetrical 3-bromofuran, **10c**, gave **11c** and **11c’** as a separable mixture of regioisomers (*rr* = 3:1). This marks a rare example of regioselective [4 + 2] cycloadditions with arynes. Product **11d** (obtained from isobenzofuran **10d** in 63% yield) is a congener of previously reported acene building blocks,^23^ and is equipped with the Kobayashi-type aryne precursor unit, which we envisage may broaden options for the bottom-up synthesis of new polycyclic aromatic hydrocarbons (PAHs).^6b–6d,24^ 1,3-Dipolar arynophiles **10f**-**h** gave the corresponding capture products **11f**-**h** in yields up to 73%, but with exclusive regioselectivity in favor of nucleophilic attack at C1, in line with the remarkably high distortion predicted by DFT calculations (Scheme 1). Product **11i**, incorporating a 3-substituted pyridine unit, was obtained from **10i** via rearrangement following the initial attack by oxygen.^25^ Notably, products **11a**-**i** represent a new cohort of 1,2,3,4-substituted arenes and novel Kobayashi-type precursors. This approach might thus serve to alleviate the paucity of structurally diverse fluoride-activated aryne precursors—a long-standing bottleneck for the broader adoption of arynes in synthesis.

To demonstrate the usefulness of precursors **11**, we studied capture reactions enabled by their fluoride-activated *ortho*-silylaryl triflate systems (Scheme 3). Computed Δθ values for novel arynes **12a**–**g** (Scheme 3A) followed expected general trends described previously for the proximity of electronegative atoms^19a,26^ and fused heterocyclic rings^19b,27^ The capture of **12c** with 3-phenylsydnone^28^ gave **13a** in 58% yield and 1.5:1 *rr* (Scheme 3B). A higher *rr* of 7:1 was obtained on trapping aryne **12c’** with 4-iodoaniline (product **13b**), in accordance with the greater proximity of the bromide to the triple bond. The adduct of **12d** and diazoacetic acid methyl ester^29^ gave the indazole **13c** in 55% yield as the only observed regioisomer. Trapping products **13d**–**f**, were obtained from arynes **12e**, **12f**, and **12g**, respectively, in moderate-to-good yields (50–75%) with regioselectivities tracking previously established trends for aromatic amine nucleophiles (**13e**, 11:1 *rr*), alkyl amines (**13d**, 6:1 *rr*) and thiophenols (**13f**, >2:1 *rr*).^30^ Among the 4-iodoaniline derivatives **13b**, **13e**, **13g**, and **13h**, regioselectivities increased as expected, according to calculated Δθ values for arynes **12c’**, **12e**, and **12g**, albeit with **12e** giving a slightly lower *rr*. Products **13a** and **13c** exemplify the new route to otherwise challenging 1,2,3,4-tetrasubsituted arene motifs this methodology grants.

**Scheme 3:**
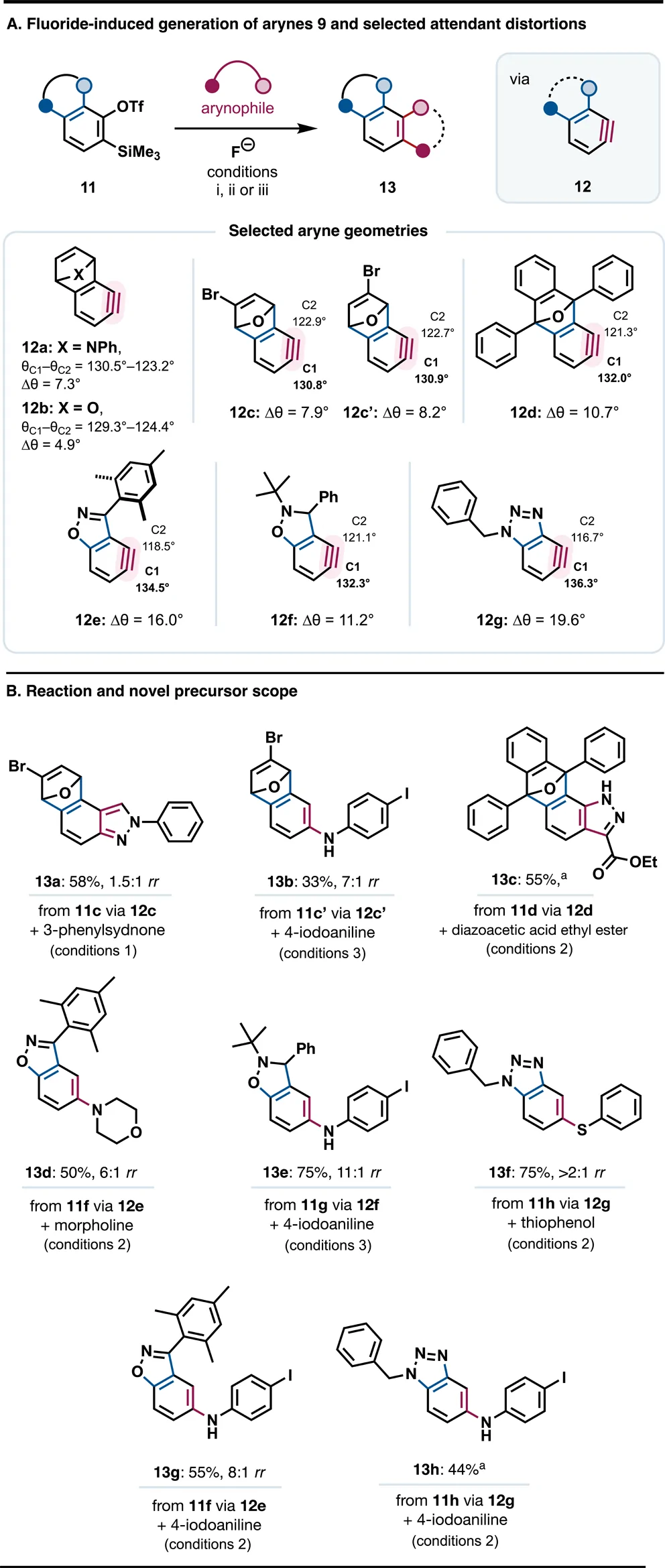
a Only observed regioisomer. Calculations were performed at the PBEh-3c level of theory (see SI for details).
b Isolated yields. Major/only detected regioisomer shown. *Conditions* 1: Arynophile (1.5 equiv), NH_4_F·H_2_O (1.3 equiv), THF, 20 °C, 18 h. *Conditions* 2: Arynophile (1.5 equiv), KF (3.0 equiv), 18-crown-6 (3.0 equiv), 20 °C, 18 h. *Conditions 3*: Arynophile (1.5 equiv), CsF (5.0 equiv), 18-crown-6 (3.0 equiv), MeCN, 20 °C, 18 h.

## Conclusion

In summary, we have shown the design and activation of a novel 1,3-benzdiyne precursor, **8**, which may be obtained conveniently from the commercially available Kobayashi benzyne precursor. Uniquely, its two aryne precursor units may be activated chemoselectively under mild conditions, allowing the use of diverse aryne trapping reactions for the elaboration of the Kobayashi precursor backbone (compounds **11a** to **11i**). The subsequent activation by fluoride gave a new library of previously unavailable arynes (**12**) and their derivatives, including those containing 1,2,3,4-substituted motifs. We anticipate the greater use of chemoselective benzdiyne generation in the synthesis of bioactive compound libraries and for the exploration of conventionally difficult-to-access chemical space. Studies on refining regiocontrol in these and closely related systems are ongoing in our laboratory.

## Supplementary Material



## Data Availability

The data underlying this study are available in the published article and its Supporting Information.

## Notes

General information, detailed experimental and computational procedures, characterization data, NMR spectra, and crystallographic data (PDF)
