# Supplementary material for: Chemoselective Generation of the 1,3-Benzdiyne Synthon
Source: J Org Chem. 2026 Sep 7;91(37):12490–5. doi: 10.1021/acs.joc.6c00616 (PMC13595903; doi:10.1021/acs.joc.6c00616)
Supplement: Supplementary file 1 [file jo6c00616_si_001.pdf]

Chemoselective Generation of the 1,3-Benzdiyne Synthon

Supporting Information

Fredrik Barnå,[a] Manoj D. Patil,[a] Fredric J. L. Ingner,[a] Andreas Orthaber,[b] Paul J. Gates[c] and Lukasz T. Pilarski*[a]

a. Department of Chemistry for Life Science, Uppsala University, Box 576, 75123, Uppsala, Sweden. Website: https://www.pilarski.group

b. Department of Chemistry – Ångström Laboratory, Uppsala University, Box 523, 75120 Uppsala, Sweden.

c. School of Chemistry, University of Bristol, Cantock's Close, Clifton, Bristol, BS8 1TS, United Kingdom.

Contents

General experimental information	S3
Synthesis of starting materials	S5
Synthesis of arynophiles	S8
Optimization	S11
Aryne captures using the iodonium precursor motif	S16
Aryne captures using the Kobayashi precursor motif	S21
Computational details	S27
Crystallography data	S41
References	S45
NMR spectra	S47

General experimental information

Unless otherwise stated, reagents and solvents were obtained commercially and used as provided by suppliers without further prior purification.

Solvents

MeCN was dried by distillation over CaH_2 and stored over molecular sieves (4 Å) under Ar atmosphere, or by passing it through a column of activated alumina under N_2 and stored over molecular sieves (4 Å) under N_2 atmosphere. THF and DCM when used as a reaction solvent were dried by being passed through a column of activated alumina under N_2 and stored over molecular sieves (4 Å) under a N_2 atmosphere.

Reagents

Bis(pinacolato)diboron, $B_2(pin)_2$, was dried in a round bottom flask lowered into an oil bath at 50 °C and under reduced pressure for 24 h and stored in a desiccator. CsF, KF, NaOt-Bu and *t*-BuOK were stored in a desiccator after opening. 18-Crown-6 was recrystallised from MeCN and stored in a desiccator.

Reaction conditions

Unless stated otherwise, all reactions were carried out under inert atmosphere N_2 with flame- or heat gun-dried glassware, under stirring with a magnetic stirrer.

Other conditions

All silica used was Silica gel 60 (40-63 μm) (VWR 84894.320), and unless otherwise stated was used as was. Flash column chromatography was performed with a Biotage® Isolera™ One flash chromatography system using Luknova® SuperSep™ cartridges. Filtrations were performed using sintered glass funnels by applying vacuum to the collection flask.

Analysis methods

Thin-layer chromatography

TLC analysis was performed using Merck TLC plates (TLC Silica gel 60, F_{254}, aluminium sheets). The plates were visualized under UV light (254 and 350 nm) and developed using $KMnO_4$ or vanillin staining solutions.

NMR Spectroscopy

General considerations

Unless otherwise specified, NMR spectra were recorded at 25 °C using an Agilent MR400-DD2 (400 MHz) or Bruker Avance Neo (500 MHz and 600 MHz) spectrometers. ^{1}H NMR chemical shifts were referenced to the appropriate residual solvent peaks (7.26 ppm for $CDCl_3$, 7.16 ppm for C_6D_6) and ^{13}C NMR chemical shifts were referenced to the appropriate solvent peaks (77.2 ppm for $CDCl_3$; 128.1 ppm for C_6D_6). ^{19}F NMR signals were referenced to external standard of $CFCl_3$ (0.0 ppm). Multiplicity of the NMR signals is reported as using the following abbreviations: singlet (s), doublet (d), triplet (t), doublet of doublets (dd), doublet of triplets (dt), quartet (q), multiplet (m). Non-product signals in the recorded NMR spectra were identified with the help of the reported chemical shifts of the common NMR impurities.[1] Spectra were processed with MNova software (v14.3.3). Auto baseline and auto phase corrections were applied. Structural assignments were made with additional information from gCOSY, gHSQC and gHMBC experiments.

For spectroscopic yields, an NMR spectrum was measured on an Agilent MR400-DD2 (400 MHz) spectrometer, using a relaxation delay of 10 s, collecting 32 transients.

Mass spectrometry

High-resolution accurate-mass mass spectra were analyzed using either a Thermo Fisher Scientific Orbitrap Elite (ESI), Waters Synapt G2-Si with Advion Nanomate (nanospray) or Thermo Fisher Scientific QExactive GC-Orbitrap (EI).

Crystallography

Single crystals were mounted on a fiber loop and fixated using Fomblin oil. The data were collected at 100 K on a Rigaku SynergyS using CuKa radiation (l = 1.5406 Å), respectively. Structures were solved by direct methods and refined by least squares methods on F2 using the SHELX and the OLEX2 software suites, respectively. All the non-hydrogen atoms were refined using an anisotropic model, and all the hydrogen atoms were constrained in geometrical positions to their parent atom. Crystallographic data are presented in Table S4 and S5. Deposition Numbers XYZXZY(5) and XZYXZY (9f) contain the supplementary crystallographic data for this paper. These data can be obtained free of charge via www.ccdc.cam.ac.uk/data_request/cif, or by emailing data_request@ccdc.cam.ac.uk, or by contacting The Cambridge Crystallographic Data Centre, 12 Union Road, Cambridge CB21EZ, UK; fax: +441223336033.

Synthesis of starting materials

5-(4,4,5,5-tetramethyl-1,3,2-dioxaborolan-2-yl)-2-(trimethylsilyl)phenyl trifluoromethanesulfonate (**7**)

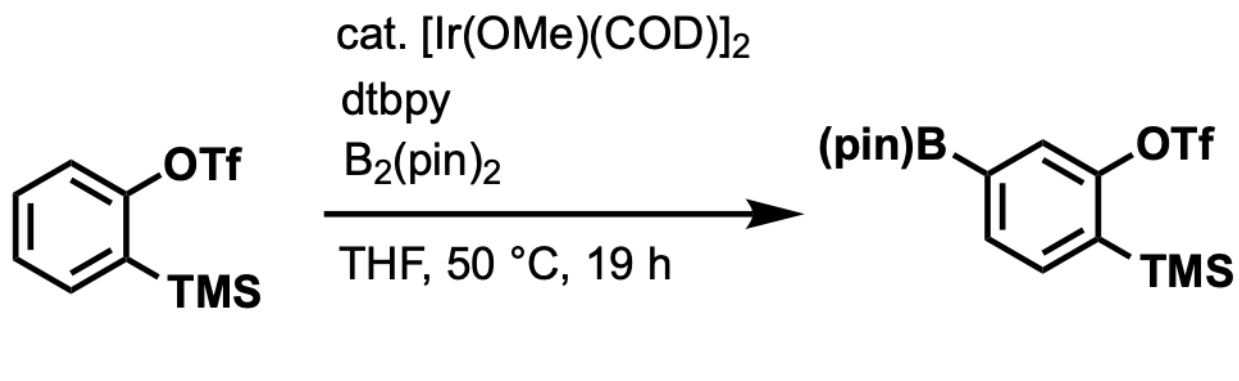

ESI-1 **7**

Modified literature procedure.[2] To a round-bottom flask (250 mL) were added $[Ir(OMe)(1,5\text{-}cod)]_2$ (301 mg, 0.455 mmol, 0.96 mol%), 4,4'-di-tert-butyl-2,2'-dipyridyl (dtbpy, 247 mg, 0.920 mmol, 1.93 mol%), $B_2(pin)_2$ (2.30 g, 9.09 mmol, 0.19 equiv.), and THF (90 mL). The mixture was stirred at 20 °C for 30 min. A colour change from yellow to deep red was observed. Compound **ESI-1** (14.20 g, 47.6 mmol, 1.00 equiv.) and additional $B_2(pin)_2$ (11.5 g, 45.3 mmol, 0.95 equiv.) were added, and the reaction mixture was stirred in an oil bath at 50 °C for 19 h. The reaction was allowed to cool to RT, after which Et_2O (50 mL) was added, and the solution was concentrated under reduced pressure. *n*-Pentane (50 mL) was added, and the solution was again concentrated under reduced pressure, to afford a thick red oil. The crude product was recrystallised from n-pentane twice to afford **7** (8.83g, 20.8 mmol, 44%) as a colourless solid. ^1H and ^{19}F NMR spectra were in accordance with literature values.[2b]

(3-(((trifluoromethyl)sulfonyl)oxy)-4-(trimethylsilyl)phenyl)boronic acid (**ESI-2a**)

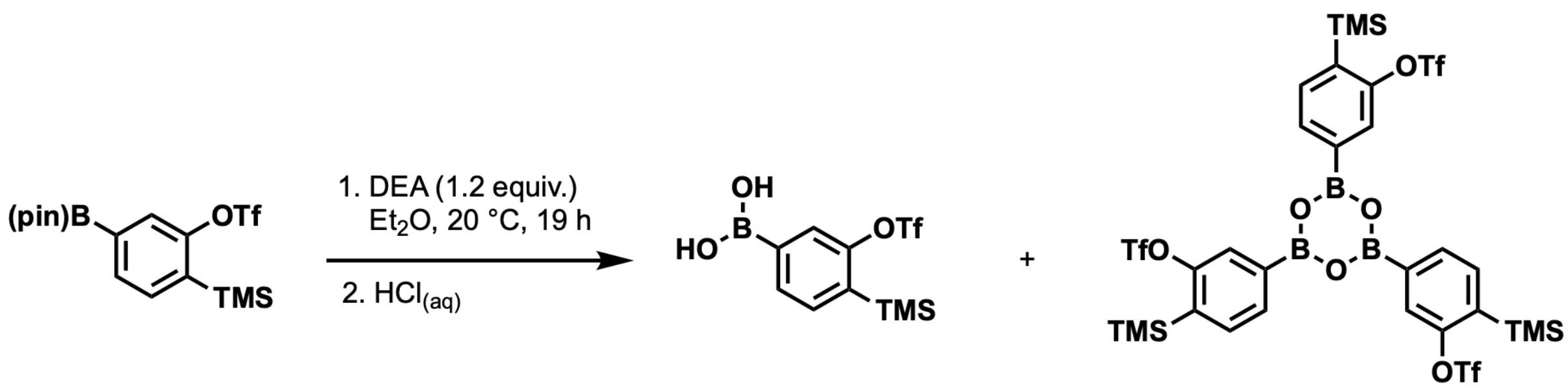

7 **ESI-2a** **ESI-2b**

This reaction was not performed under inert and dry conditions. Modified literature procedure.[2b]

To a round-bottom flask (250 mL) were added **7** (5.00 g, 11.7 mmol, 1.00 equiv.) and Et_2O (80 mL). Under stirring, diethanolamine (DEA, 1.36 mL, 1.49 g, 14.2 mmol, 1.20 equiv.) was added dropwise. A precipitate began to form. The reaction mixture was vigorously stirred at 20 °C for 19 h. The formed solid was filtered off and then redissolved in Et_2O (50 mL), to which aqueous HCl (1M, 50 mL) was added. The biphasic system was stirred until all solid dissolved (approx. 10 min). The phases were separated, and the aqueous phase was extracted with Et_2O (3 x 50 mL). The

combined organic phases were dried (Na_2SO_4), filtered and concentrated under reduced pressure to afford a mixture of **ESI-2a** and the corresponding boroxine **ESI-2b** as a colourless solid (3.53 g, 10.3 mmol based on **ESI-2a**, 88%). **ESI-2a** readily converts to **ESI-2b** upon drying.

Revised spectral data for **ESI-2a**:[2b]

^{1}H NMR (400 MHz, $CDCl_3$): δ 7.67 – 7.64 (m, 2H), 7.56 (d, *J* = 7.4 Hz, 1H), 4.69 (s, 2H), 0.36 (s, 9H).^{19}F NMR (376 MHz, $CDCl_3$): δ -73.9.

ESI-2b (boroxine – previously assigned as **ESI-2a**[2b]**):**

^{1}H NMR (400 MHz, $CDCl_3$): δ 8.15 – 8.05 (m, 2H), 7.72 (d, *J* = 7.2 Hz, 1H), 0.43 (s, 9H).^{19}F NMR (376 MHz, $CDCl_3$): δ -74.1.

(Diacetoxyiodo)mesitylene (**ESI-3**)

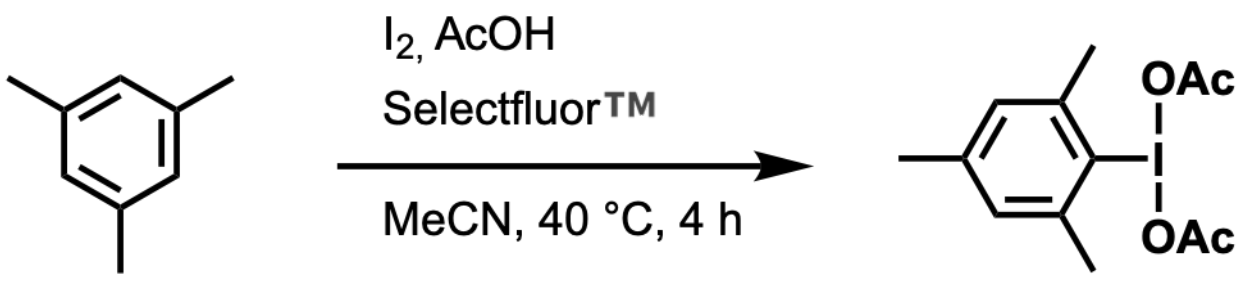

ESI-3

Modified literature procedure.[3]

To a 500 mL round bottom flask was added mesitylene (3.50 g, 29.1 mmol, 2.04 equiv.), MeCN (175 mL), acetic acid (32 mL, 34 g, 560 mmol, 38 equiv.), iodine (3.61 g, 14.2 mmol, 1.00 equiv.), and Selectfluor™ (25.0 g, 70.6 mmol, 4.96 equiv.). The mixture was lowered into an oil bath at 40 °C for 4 h and then concentrated under reduced pressure. Water (120 mL) and DCM (60 mL) were added, and the biphasic mixture was stirred until all solid had dissolved. The phases were separated, and the aqueous phase was extracted with DCM (3 x 60 mL). The combined organic phases were dried over Na_2SO_4, filtered, concentrated under reduced pressure, and triturated with Et_2O to afford **ESI-3** (8.71 g, 23.9 mmol, 82%) as a colourless solid. Spectra are in agreement with published values.[3]

Mesityl(3-(((trifluoromethyl)sulfonyl)oxy)-4-(trimethylsilyl)phenyl)-l3-iodaneyl 4-methylbenzenesulfonate (**8**)

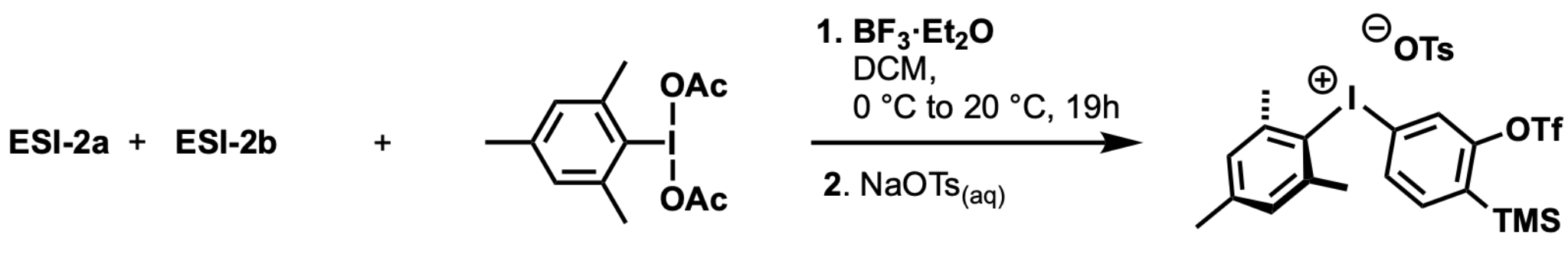

ESI-3 8

To a 500 mL round bottom flask were added **ESI-2** (3.50 g, mixture with boroxine, 10.2 mmol based on **ESI-2a**, 1.00 equiv.) and DCM (100 mL). The solution was cooled to 0 °C and boron trifluoride etherate (3.8 mL, 4.4 g, 31 mmol, 3.0 equiv.) was added dropwise.

In a separate 100 mL round bottom flask, **ESI-3** (4.10 g, 11.3 mmol, 1.10 equiv.) was dissolved in DCM (50 mL). This solution was transferred slowly to the solution of **ESI-2**, and the resulting mixture was stirred at 20 °C for 19 h. Aqueous NaOTs solution (2M, 103 mL, 206 mmol, 20.2 equiv.) was added, and the biphasic mixture was stirred at 20 °C for 10 min. The phases were separated and the aqueous phase was additionally extracted with DCM (4 × 50 mL). The combined organic phases were dried over Na_2SO_4, filtered, and concentrated under reduced pressure. The resulting crude product was triturated with Et_2O to afford **8** (5.07 g, 7.09 mmol, 69%) as a colourless solid.

Single crystals of **8** suitable for X-ray diffraction were obtained by slow evaporation of an ethyl acetate/n-pentane solution at room temperature.

^{1}H NMR (500 MHz, $CDCl_3$): δ 8.03 (dd, *J* = 8.0, 1.5 Hz, 1H), 7.46 (d, *J* = 8.0 Hz, 1H), 7.39 (d, *J* = 8.0 Hz, 2H), 7.20 (d, *J* = 1.5 Hz, 1H), 7.04 – 6.92 (m, 4H), 2.55 (s, 6H), 2.32 – 2.29 (m, 6H), 0.30 (s, 9H).

^{13}C{^{1}H} NMR (126 MHz, $CDCl_3$): δ 155.4, 143.9, 142.5, 142.4, 139.5, 138.6, 136.4, 132.6, 130.0, 128.5, 126.0, 122.6 (q, *J* = 1.4 Hz), 122.6, 118.3 (q, *J* = 320.5 Hz), 114.8, 27.1, 21.4, 21.2, -1.0.

^{19}F NMR (376 MHz, $CDCl_3$): δ -73.9.

HRMS (EI+): m/z calcd for $C_{19}H_{23}O_3SSiIF_3$ ($[M-OTs]^+$) 543.0134; found 543.0142

Synthesis of arynophiles

N-*tert*-Butyl-α-phenylnitrone (**10g**)

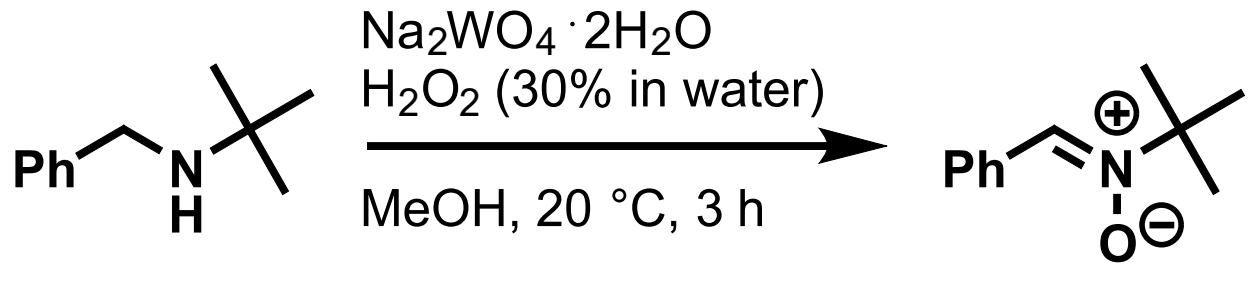

10g

Modified literature procedure.[4] This reaction was not performed under inert atmosphere or dry conditions.

To a 100 mL two-necked round-bottom flask was added N-Benzyl-*tert*-butylamine (4.11 g, 25.2 mmol, 1 equiv.), MeOH (40 mL), and $Na_2WO_4 \cdot 2H_2O$ (243 mg, 0.83 mmol, 3.0 mol%). Hydrogen peroxide (30% in water, 8.0 mL, 78 mmol, 3.1 equiv.) was added dropwise from an additional funnel over 10 min. The solution was stirred at 20 °C for 3 h, and was then concentrated under reduced pressure. The resulting solution was diluted with brine (40 mL) and EtOAc (30 mL). MnO_2 was added to quench remaining hydrogen peroxide. The phases were separated, and the aqueous phase was extracted with EtOAc (2 x 30 mL). The organic phases were combined, washed with brine (40 mL), dried (Na_2SO_4), filtered, and concentrated under reduced pressure to afford a crude product, which was recrystallised from hexanes to afford **10g** as a colourless crystalline solid (2.81 g, 15.9 mmol, 63 %). Spectral data were in agreement with literature values.[5]

(E)-2,4,6-trimethylbenzaldehyde oxime

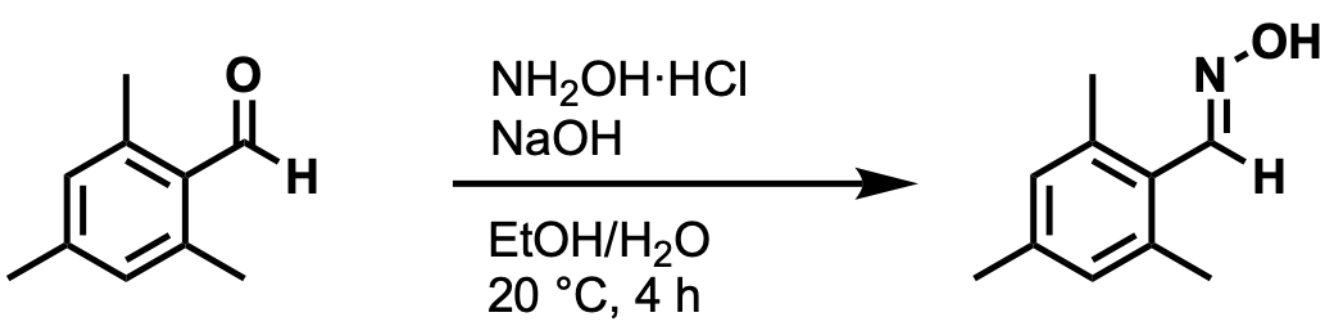

Modified literature procedure.[6] This reaction was not conducted under an inert atmosphere or dry conditions.

To a 100 mL round-bottom flask were added EtOH (20 mL), water (20 mL), mesitylaldehyde (6.17 g, 41.6 mmol, 1.0 equiv.), and hydroxylamine hydrochloride (4.29 g, 61.8 mmol, 1.48 equiv.). Stirring the resulting solution for 2 min led to the formation of a colourless solid.

NaOH (4.07 g, 102 mmol, 2.44 equiv.) was dissolved in water (10 mL), and added dropwise to the mesitylaldehyde/hydroxylamine mixture, which was then stirred at 20 °C for 4 h and poured into dilute hydrochloric acid (1M, 50 mL). A colourless solid formed, which was filtered off, dissolved in Et_2O (50 mL), and washed with dilute hydrochloric acid (1M, 50 mL). The washings were additionally extracted with Et_2O (2 x 50 mL), and the combined organic phases were dried (Na_2SO_4), filtered, and concentrated under reduced pressure to afford (E)-2,4,6-trimethylbenzaldehyde oxime as a colourless solid (6.01 g, 36.8 mmol, 88%). Spectral data were in agreement with previously reported values.[7]

Mesitylene nitrile oxide (**10f**)

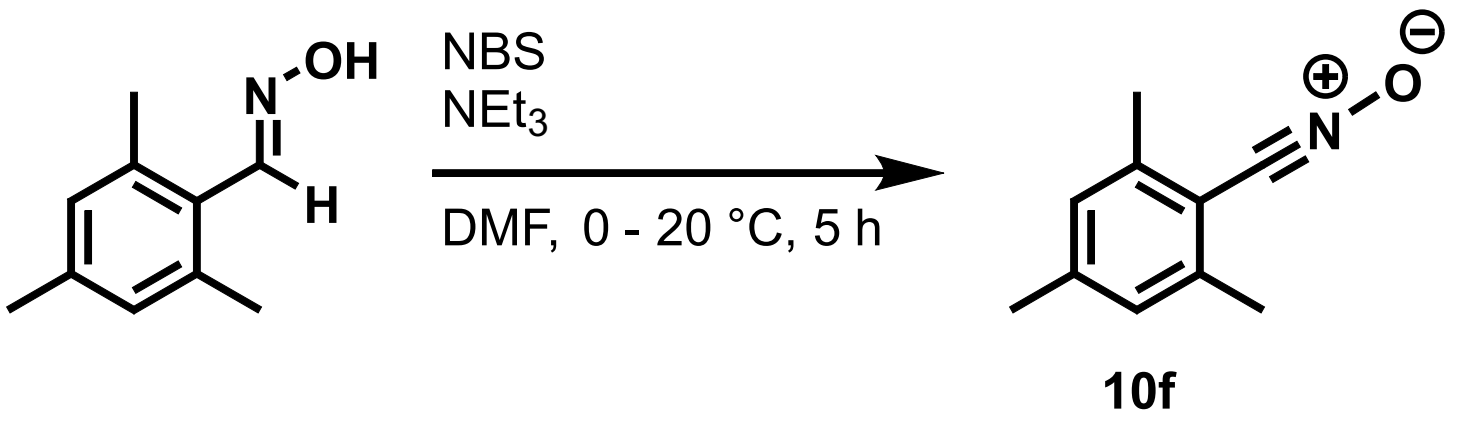

Modified version of a literature procedure.[8] This reaction was not conducted under an inert atmosphere or dry conditions.

To a 250 mL round-bottom flask were added DMF (30 mL) and (E)-2,4,6-trimethylbenzaldehyde oxime (5.01 g, 30.7 mmol, 1.00 equiv.). The solution was cooled to 0 °C. To a separate 100 mL round bottom flask were added DMF (30 mL) and N-bromosuccinimide (NBS, 6.05 g, 34.0 mmol, 1.11 equiv.). The NBS solution was added dropwise to the oxime solution, followed by dropwise addition of triethylamine (4.7 mL, 3.4 g, 34 mmol, 1.1 equiv.). A precipitate formed, making the solution viscous. The solution was taken off the ice bath and stirred for 1 h. It was then put back into the ice bath and stirred at 0 °C for 4 h. Water (100 mL) was added, and the formed precipitate was filtered off and recrystallised from methanol to afford **10f** as colourless needles (3.79 g, 23.5 mmol, 77%). Spectral data were in agreement with previously reported values.[8]

N-Nitroso-N-phenylglycine

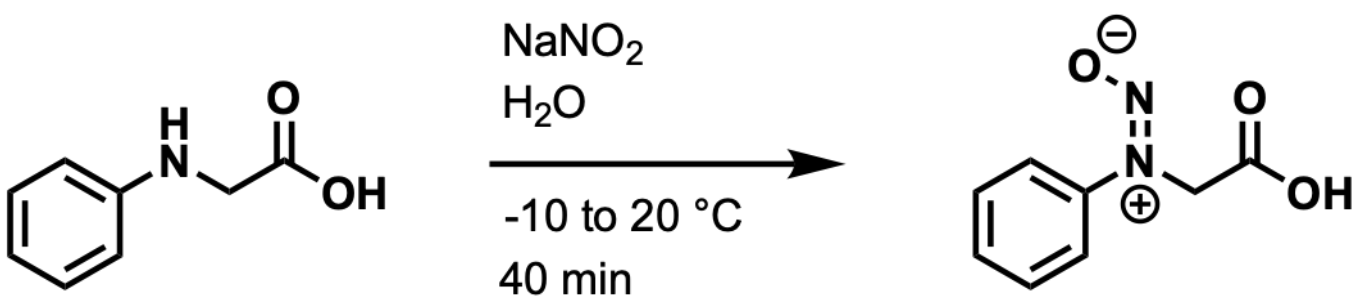

Modified literature procedure. This reaction was not conducted under an inert atmosphere or dry conditions.[9]

To a round-bottom flask (250 mL) was added *N*-phenylglycine (10.0g, 66.2 mmol, 1.00 equiv.) and water (120 mL). The flask was cooled to 0 °C. In a separate round-bottom flask (100 mL), $NaNO_2$ (5.00g, 72.5 mmol, 1.10 equiv.) was dissolved in water (30 mL). The $NaNO_2$ solution was transferred dropwise to the phenylglycine solution over 40 minutes. The solution was filtered, stirred with a small amount of activated carbon, and filtered. Concentrated HCl solution (10 mL) was added, and the formed dispersion was stirred for 10 minutes. It was filtered to afford *N*-nitroso-*N*-phenylglycine (7.60 g, 42.2 mmol, 64%) as a beige solid. Spectral data were in agreement with previously reported values.[10]

3-Phenylsydnone

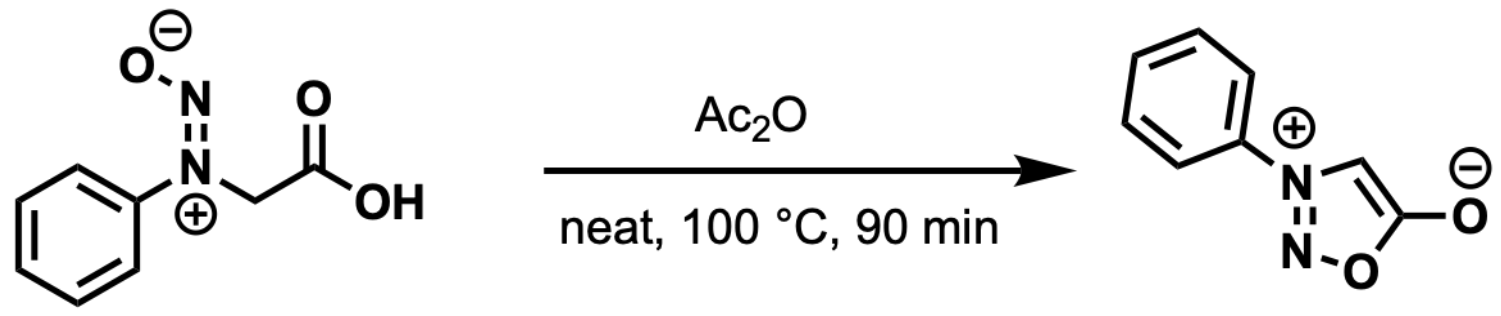

Modified literature procedure. This reaction was not conducted under an inert atmosphere or dry conditions.[9]

To a round-bottom flask (100 mL) was added N-nitroso-N-phenylglycine (7.60 g, 42.2 mmol, 1.00 equiv.) and acetic anhydride (40 mL). The mixture was stirred at 100 °C (oil bath) for 90 minutes. The solution was cooled to rt and poured into water (300 mL) under stirring. After 5 minutes, the formed solid was filtered off, washed with ice-cold water and dried to afford 3-phenylsydnone (5.86 g, 36.1 mmol, 86%) as a caramel-coloured solid. Spectral data were in agreement with previously reported values.[10]

Optimization

Design

The reaction optimisation was carried out with a fractional factorial experiment design.[11] The design was of resolution V, incorporating five factors at two levels, as well as three centerpoint runs, giving a total of 19 runs (Table S1).

For the reaction, the most relevant factors to optimise were determined to be the strength of the base, the counterion for the base, the reaction temperature and the solvent.

Table S1. *Design table used for the optimisation, with the runs shown in standard order and the levels of the factors given for each run. The design is balanced, as the levels for each factor sum to 0.*

Run	Base strength	Counterion	Temperature	Solvent parameter 1	Solvent parameter 2
1	-1	-1	1	-1	-1
2	1	-1	-1	-1	-1
3	-1	1	-1	-1	-1
4	1	1	1	-1	-1
5	-1	-1	-1	1	-1
6	1	-1	1	1	-1
7	-1	1	1	1	-1
8	1	1	-1	1	-1
9	-1	-1	-1	-1	1
10	1	-1	1	-1	1
11	-1	1	1	-1	1
12	1	1	-1	-1	1
13	-1	-1	1	1	1
14	1	-1	-1	1	1
15	-1	1	-1	1	1
16	1	1	1	1	1
17	0	0	0	0	0
18	0	0	0	0	0
19	0	0	0	0	0
Sum	0	0	0	0	0

Previously, Stuart and co-workers have shown that *tert*-butoxide bases perform better in similar reactions.[12] The use of acetate bases has been reported for the formation of cyclohexyne from cyclohexenyl iodonium salts by Fujita and co-workers.[13] As using a weaker base would allow the use of more base-sensitive arynophiles, and therefore increase the potential scope of the reaction, the bases compared in the optimisation are t-BuO$^-$ and AcO$^-$. Carbonate was used as the centerpoint base.

Due the difference in solubility of *tert*-butoxide and acetate bases with different solvents and reaction temperatures, the base counterion, the reaction temperature and the solvent were also included as factors. For the cations, potassium ions with 18-crown-6 and sodium ions were used as the high and low respectively. Potassium ions were used for the centerpoint. The solvents were parametrised using the results of the principal component analysis of reactions solvents performed by Sheppard and co-workers.[14] The first and second principal components, correlating to solvent polarity and polarizability respectively, were used for this optimisation. Toluene (PCA1 = 2.82, PCA2 = 0.65), MTBE (2.35, -2.35), DMF (-2.68, 0.53) and acetonitrile (-2.18, -1.49) were studied in the optimisation. 1,4-Dioxane (0.60, -0.20) was used as the centerpoint. The final design with experimental conditions including the measured spectroscopic yields is shown in Table S2. The run order was randomised to reduce the risk of introducing systematic errors.

Table S2. *Design table used for the optimisation, with the runs shown in standard order and the conditions used for each run, as well as the spectroscopic yield measured for the run.*

Run	Base strength	Counterion	Temperature	Solvent	Spectroscopic yield 11b (%)
1	$^{-}$OAc	Na^+	60°C	MeCN	5
2	*t*-BuO^{-}	Na^+	20°C	MeCN	51
3	$^{-}$OAc	K^+ + 18-crown-6	20°C	MeCN	2
4	*t*-BuO^{-}	K^+ + 18-crown-6	60°C	MeCN	37
5	$^{-}$OAc	Na^+	20°C	MTBE	0
6	*t*-BuO^{-}	Na^+	60°C	MTBE	55
7	$^{-}$OAc	K^+ + 18-crown-6	60°C	MTBE	17
8	*t*-BuO^{-}	K^+ + 18-crown-6	20°C	MTBE	37
9	$^{-}$OAc	Na^+	20°C	DMF	0
10	*t*-BuO^{-}	Na^+	60°C	DMF	0
11	$^{-}$OAc	K^+ + 18-crown-6	60°C	DMF	0
12	*t*-BuO^{-}	K^+ + 18-crown-6	20°C	DMF	0
13	$^{-}$OAc	Na^+	60°C	Toluene	4
14	*t*-BuO^{-}	Na^+	20°C	Toluene	51
15	$^{-}$OAc	K^+ + 18-crown-6	20°C	Toluene	2
16	*t*-BuO^{-}	K^+ + 18-crown-6	60°C	Toluene	29
17	CO_3^{2-}	Na^+	20°C	1,4-Dioxane	3
18	CO_3^{2-}	Na^+	40°C	1,4-Dioxane	32
19	CO_3^{2-}	K^+ + 18-crown-6	40°C	1,4-Dioxane	2

Reaction conditions: **8** (71 mg, 0.1 mmol, 1.0 equiv.), **10b** (32.5 μL, 28.9 mg, 0.30 mmol, 3.0 equiv.), base (0.15 mmol, 1.5 equiv., 1.6 equiv. for *t*-BuOK), solvent (2.5 mL), temperature, 40 min. All heating conducted in a conventional oil bath.

Results

As the design was of Resolution V, the main effects and the two-factor interactions of the system were separated from each other. The main effects are aliased with the four-factor interactions, while the two-factor interactions are aliased with the three-factors interactions. Both the three-factor and four-factor interactions were assumed to be negligible.

Out of the main effects (Figure S1), the strength of the base is the most important effect, strongly favouring the use of *tert*-butoxide bases. There is a negative effect from the use of potassium counterions together with 18-crown-6 compared to using sodium ions. The reaction temperature alone has no effect on the reaction. Looking at the solvent, the reaction favours low-polarity (high PCA1 corresponds to low polarity solvents) and low-polarizability solvents, in this study MTBE.

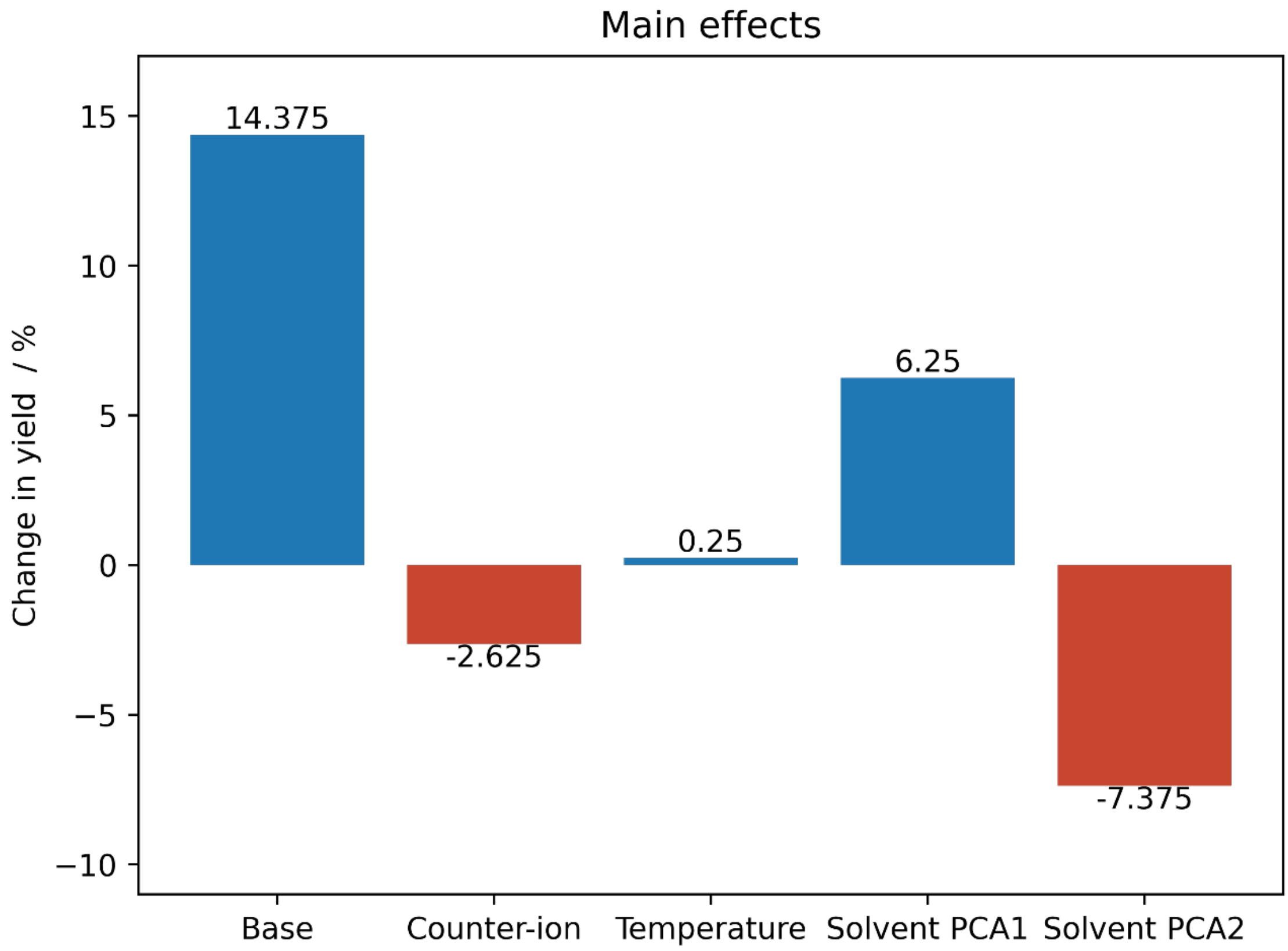

Figure S1. *Plot of reaction main effects.*

Looking at the two-factor interactions (Figure S2), the reaction has a strong negative interaction between the base strength and the counter-ion, further favouring the use of NaOt-Bu. Strong bases favourably interacts with low reaction temperature, and the use of MTBE as the reaction solvent. Other notable interactions are the positive interaction between counterion and temperature, the negative interaction between temperature and solvent polarizability, and the positive interaction between solvent polarity and polarizability.

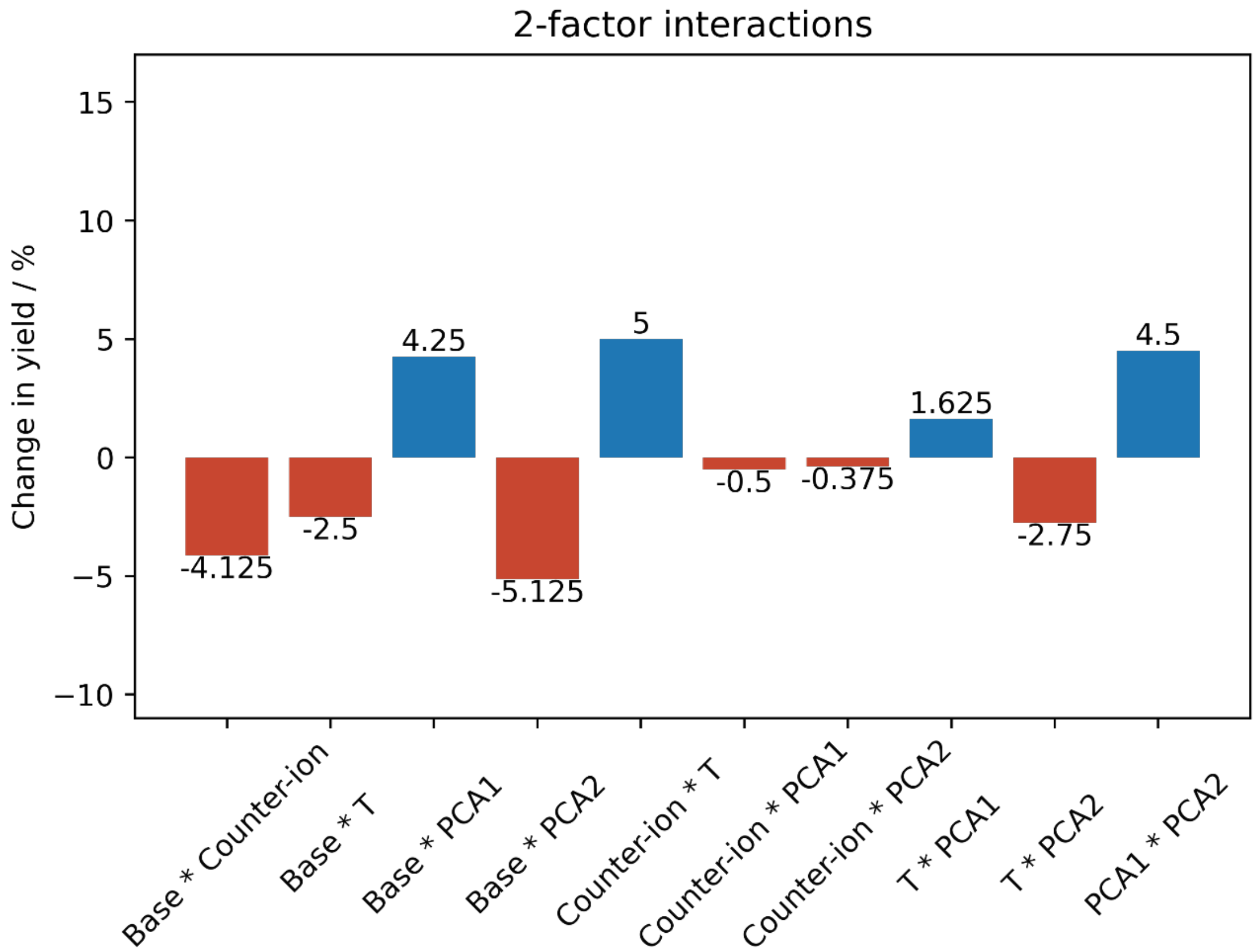

Figure S2. *Plot of reaction two-factor interactions.*

Based on these results, the optimised conditions were found to consist of NaO*t*-Bu as the base, a reaction temperature of 20 °C in MTBE as solvent, with a predicted yield of 61% (Table S3). Performing the reaction using the optimised conditions gave 34% yield. However, switching from using *t*-BuONa to *t*-BuOK resulted in a yield of 69%. Reducing the loading of **10b** from 3.0 equiv. to 2.0 equiv. retained essentially the same yield (70%).

Table S3. *Test of optimised conditions.*

Entry	**10b** (equiv.)	Base	Yield **11b** (%)
1	3.0	*t*-BuONa	61[a]
2	3.0	*t*-BuONa	34
3	2.0	*t*-BuONa	68
4	3.0	*t*-BuOK	69
5	2.0	*t*-BuOK	70

Optimised conditions: **8** (71 mg, 0.1 mmol, 1.0 equiv.), **10b** (32.5 µL, 28.9 mg, 0.30 mmol, 3.0 equiv.), *t*-BuONa (14.4 mg, 0.15 mmol, 1.5 equiv.), MTBE (2.5 mL), 20 °C, 40 min. [a] Predicted yield from optimisation

Aryne captures using the iodonium precursor motif

General procedure (GP) for aryne captures using precursor **8**

To a microwave vial (Biotage, 10 mL) was added **8** (0.20 mmol, 1.0 equiv.), MTBE (5 mL, 0.04 M), **10** (0.40 mmol, 2.0 equiv.) and t-BuOK (36.7, 1.6 equiv.). The initially heterogenous mixture was stirred at 20 °C for 40 min. The reaction mixture was then added to brine (5 mL). The biphasic mixture was extracted with EtOAc (3 x 10 mL), the combined organic phases were dried (Na_2SO_4), filtered and concentrated under reduced pressure. Purification by column chromatography afforded the pure product.

Aryne capture products (Scheme 2)

6-(Trimethylsilyl)-1,4-dihydro-1,4-epoxynaphthalen-5-yl trifluoromethanesulfonate (**11a**)

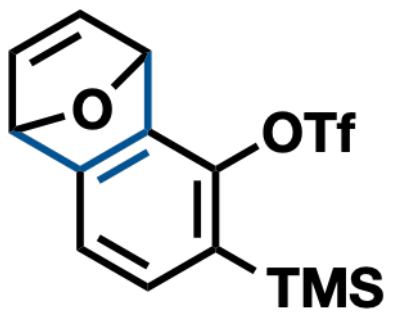

11a

General procedure GP. **10a** was used as the arynophile. Purification by column chromatography using a 1:9 EtOAc/hexanes mobile phase. Isolated as a yellow oil (47 mg, 0.13 mmol, 65%)

^{1}H NMR (400 MHz, $CDCl_3$): δ 7.25 (d, *J* = 7.0 Hz, 1H), 7.21 (d, *J* = 7.0 Hz, 1H), 7.18 – 7.12 (m, 1H), 7.08 – 6.99 (m, 1H), 6.09 – 5.87 (m, 1H), 5.80 – 5.66 (m, 1H), 0.33 (s, 9H).

^{13}C{^{1}H} NMR (101 MHz, $CDCl_3$): δ 155.0, 145.6, 143.0, 142.5, 140.6, 135.2, 130.9, 119.8, 118.6 (q, *J* = 319.4 Hz), 82.7, 81.6, -0.2.

^{19}F NMR (376 MHz, $CDCl_3$): δ -73.8.

HRMS (ESI+): m/z calcd for $C_{14}H_{15}O_4SSiF_3Na$ ([M+Na]$^+$) 387.0310; found 387.0318

1,4-Dimethyl-6-(trimethylsilyl)-1,4-dihydro-1,4-epoxynaphthalen-5-yl trifluoromethanesulfonate (**11b**)

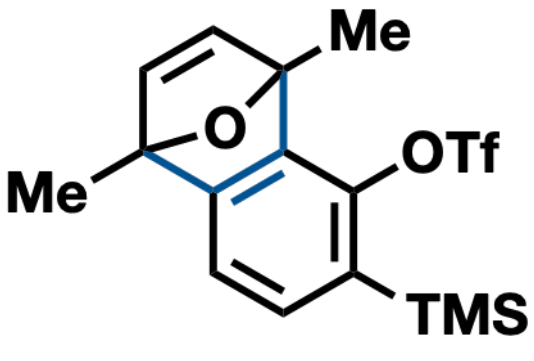

11b

General procedure GP. **10b** was used as the arynophile. Reaction performed using 0.3 mmol **8**. Purification by column chromatography using a 1:9 EtOAc/hexanes mobile phase. Isolated as a yellow oil (84 mg, 0.21 mmol, 71%).

^{1}H NMR (400 MHz, $CDCl_3$): δ 7.24 (d, *J* = 7.0 Hz, 1H), 7.14 (d, *J* = 7.0 Hz, 1H), 6.96 (d, *J* = 5.3 Hz, 1H), 6.74 (d, *J* = 5.3 Hz, 1H), 1.99 (s, 3H), 1.89 (s, 3H), 0.33 (s, 9H).

^{13}C{^{1}H} NMR (101 MHz, $CDCl_3$): δ 159.0, 146.6, 146.0, 145.7, 143.2, 135.6, 132.3, 118.6 (q, *J* = 319.6 Hz), 118.2, 90.5, 88.4, 16.5, 15.4, 0.5.

^{19}F NMR (376 MHz, $CDCl_3$): δ -73.3.

HRMS (EI+): m/z calcd for $C_{16}H_{19}O_4SSiF_3$ ($[M]^+$) 392.0720; found 392.0723

2-Bromo-6-(trimethylsilyl)-1,4-dihydro-1,4-epoxynaphthalen-5-yl trifluoromethanesulfonate (**11c** and **11c'**)

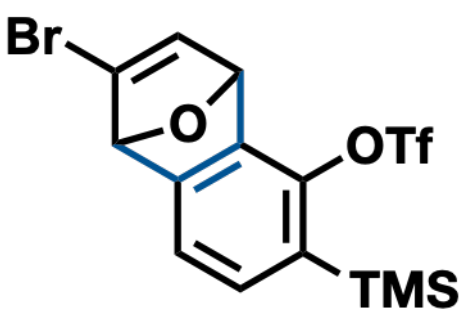

11c

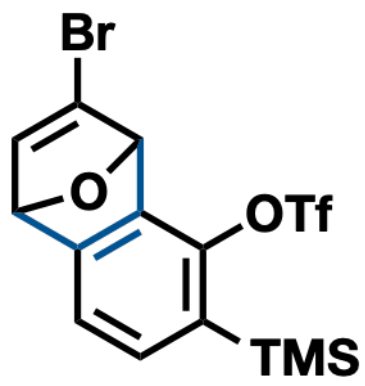

11c'

General procedure GP. **10c** was used as the arynophile. Reaction performed using 1.0 mmol of **8**. Purification by column chromatography using a EtOAc/hexanes gradient mobile phase. Regioselectivity determined by ^{1}H NMR analysis of the crude mixture: **11c**/**11c'** = 3.1:1 *rr*.

11c
Isolated as a yellow oil (223 mg, 0.50 mmol, 50%).

^{1}H NMR (500 MHz, $CDCl_3$): δ 7.37 (d, *J* = 7.0 Hz, 1H), 7.28 – 7.25 (m, 1H), 7.06 (d, *J* = 2.0 Hz, 1H), 5.94 (dd, *J* = 2.2, 1.1 Hz, 1H), 5.49 (d, *J* = 1.1 Hz, 1H), 0.34 (s, 9H).

^{13}C{^{1}H} NMR (126 MHz, $CDCl_3$): δ 152.8, 145.3, 139.6, 139.2, 136.0, 135.5, 132.2, 120.3, 118.6 (q, *J* = 319.3 Hz), 87.2, 83.7, -0.2.

^{19}F NMR (376 MHz, $CDCl_3$): δ -73.8.

HRMS (ESI+): m/z calcd for $C_{14}H_{14}O_4SSiBrF_3Na$ ($[M+Na]^+$) 464.9415; found 464.9414

11c'
Isolated as a yellow oil (57 mg, 0.13 mmol, 13%).

^{1}H NMR (500 MHz, $CDCl_3$): δ 7.29 (d, *J* = 7.0 Hz, 1H), 7.27 – 7.24 (m, 1H), 7.03 (d, *J* = 2.1 Hz, 1H), 5.89 (d, *J* = 1.0 Hz, 1H), 5.75 (dd, *J* = 2.1, 1.1 Hz, 1H), 0.35 (s, 9H).

^{13}C{^{1}H} NMR (126 MHz, $CDCl_3$): δ 153.6, 146.1, 140.4, 138.8, 136.3, 136.1, 132.1, 119.9, 118.6 (q, *J* = 319.7 Hz), 85.8, 84.5, -0.2.

^{19}F NMR (376 MHz, $CDCl_3$): δ -73.7.

HRMS (ESI+): m/z calcd for $C_{14}H_{14}O_4SSiBrF_3Na$ ($[M+Na]^+$) 464.9415; found 464.9423

9,10-Diphenyl-2-(trimethylsilyl)-9,10-dihydro-9,10-epoxyanthracen-1-yl trifluoromethanesulfonate (**11d**)

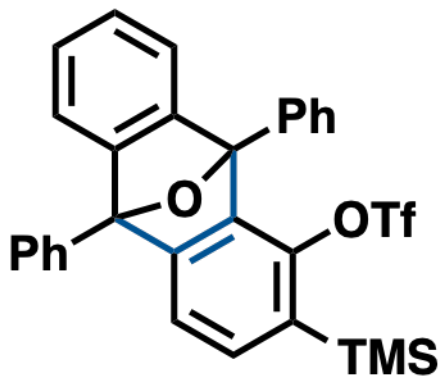

11d

Modified general procedure GP. **10d** was used as the arynophile. Reaction performed using 1.0 mmol **8**. The compound was purified by flash column chromatography using a hexanes/toluene gradient mobile phase. Compound **11d** was afforded as a green solid (359 mg, 0.63 mmol, 63%).

^{1}H NMR (500 MHz, $CDCl_3$): δ 7.89 (dd, *J* = 7.9, 1.8 Hz, 2H), 7.85 (d, *J* = 7.4 Hz, 1H), 7.82 (dd, *J* = 8.3, 1.3 Hz, 2H), 7.66 – 7.39 (m, 8H), 7.36 (d, *J* = 7.2 Hz, 1H), 7.22 (td, *J* = 7.5, 1.1 Hz, 1H), 7.14 (td, *J* = 7.4, 1.1 Hz, 1H), 0.35 (s, 9H).

^{13}C{^{1}H} NMR (126 MHz, $CDCl_3$): δ 157.6, 150.7, 147.0, 143.8, 142.7, 136.5, 134.3, 133.7, 133.4, 129.8, 129.6, 128.9, 128.6, 126.9, 126.2, 126.1, 125.4, 120.5, 120.4, 118.0 (q, *J* = 320.2 Hz), 93.4, 90.2, 0.4.

^{19}F NMR (376 MHz, $CDCl_3$): δ -73.8.

HRMS (ESI+): m/z calcd for $C_{30}H_{25}O_4SSiF_3Na$ ($[M+Na]^+$) 589.1093; found 589.1083

9-Phenyl-6-(trimethylsilyl)-1,4-dihydro-1,4-epiminonaphthalen-5-yl trifluoromethanesulfonate (**11e**)

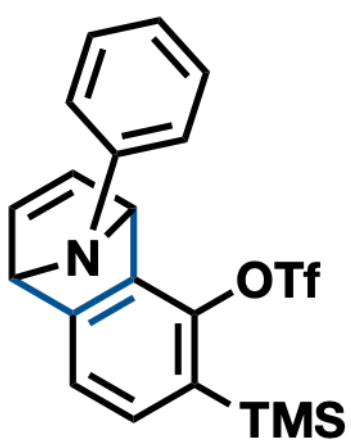

11e

General procedure GP. **10e** was used as the arynophile. Purification by column chromatography using a 1:9 EtOAc/hexanes mobile phase. Compound **11e** was isolated as a black solid (41 mg, 0.094 mmol, 47%).

^{1}H NMR (500 MHz, $CDCl_3$): δ 7.25 (d, *J* = 7.4 Hz, 1H), 7.24 – 7.16 (m, 2H), 7.16 (d, *J* = 7.0 Hz, 1H), 7.06 (dd, *J* = 5.4, 2.4 Hz, 1H), 6.98 (dd, *J* = 5.4, 2.5 Hz, 1H), 6.87 (tt, *J* = 7.3, 1.1 Hz, 1H), 6.84 – 6.77 (m, 2H), 5.72 (t, *J* = 2.0 Hz, 1H), 5.49 (t, *J* = 2.1 Hz, 1H), 0.31 (s, 9H).

^{13}C{^{1}H} NMR (126 MHz, $CDCl_3$): δ 154.6, 147.0, 146.1, 141.8, 141.7, 140.3, 134.9, 130.6, 129.1, 121.4, 121.0, 118.7 (q, *J* = 319.3 Hz), 117.9, 69.6, 68.2, -0.2.

^{19}F NMR (376 MHz, $CDCl_3$): δ -73.7.

HRMS (ESI+): m/z calcd for $C_{20}H_{21}O_3SSiNF_3$ ($[M+H]^+$) 440.0964; found 440.0952

3-Mesityl-5-(trimethylsilyl)benzo[d]isoxazol-4-yl trifluoromethanesulfonate (**11f**)

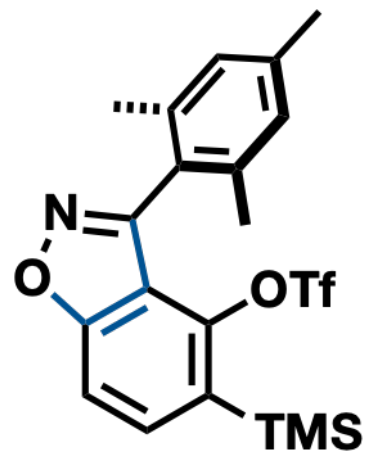

11f

General procedure GP. **10f** was used as the arynophile. Purification by column chromatography using a 1:9 EtOAc/hexanes mobile phase. Compound **11f** was obtained as a yellow solid (53 mg, 0.13 mmol, 58%). Only the expected major regioisomer was observed to form.

Single crystals of **11f** suitable for X-ray diffraction were obtained by slow evaporation of an ethyl acetate/n-pentane solution at room temperature.

^{1}H NMR (400 MHz, $CDCl_3$): δ 7.78 (d, *J* = 8.6 Hz, 1H), 7.72 (d, *J* = 8.6 Hz, 1H), 6.96 (s, 2H), 2.34 (s, 3H), 2.11 (s, 6H), 0.41 (s, 9H).

^{13}C{^{1}H} NMR (101 MHz, $CDCl_3$): δ 142.8, 139.6, 138.2, 137.7, 130.1, 128.6, 123.7, 118.0 (q, *J* = 321.1 Hz), 117.5, 110.9, 21.3, 20.3, 0.4.

^{19}F NMR (376 MHz, $CDCl_3$): δ -73.3.

HRMS (ESI+): m/z calcd for $C_{20}H_{23}O_4SSiNF_3$ ([M+H]$^+$) 458.1064; found 458.1057

2-(tert-butyl)-3-phenyl-5-(trimethylsilyl)-2,3-dihydrobenzo[d]isoxazol-4-yl trifluoromethanesulfonate (**11g**)

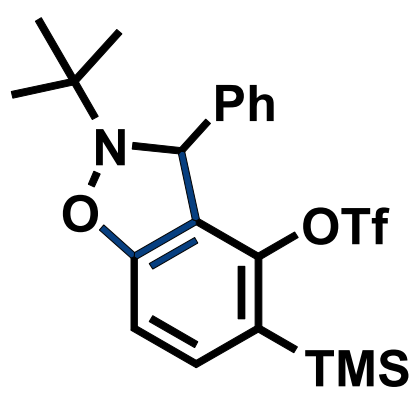

11g

General procedure GP. **10g** was used as the arynophile. Compound **11g** was isolated as a yellow oil (69 mg, 0.15 mmol, 73%). Only the expected major regioisomer was observed to form.

^{1}H NMR (400 MHz, $CDCl_3$): δ 7.43 (d, *J* = 8.1 Hz, 1H), 7.35 – 7.21 (m, 3H), 7.19 – 7.12 (m, 2H), 6.93 (d, *J* = 8.1 Hz, 1H), 6.00 (s, 1H), 1.22 (s, 9H), 0.30 (s, 9H).

^{13}C{^{1}H} NMR (101 MHz, $CDCl_3$): δ 161.7, 146.5, 141.5, 138.3, 128.8, 127.9, 127.4, 125.8, 123.0, 116.8 (q, *J* = 319.9 Hz), 107.5, 65.5, 62.2, 25.6, 0.1.

^{19}F NMR (376 MHz, $CDCl_3$): δ -73.5.

HRMS (ESI+): m/z calcd for $C_{21}H_{27}O_4SSiNF_3$ ([M+H]$^+$) 474.1377; found 474.1384

1-benzyl-5-(trimethylsilyl)-1H-benzo[d][1,2,3]triazol-4-yl trifluoromethanesulfonate (**11h**)

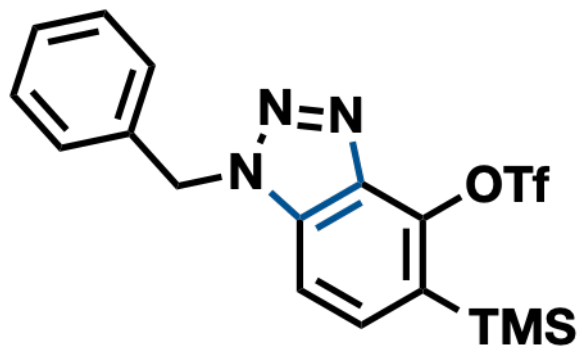

11h

General procedure GP. **10h** was used as the arynophile. Purification by column chromatography using a EtOAc/hexanes gradient mobile phase. Compound **11h** was afforded as a peach coloured solid (29 mg, 0.068 mmol, 34%). Only the expected major regioisomer was observed to form.

^{1}H NMR (400 MHz, $CDCl_3$) δ 7.50 (d, J = 8.3 Hz, 1H), 7.38 – 7.28 (m, 6H), 5.84 (s, 2H), 0.43 (s, 9H).

^{1}H NMR (400 MHz, C_6D_6) δ 7.02 (d, J = 8.3 Hz, 1H), 6.97 – 6.86 (m, 3H), 6.82 – 6.73 (m, 2H), 6.62 (d, J = 8.3 Hz, 1H), 5.02 (s, 2H), 0.37 (s, 9H).

^{13}C{^{1}H} NMR (101 MHz, $CDCl_3$) δ 143.9, 139.3, 136.2, 134.2, 133.8, 129.3, 128.9, 128.4, 127.9, 117.4 (q, J = 322.1 Hz), 109.9, 52.9, -0.2.

^{19}F NMR (376 MHz, $CDCl_3$) δ -71.1.

HRMS (ESI+): m/z calcd for $C_{17}H_{19}O_3SSiN_3F_3$ ($[M+H]^+$) 430.0863; found 430.0866

3-hydroxy-2-(pyridin-3-yl)-6-(trimethylsilyl)phenyl trifluoromethanesulfonate (**11i**)

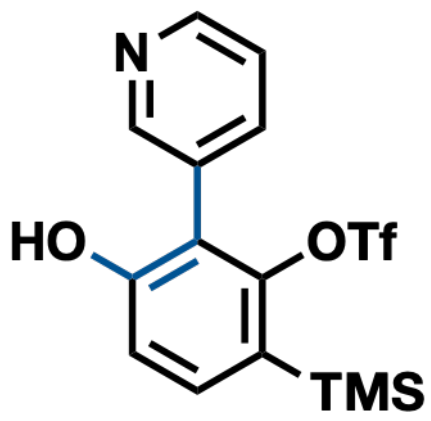

11i

General procedure GP. **10i** was used as the arynophile. Purification by column chromatography using a EtOAc/hexanes gradient mobile phase. Compound **11i** was obtained as a yellow solid (23 mg, 0.058 mmol, 29%). Regioselectivity (**11i**:**11i’**): at least ≥3.7:1 (based on ratio of the peak of **11i** and the second largest peak in the ^{19}F NMR spectrum of the crude product).

^{1}H NMR (400 MHz, $CDCl_3$): δ 10.28 (broad s, 1H), 8.59 (d, J = 1.8 Hz, 1H), 8.48 (dd, J = 5.0, 1.5 Hz, 1H), 7.83 (dt, J = 8.1, 1.8 Hz, 1H), 7.53 – 7.34 (m, 2H), 7.06 (d, J = 8.3 Hz, 1H), 0.38 (s, 9H).

^{13}C{^{1}H} NMR (101 MHz, $CDCl_3$): δ 157.9, 151.1, 150.3, 147.3, 140.2, 137.3, 129.7, 124.7, 123.8, 119.8, 118.1 (q, J = 320.3 Hz), 116.4, 0.3.

^{19}F NMR (376 MHz, $CDCl_3$) δ -74.25.

HRMS (ESI-): m/z calcd for $C_{15}H_{15}O_4SSiNF_3$ ($[M-H]^-$) 390.0443; found 390.0436

Aryne captures using the Kobayashi precursor motif

Products (Scheme 3)

7-Bromo-2-phenyl-6,9-dihydro-2H-6,9-epoxybenzo[e]indazole (**13a** and **13a'**)

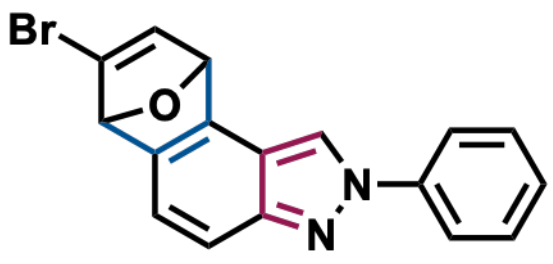

13a

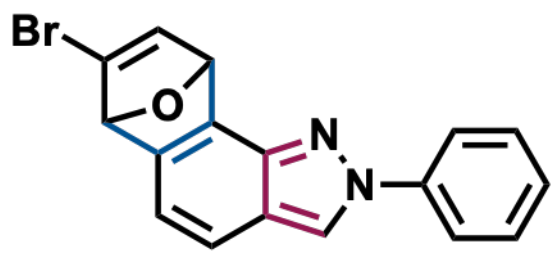

13a'

To a 10 mL microwave vial were added **11c** (44 mg, 0.10 mmol, 1.0 equiv.), 3-phenylsydnone (24 mg, 0.15, 1 equiv.), and tetrabutylammonium fluoride hydrate (36 mg, 0.13 mmol, 1.3 equiv.). The vial was sealed under N_2 and THF (1 mL) was added. The reaction mixture was stirred at 20 °C for 18 h. The reaction mixture was diluted with water (20 mL) and extracted with EtOAc (3 x 20 mL). The combined organic phases were washed with brine (10 mL), dried (Na_2SO_4), filtered and concentrated under reduced pressure. The crude product was purified by flash chromatography (EtOAc/hexanes gradient) to afford **13a** as a colourless solid and **13a'** (7.8 mg, 0.023 mmol, 23%) as a yellow oil.

13a

Colourless solid (12 mg, 0.035 mmol, 35%).

^{1}H NMR (500 MHz, $CDCl_3$): δ 8.42 (d, *J* = 1.0 Hz, 1H), 7.98 – 7.84 (m, 2H), 7.61 – 7.52 (m, 4H), 7.47 – 7.42 (m, 1H), 7.09 (d, *J* = 2.0 Hz, 1H), 6.07 (dd, *J* = 2.0, 1.1 Hz, 1H), 5.62 (d, *J* = 1.1 Hz, 1H).

^{13}C{^{1}H} NMR (126 MHz, $CDCl_3$): δ 150.0, 143.3, 142.4, 140.6, 139.9, 139.4, 129.8, 128.6, 121.4, 120.9, 118.3, 117.7, 114.9, 88.2, 84.5.

HRMS (ESI+): m/z calcd for $C_{17}H_{12}N_2OBr$ ([M+H]$^+$): 339.0128; found 339.0127

13a'

Yellow oil (7.8 mg, 0.023 mmol, 23%).

^{1}H NMR (500 MHz, $CDCl_3$): δ 8.46 (s, 1H), 8.01 – 7.82 (m, 2H), 7.65 – 7.52 (m, 3H), 7.49 – 7.41 (m, 1H), 7.37 (d, *J* = 8.3 Hz, 1H), 7.16 (d, *J* = 2.1 Hz, 1H), 6.43 – 6.31 (m, 2H), 5.62 (d, *J* = 1.1 Hz, 1H).

^{13}C{^{1}H} NMR (126 MHz, $CDCl_3$): δ 148.8, 144.4, 140.6, 140.2, 139.1, 138.4, 129.8, 128.5, 123.7, 121.6, 121.6, 118.5, 116.9, 88.3, 83.7.

HRMS (ESI+): m/z calcd for $C_{17}H_{12}N_2OBr$ ([M+H]$^+$) 339.0128; found 339.0128

3-Bromo-N-(4-iodophenyl)-1,4-dihydro-1,4-epoxynaphthalen-6-amine (**13b**)

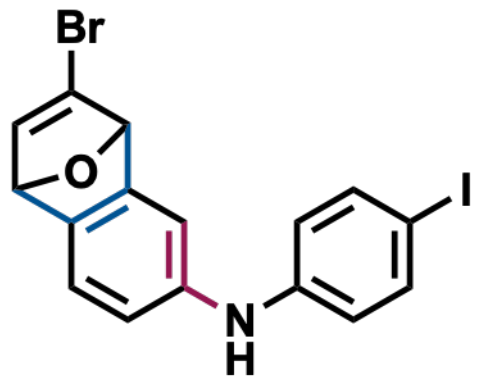

13b

To a 10 mL microwave vial were added **11c'** (44 mg, 0.10 mmol, 1.0 equiv.), 4-iodoaniline (33 mg, 0.15 mmol, 1.5 equiv.), CsF (76 mg, 0.50 mmol, 5.0 equiv.) and 18-crown-6 (78 mg, 0.30 mmol, 3.0 equiv.). The vial was sealed under N_2 and MeCN (1 mL) was added. The reaction mixture was stirred at 20 °C for 18 h. The reaction mixture was diluted with water (10 mL) and extracted with EtOAc (3 x 10 mL). The combined organic phases were washed with brine (10 mL), dried (Na_2SO_4), filtered and concentrated under reduced pressure. The crude product was purified by flash chromatography (EtOAc/hexane gradient) gradient to afford **13b** as a brown solid (14 mg, 0.033 mmol, 33%). Regioselectivity (**13b**:**13b'**): 7:1 (doublet at 7.61 ppm in the crude ^{1}H NMR spectrum was assumed to belong to **13b'** and corresponds to one proton. As this doublet is the largest unassigned peak in the aromatic region, this is a lower bound for the regioselectivity.)

^{1}H NMR (500 MHz, $CDCl_3$) δ 7.54 – 7.49 (m, 2H), 7.18 – 7.11 (m, 2H), 6.95 (d, *J* = 2.0 Hz, 1H), 6.81 – 6.77 (m, 2H), 6.69 (dd, *J* = 7.6, 2.0 Hz, 1H), 5.71 (d, *J* = 2.1 Hz, 1H), 5.67 (s, 1H), 5.40 (s, 1H).

^{13}C{^{1}H} NMR (126 MHz, $CDCl_3$) δ 149.0, 143.3, 140.7, 140.3, 139.9, 138.3, 136.0, 120.9, 119.5, 114.8, 113.5, 87.1, 84.3, 82.4.

HRMS (ESI+): m/z calcd for $C_{16}H_{12}ONBrI$ ($[M+H]^+$) 439.9147; found 439.9143

Ethyl 6,11-diphenyl-6,11-dihydro-1H-6,11-epoxynaphtho[2,3-g]indazole-3-carboxylate (**13c**)

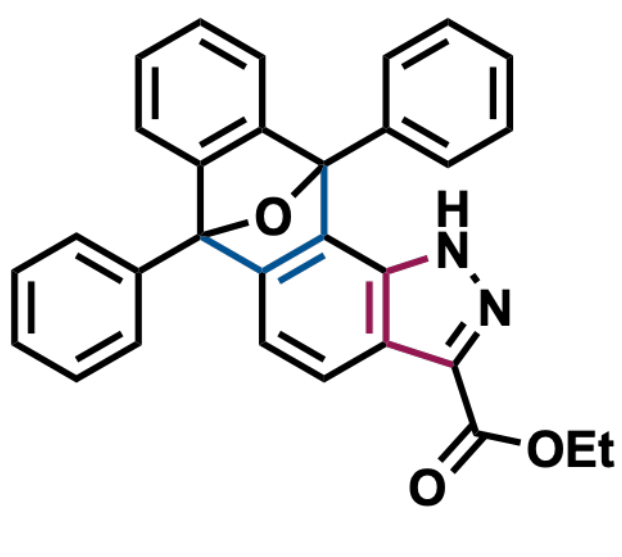

13c

To a 10 mL microwave vial was added **11d** (56 mg, 0.10 mmol, 1.0 equiv.), KF (18 mg, 0.30 mmol, 3.0 equiv.), and 18-crown-6 (78 mg, 0.30 mmol, 3.0 equiv.). The vial was sealed under N_2 and diazoacetic acid ethyl ester (87% in DCM, 18 µL, 20 mg, 0.15 mmol, 1.5 equiv.) and THF (1 mL) was added. The reaction mixture was stirred at 20 °C for 18 h. The reaction mixture was diluted with water (10 mL) and extracted with EtOAc (3 x 10 mL). The combined organic phases were

washed with brine (10 mL), dried over Na_2SO_4, filtered and concentrated under reduced pressure. The crude product was purified by flash chromatography (EtOAc/hexane gradient) to afford **13c** as a colourless solid (25 mg, 0.054 mmol, 55%).

No other regioisomer was observed in the crude ^{1}H NMR spectrum.

^{1}H NMR (500 MHz, $CDCl_3$) δ 10.19 (s, 1H), 8.11 – 8.04 (m, 3H), 8.04 (d, *J* = 8.3 Hz, 1H), 8.00 – 7.94 (m, 3H), 7.69 – 7.55 (m, 5H), 7.55 – 7.50 (m, 2H), 7.47 (d, *J* = 8.2 Hz, 1H), 7.45 – 7.40 (m, 1H), 7.09 – 7.03 (m, 2H), 4.55 – 4.40 (m, 2H), 1.41 (t, *J* = 7.1 Hz, 3H).

^{13}C{^{1}H} NMR (126 MHz, $CDCl_3$) δ 162.5, 152.5, 151.5, 149.4, 137.5, 135.1, 134.5, 134.4, 133.6, 129.9, 129.7, 129.0, 128.8, 128.0, 127.3, 126.1, 125.8, 123.1, 121.2, 121.1, 120.6, 117.0, 92.4, 91.0, 61.3, 14.4.

HRMS (ESI+): m/z calcd for $C_{30}H_{23}N_2O_3$ ($[M+H]^+$) 459.1703; found 459.1701

3-Mesityl-5-morpholinobenzo[d]isoxazole (**13d**)

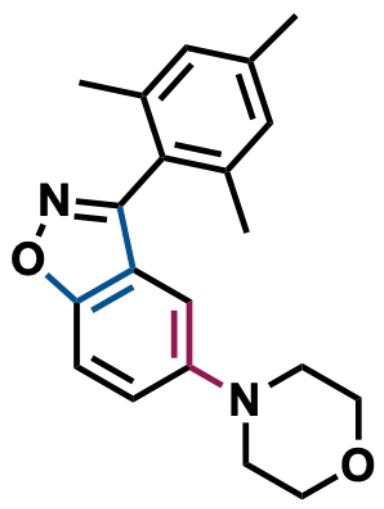

13d

To a round bottom flask (10 mL) were added **11f** (46 mg, 0.10 mmol, 1.0 equiv.), KF (17.4 mg, 0.30 mmol, 3.0 equiv.), and 18-crown-6 (79 mg, 0.30 mmol, 3.0 equiv.). The flask was filled with N_2 and THF (1 mL) and morpholine (9.5 µL, 9.6 mg, 0.11 mmol, 1.1 equiv.) were added. The reaction mixture was stirred at 20 °C for 18 h. The reaction mixture was diluted with brine (10 mL) and extracted with EtOAc (3 x 15 mL). The combined organic phases dried (Na_2SO_4), filtered and concentrated under reduced pressure. The crude product was purified by column chromatography (EtOAc/hexanes 15:85) to afford **13d** (16 mg, 0.050 mmol, 50%) as a yellow oil.

Regioselectivity (**13d**:**13d'**): 6.0:1.0 (based on ratio between the higher shift morpholine signal of **13d** and the only unassigned morpholine peak in the crude ^{1}H NMR spectrum).

^{1}H NMR (400 MHz, $CDCl_3$): δ 7.56 (d, *J* = 9.1 Hz, 1H), 7.29 (dd, *J* = 9.0, 2.4 Hz, 1H), 7.01 (s, 2H), 6.70 (d, *J* = 2.4 Hz, 1H), 3.94 – 3.79 (m, 4H), 3.34 – 3.00 (m, 4H), 2.37 (s, 3H), 2.09 (s, 6H).

^{13}C{^{1}H} NMR (101 MHz, $CDCl_3$): δ 158.9, 158.0, 149.0, 139.4, 137.9, 128.6, 124.6, 122.9, 122.0, 110.6, 106.3, 67.0, 51.1, 21.4, 20.3.

HRMS (EI+): m/z calcd for $C_{20}H_{22}N_2O_2$ ($[M]^+$): 322.1676; found 322.1676

2-(tert-Butyl)-N-(4-iodophenyl)-3-phenyl-2,3-dihydrobenzo[d]isoxazol-5-amine (**13e**)

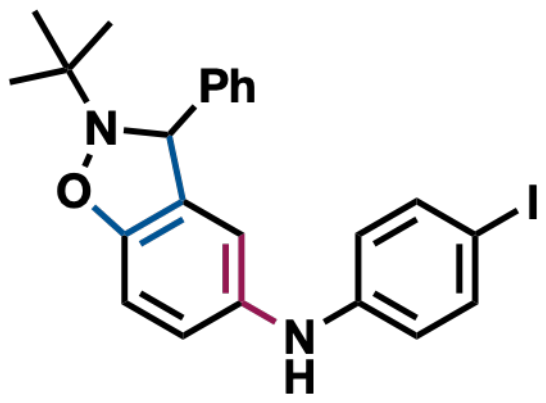

13e

To a 10 mL microwave vial were added **11g** (48 mg, 0.10 mmol, 1.0 equiv.), 4-iodoaniline (33 mg, 0.15 mmol, 1.5 equiv.), CsF (77 mg, 0.50 mmol, 5.0 equiv.), and 18-crown-6 (79 mg, 0.30 mmol, 3.0 equiv.). The vial was sealed under N_2 and MeCN (1 mL) was added. The reaction mixture was stirred at 20 °C for 18 h. The reaction mixture was diluted with brine (10 mL) and extracted with EtOAc (3 x 15 mL). The combined organic phases dried (Na_2SO_4) filtered and concentrated under reduced pressure. The crude product was purified by column chromatography using a 4:1 hexane/EtOAc mobile phase to afford **13e** as a red oil (35 mg, 0.075 mmol, 75%).

The regioselectivity was determined by assuming that the largest unidentified peak in the chemical shift range 5.5-5.7 ppm (corresponding to the benzylic proton) in the crude ^{1}H NMR spectrum belonged to the minor isomer. This gave a lower bound on the regioselectivity of 11:1

^{1}H NMR (400 MHz, $CDCl_3$): δ 7.44 – 7.38 (m, 4H), 7.37 – 7.30 (m, 2H), 7.29 – 7.23 (m, 1H), 6.91 (d, *J* = 8.6 Hz, 1H), 6.74 (d, *J* = 8.5 Hz, 1H), 6.64 (s, 1H), 6.57 – 6.52 (m, 2H), 5.57 (s, 1H), 5.36 (s, 1H), 1.20 (s, 9H).

^{13}C{^{1}H} NMR (101 MHz, $CDCl_3$): δ 152.8, 145.4, 143.7, 138.0, 135.1, 131.3, 128.8, 127.6, 127.4, 123.0, 117.9, 117.2, 107.4, 80.1, 67.3, 61.2, 25.6.

HRMS (ESI+): m/z calcd for $C_{23}H_{24}N_2OI$ ($[M+H]^+$): 471.0928; found 471.0926

1-Benzyl-5-(phenylthio)-1H-benzo[d][1,2,3]triazole (**13f**)

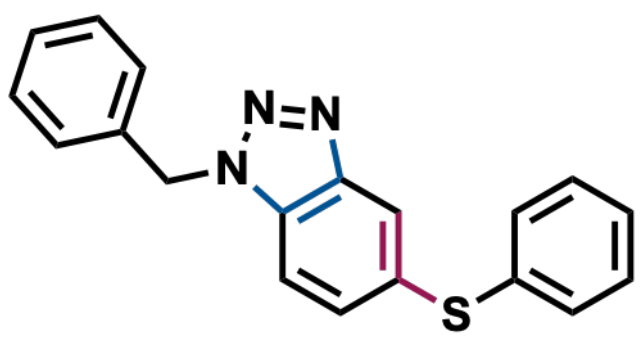

13f

To a round bottom flask (10 mL) were added KF (17.4 mg, 0.30 mmol, 3.0 equiv.) and 18-crown-6 (79 mg, 0.30 mmol, 3.0 equiv.). The flask was filled with N_2 and THF (1 mL), **11h** (43 mg, 0.10 mmol, 1.0 equiv.), and thiophenol (12 mg, 0.15 mmol, 1.5 equiv.) were added. The reaction mixture was stirred at 20 °C for 18 h. The reaction mixture was diluted with brine (10 mL) and extracted with EtOAc (3 x 15 mL). The combined organic phases dried over Na_2SO_4, filtered and

concentrated under reduced pressure. The crude product was purified by column chromatography (EtOAc/hexanes 1:9) to afford **13f** (24 mg, 0.076 mmol, 75%) as a yellow oil.

The regioselectivity was determined by assuming that the largest unidentified peak in the chemical shift range 5.7-5.9 ppm (corresponding to the benzylic protons) in the crude ^{1}H NMR spectrum belonged to the minor isomer. This gave a lower bound on the regioselectivity of ≥1.9:1.0.

^{1}H NMR (400 MHz, C_6D_6): δ 8.19 (dt, *J* = 1.7, 0.8 Hz, 1H), 7.22 (m, 2H), 6.97 – 6.87 (m, 6H), 6.85 – 6.80 (m, 2H), 6.62 (dt, *J* = 8.6, 0.9 Hz, 1H), 5.06 (s, 2H).

^{13}C{^{1}H} NMR (101 MHz, $CDCl_3$): δ 147.2, 135.5, 134.5, 132.2, 132.0, 131.3, 131.0, 129.5, 129.2, 128.8, 127.8, 127.6, 122.4, 110.5, 52.6.

HRMS (ESI+): m/z calcd for $C_{19}H_{16}N_3S$ ([M+H]$^+$) 318.1059; found 318.1057

N-(4-iodophenyl)-3-mesitylbenzo[*d*]isoxazol-5-amine (**13g**)

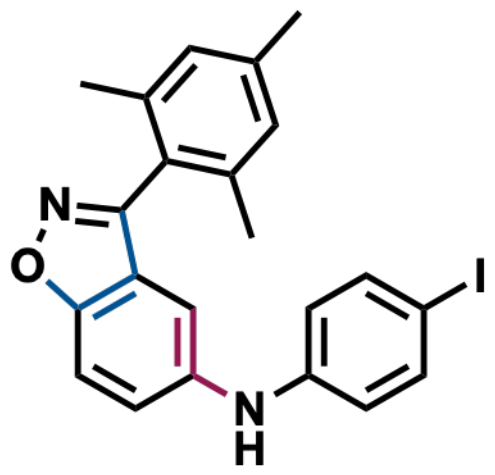

13g

To a 10 mL round bottom flask were added **11f** (45.7 mg, 0.10 mmol, 1.0 equiv.), KF (17.4 mg, 0.30 mmol, 3.0 equiv.), and 18-crown-6 (79.3 mg, 0.30 mmol, 3.0 equiv.). The flask was evacuated and backfilled with N_2 and THF (1 mL) and 4-iodoaniline (32.8 mg, 0.15 mmol, 1.5 equiv.) were added. The reaction mixture was stirred at 20 °C for 18 h. The reaction mixture was diluted with brine (10 mL) and extracted with EtOAc (3 x 15 mL). The combined organic phases dried (Na_2SO_4,), filtered and concentrated under reduced pressure. The crude product was purified by combi flash chromatography, column chromatography and then preparative TLC (EtOAc/hexanes 1:4) to afford **10i** (25 mg, 0.055 mmol, 55%) as a yellow oil. Regioselectivity (**13g**:**13g'**): 8.0:1.0 determination based on aromatic AB-system in the crude ^{1}H NMR spectrum.

^{1}H NMR (500 MHz, $CDCl_3$): δ 7.58 (d, *J* = 8.8 Hz, 1H), 7.48 (d, *J* = 8.6 Hz, 2H), 7.33 (dd, *J* = 8.8, 2.2 Hz, 1H), 7.03 (d, *J* = 2.2 Hz, 1H), 6.98 (s, 2H), 6.71 (d, *J* = 8.7 Hz, 2H), 5.66 (s, 1H), 2.35 (s, 3H), 2.09 (s, 6H).

^{13}C{^{1}H} NMR (126 MHz, $CDCl_3$): δ 159.9, 157.8, 144.0, 139.5, 138.6, 138.3, 137.7, 128.7, 124.5, 124.2, 123.3, 118.5, 111.2, 111.1, 82.0, 21.4, 20.3.

HRMS (ESI+): m/z calcd for $C_{22}H_{20}N_2OI$ ([M+H]$^+$) 455.0615; found 455.0601

1-benzyl-*N*-(4-iodophenyl)-1*H*-benzo[*d*][1,2,3]triazol-5-amine (**13h**)

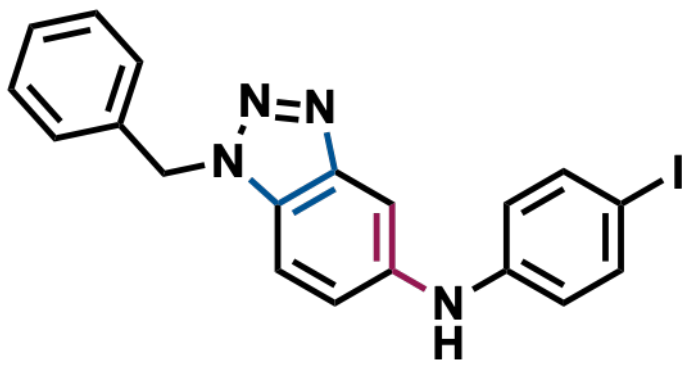

13h

To a round-bottom flask (10 mL) was added **11h** (42.9 mg, 0.10 mmol, 1.0 equiv.), KF (17.4 mg, 0.30 mmol, 3.0 equiv.), and 18-crown-6 (79.3 mg, 0.30 mmol, 3.0 equiv.). The flask was evacuated and backfilled with N_2 and then THF (1 mL) and 4-iodoaniline (32.8 mg, 0.15 mmol, 1.5 equiv.) were added. The reaction mixture was stirred at 20 °C for 20 h, diluted with brine (10 mL) and extracted with EtOAc (3 x 15 mL). The combined organic phases were dried (Na_2SO_4), filtered and concentrated under reduced pressure. The crude product was purified by combi flash chromatography, column chromatography and then with preparative TLC using 50% EtOAc in hexanes as the mobile phase to afford **13h** (19 mg, 0.044 mmol, 44%) as a colourless solid and the only observed regioisomer.

^{1}H NMR (500 MHz, $CDCl_3$): δ 7.68 (s, 1H), 7.51 (d, *J* = 8.1 Hz, 2H), 7.43 – 7.31 (m, 3H), 7.30 – 7.26 (m, 2H), 7.24 (d, *J* = 8.7 Hz, 1H), 7.14 (d, *J* = 8.8 Hz, 1H), 6.81 (d, *J* = 8.2 Hz, 2H), 5.85 (s, 1H), 5.81 (s, 2H).

^{13}C{^{1}H} NMR (126 MHz, $CDCl_3$): δ 147.61, 143.39, 139.17, 138.31, 134.79, 129.38, 129.18, 128.67, 127.73, 122.58, 119.13, 110.70, 107.14, 82.59, 52.58.

HRMS (ESI+): m/z calcd for $C_{19}H_{16}N_4I$ ($[M+H]^+$): 427.0414; found 427.0400.

Computational details

Computational methods

Input structures were prepared in Avogadro (v.1.2.0) [Avogadro: an open-source molecular builder and visualization tool. Version 1.2.0. (http://avogadro.cc/),[15] and performing a preliminary geometry optimisation using the Universal Force Field[16] optimiser. The geometries were calculated using ORCA (v. 6.1.0)[17] using the composite method PBEh-3c[18] while applying an acetonitrile SMD solvent model.[19] The bond angle difference ("BAD" or Δθ) was calculated as the difference between the internal bond angles of the two carbon atoms in the aryne triple bond.

3-OTf-benzyne and 4-OTf-benzyne intermediates

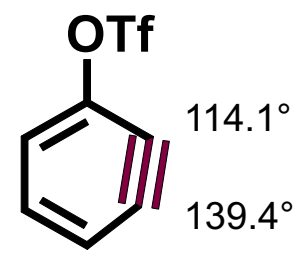

3-OTf-benzyne
Δθ = 25.3°

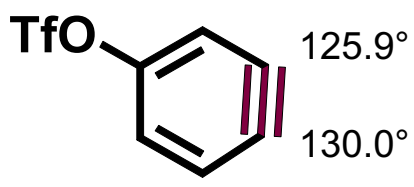

4-OTf-benzyne
Δθ = 4.1°

Additional aryne geometries

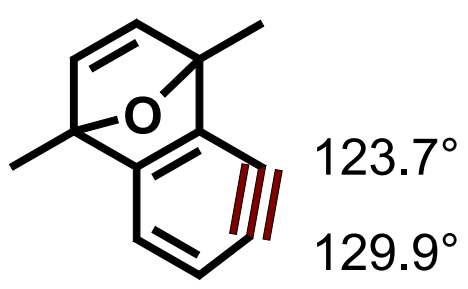

12h
Δθ = 6.2°

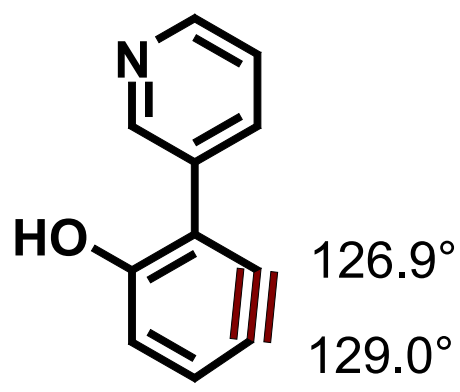

12i
Δθ = 2.1°

.xyz files

9a

29

Coordinates from ORCA-job Output/1367/1367 E -1597.729204154941

C	-0.52278027757589	1.06685494622277	0.42652334888182
C	-1.25899163838285	2.18421719117576	0.84714055882260
C	-0.68649328254718	3.35245721742813	1.39302173444586
C	0.65063182614048	3.14209320843313	1.40966267879164
C	1.45626672922294	2.24484544678143	1.08960873427561

C	0.87175521859406	1.11621069308326	0.56081638878494
O	1.58148523095663	-0.00238184874749	0.14367474374384
S	3.18712045862683	0.01731424140515	0.23714995789238
O	3.73402176239218	0.94911912760548	-0.71014923835314
O	3.61175464155169	-0.01397613487463	1.60965325451978
C	3.39714691584656	-1.68375344781127	-0.45429492483183
F	4.69456717149680	-1.92163364937066	-0.48503415417041
F	2.79909563672269	-2.56414263390273	0.32127421758920
F	2.90263094614390	-1.74478321353920	-1.67321952808574
Si	-1.41533141413861	-0.45104241643598	-0.30359904285303
C	-1.06870106175377	-1.95876835171045	0.76322668742300
C	-0.85007773003172	-0.71285915378631	-2.07638804086603
C	-3.26041748145965	-0.09876023072082	-0.29000623458503
H	-2.33884968920632	2.17694474666501	0.75817675807956
H	-1.25595127566142	4.20971856799138	1.71597481102087
H	-0.02203149851538	-2.26280564489701	0.74837315107649
H	-1.65793758434543	-2.80954670869133	0.41267421212839
H	-1.34665514558458	-1.77822666842451	1.80362381588230
H	0.20470448659843	-0.97669327379499	-2.15421442617545
H	-1.01251292421587	0.18341720150998	-2.67857303252196
H	-1.42109322382977	-1.52210818400838	-2.53789062126304
H	-3.52350575860364	0.76981641635639	-0.89659329912049
H	-3.64565011505830	0.06625703313580	0.71800162078804
H	-3.79894192338280	-0.95474647707793	-0.70385213132016

9b

30

Coordinates from ORCA-job Output/1959/1959 E -876.141002873112

C	-0.12396598425762	-2.40912415146548	0.52177828507763
C	2.76451471965471	-0.08859420150951	-0.26471453029448
C	3.52709077369728	-1.09083807655651	0.35198757162993

C	4.43938302024416	-0.77805367918232	1.36912714806020
C	4.40209075346819	0.57411758841268	1.59749916831878
C	3.72703354711435	1.45836519664357	1.05082582197219
C	2.81521876693301	1.27908422974782	0.03872995711740
I	1.45010421266709	-0.70174023505578	-1.79916179367385
C	-0.37759677373799	-0.25844460545208	-0.83091028834826
C	-1.04752379275870	0.90724779593816	-1.20932853509404
C	-2.25475185094687	1.15778834048184	-0.56781029128485
C	-2.77406564574757	0.30113463596338	0.39828985952255
C	-2.05993124458567	-0.84303518462823	0.72810707432376
C	-0.84611006756340	-1.15989052426023	0.12249441804844
C	-4.07849397434065	0.61701450984401	1.06234512502957
C	-0.54526030207914	1.87075165263527	-2.24174230140109
H	0.85091155211643	-2.18901084477419	0.96002345798015
H	0.04007319131970	-3.07269384630495	-0.32847463662415
H	-0.70039184825362	-2.95866165635110	1.26349401117342
H	3.41599286383450	-2.12331147610040	0.04619888924786
H	5.04532346808975	-1.52340874450375	1.86249403333052
H	2.22038006078196	2.03007438161083	-0.46164214345342
H	-2.80418373414718	2.05319758971885	-0.83510498202381
H	-2.45305255614614	-1.51915590405557	1.47803313700773
H	-4.02509944832137	1.56640005404927	1.59751715035363
H	-4.36398793613972	-0.15460992306153	1.77541217793305
H	-4.87963161042844	0.71053346974486	0.32772007357921
H	0.43004563111544	2.27979092459679	-1.97424420073612
H	-0.44662957402143	1.40109913702902	-3.22141877699390
H	-1.23551721756104	2.70565354684528	-2.34532287977805

9a

28

Coordinates from ORCA-job FBA185/FBA185 E -669.414805733599

C	-5.03007867089651	5.30977116697363	0.57682142288044

C	-4.89956402938209	3.98601624149940	0.95794430987488
C	-3.65150592083518	3.47412860594080	1.39687312114268
C	-2.70670593937552	4.46286911259777	1.36538912972623
C	-2.80020650331649	5.66178644423021	1.03394159338456
C	-3.96113214022982	6.22804795780173	0.59434008572697
H	-5.74988422802902	3.31536190858779	0.92592205859973
H	-3.52376783599596	2.44436847020861	1.69718848888428
C	-6.21831817782061	6.14110101746985	0.10979259137797
C	-6.51163344805572	7.13199972151096	1.24881291149242
C	-5.49420926081791	7.98507582167486	1.26874167376371
C	-4.54298686880683	7.55043510648947	0.14167745311783
N	-5.50467150859755	7.00759825173402	-0.84737673820459
H	-7.34855190794085	7.06298098532991	1.92810735215060
H	-5.29557688950407	8.78633843495112	1.96518998413610
H	-3.84200629561374	8.28768349671070	-0.23611908570641
H	-7.05411091339391	5.57994815094057	-0.29804387300210
C	-6.22478879323717	7.91085949277460	-1.63460304897482
C	-5.53126802102743	8.89673316347937	-2.34720992894968
C	-6.20595185331213	9.77699748257382	-3.17571393885809
C	-7.58548016627884	9.69480350548763	-3.32837730443173
C	-8.27666576704501	8.71631640375122	-2.63117306902741
C	-7.61132946421975	7.83730394630116	-1.78531472980321
H	-4.45413943865631	8.96911442570792	-2.26394378343109
H	-5.64417243531676	10.53051726576093	-3.71332562146351
H	-8.10911014079526	10.38136802680958	-3.98040359694186
H	-9.35179766991419	8.63523462435345	-2.73127699683450
H	-8.18862571158530	7.09515076834883	-1.25055046062939

12b

17

Coordinates from ORCA-job FBA180/FBA180 E -458.692769477220

C	-4.83529783261235	4.05518807403398	-0.24464904691958
C	-4.73893853851988	2.71854291585762	0.09444850023791
C	-3.50046985396596	2.15781441356771	0.50357142732309
C	-2.52164010451126	3.11504077370419	0.49802880418737
C	-2.60145358611406	4.32605065827725	0.20884125338736
C	-3.74042652628199	4.94459349589605	-0.20708546634659
H	-5.60786410705777	2.07286873889563	0.04945797271235
H	-3.40114124812460	1.11417446600993	0.76468837868318
C	-5.98505419905655	4.96203099277864	-0.68632965617049
C	-6.26270436487990	5.90559119920111	0.48762111104388
C	-5.22510872138604	6.73162454069082	0.52714048684678
C	-4.31695303397690	6.28994267591901	-0.62483325300097
H	-7.10241173641303	5.82708523811228	1.16177541205145
H	-4.98995263106348	7.50596925052011	1.24168969491718
O	-5.28369726896810	5.83710131986004	-1.56752416558787
H	-6.83825178815614	4.48637664049941	-1.16466111793256
H	-3.63094445891193	7.02298460617615	-1.04217033543250

12c

17

Coordinates from ORCA-job FBA189a/FBA189a E -3031.108099218831

C	-4.96962610484852	4.08223891981408	-0.23182893134890
C	-4.87129342846585	2.72700362200594	0.02086478321329
C	-3.67149309124069	2.17133465360881	0.53738768065306
C	-2.74205005674478	3.15790389080745	0.71366286355467
C	-2.80947299439319	4.38671231949599	0.50877487743161
C	-3.91931453334275	4.99354183978167	0.00078178163141
H	-5.70514694826935	2.06381235044069	-0.17532098190547
H	-3.56450616147265	1.11515578792748	0.73653965958606
C	-6.08601453945116	4.98619174378713	-0.75092164026807
C	-6.51872462826144	5.84291859145373	0.44759264600578

C	-5.53016679924499	6.69295664768878	0.68259059614468
C	-4.48781256943246	6.35147748133309	-0.38681832436913
H	-5.40004319908517	7.40674904735126	1.48128820720780
O	-5.32697816046768	5.94865102695565	-1.46849065494657
H	-3.78626736493721	7.13324527999392	-0.66578651604791
H	-6.86793948591100	4.53083455788244	-1.35492912725750
Br	-8.06482993443107	5.53149223967183	1.42008308071519

12c'

17

Coordinates from ORCA-job FBA189b/FBA189b E -3031.107798034936

C	-5.00135332098138	4.04621321177336	-0.23535234483037
C	-4.87430435889638	2.69529296031862	0.02827603727595
C	-3.65938424458994	2.16629329970373	0.53753273482434
C	-2.74712413262282	3.17106245260142	0.69721628099936
C	-2.83632111121819	4.39682134217351	0.48251983065562
C	-3.96417275102365	4.97580985974248	-0.01835186852441
H	-5.69734380850120	2.01452214289122	-0.15262725397417
H	-3.53103165093332	1.11395534191031	0.74437128303518
C	-6.14246360717983	4.92589319915810	-0.74528986411012
C	-6.60995343808576	5.76299287253486	0.44914174682462
C	-5.62318597091570	6.62490826936341	0.64504747223981
C	-4.55924720345406	6.31783913068244	-0.41896557229031
Br	-5.38234304273190	7.85741058729970	2.00759954507769
O	-5.39821142551214	5.89794949259237	-1.48265140632715
H	-3.87655943381886	7.11583335723595	-0.70174115111203
H	-6.91300228927181	4.44986877498110	-1.34672807168743
H	-7.50566821026300	5.58950370503737	1.02545260192344

12d

43

Coordinates from ORCA-job FBA188/FBA188 E -1073.134603599709

C	-4.74848258097693	4.50567378848232	0.82302115815058
C	-4.35352809470087	3.42624100277833	1.59640860190169
C	-3.02436933609099	3.32465133218632	2.07477883406866
C	-2.30494590003787	4.40850039040069	1.65718210927936
C	-2.61710538161983	5.40577739525945	0.97791095097075
C	-3.87828849438736	5.55257760989891	0.47160699473334
H	-5.04659493026225	2.63169367648609	1.84374476205681
H	-2.68479830430867	2.48934079740770	2.66902330440168
C	-6.07774565824903	4.91710129219562	0.17365181305207
C	-6.56586759366662	6.13157757453762	0.97160766401196
C	-5.68651734529942	7.15658367206671	0.63034314954487
C	-4.70906957961432	6.52074979111100	-0.35890464990132
C	-5.80403086597565	8.41608459432034	1.16549362286486
O	-5.58111048389620	5.58362969478073	-0.98797343245735
C	-6.85206615154876	8.64241238020213	2.06449894761808
C	-7.73044991778541	7.62525320764065	2.40305010383652
C	-7.59464913031581	6.34330169214598	1.85804095553817
H	-8.53754715540342	7.82462705319521	3.09604856385852
H	-8.28987434724917	5.55894747890707	2.12851854390264
H	-5.12213122593399	9.21462031784988	0.90076081278137
H	-6.98066368933672	9.62644080093674	2.49638040373991
C	-4.00050458847364	7.41770418353729	-1.31755468871862
C	-2.96327574953420	8.22319568194017	-0.85296232218622
C	-2.30204694360757	9.08206783910304	-1.71891919312651
C	-2.66986012009040	9.14269141039126	-3.05650614189449
C	-3.70377268509464	8.34195458601191	-3.52101520075374
C	-4.37029101812595	7.48312523486891	-2.65679484588366
H	-5.17571334529794	6.86851421536239	-3.03575830357054
H	-2.66831979979208	8.18686315265297	0.18898170566886
H	-1.49764413014167	9.70310653211243	-1.34659529390951

H	-2.15315595952510	9.81107683540474	-3.73305554613115
H	-3.99634588981872	8.38370275759351	-4.56227522271226
C	-7.06669080615525	3.84570332074274	-0.15243639870966
C	-7.41975740552509	3.56287337640783	-1.46791954564559
C	-8.34526313961934	2.56399825696106	-1.74239413683019
C	-8.92616766179170	1.84109183407212	-0.71064257628506
C	-8.57567291159028	2.12052209770052	0.60377891852875
C	-7.65146586267898	3.11659104305408	0.88136695156621
H	-6.97934734641858	4.11472930856241	-2.28699814127585
H	-8.61170456170733	2.35280016029797	-2.77003569497644
H	-9.64717059069205	1.06378379183752	-0.92834922672907
H	-9.02150092688850	1.56251644353035	1.41691835403481
H	-7.38538239077145	3.32231239506500	1.91122333558675

12e

31

Coordinates from ORCA-job FBA119/FBA119 E -745.726259747482

C	-4.10195362402735	6.44725434589261	0.39909307028892
C	-4.95479403295092	5.51871871323915	1.01975471944636
C	-5.06420554498687	5.52968855416048	2.40860401977768
C	-4.25728988586276	6.52002660932547	2.93651003476916
C	-3.49127689702970	7.36326927009342	2.45410710654621
C	-3.33363723852184	7.40398263125834	1.07296724818912
H	-5.52110495282328	4.80276949511676	0.43846675393445
C	-2.65448093723690	8.09728692886012	0.02619235242495
N	-2.99218961622112	7.59596001114088	-1.12250400344851
O	-3.88600651913542	6.57679416315155	-0.91328868717080
C	-1.71979165451501	9.23095711052083	0.12889973132990
C	-2.09627694996414	10.48997678385435	-0.35076920692639
C	-1.19673072356532	11.54505982739528	-0.22586400644294
C	0.05410689891411	11.37736312697140	0.35558292846183

C	0.40310060105416	10.11229084664805	0.81956199530856
C	-0.46527790107068	9.03270403777502	0.72075053687906
H	1.37904990703209	9.96002030303212	1.26770672583887
H	-1.48451624124262	12.52399089507964	-0.59302122149625
C	-3.43593977894423	10.72692889876120	-0.98651244490037
H	-4.25159971541792	10.32805375828437	-0.38154559698328
H	-3.50655914948466	10.25602298239311	-1.96784504607784
H	-3.61442847769736	11.79292147591701	-1.12108329551224
C	-0.04587277767769	7.68558767783014	1.23372741572984
H	1.01481420894667	7.67913700823967	1.48132137946348
H	-0.59144592894338	7.41100448523573	2.13871376536684
H	-0.21972493842050	6.89534999873560	0.50195291034330
C	1.00260433202705	12.52954046331895	0.50035441404670
H	0.69813380303665	13.38031806909965	-0.10814047283061
H	2.01624713548151	12.25130846653855	0.20975496704446
H	1.05104796935342	12.86781791523057	1.53758292113978
H	-5.69945137010597	4.84337514689989	2.94801898545976

12f

36

Coordinates from ORCA-job FBA094/FBA094 E -786.127889976706

C	-4.28653303392998	7.81771479560416	-0.33210540485044
C	-4.46765666882286	7.24707216540223	0.93574782239717
C	-4.43959893357613	5.85240970348386	1.08700712496157
C	-4.22106631650864	5.25868159627761	-0.13100168521435
C	-4.05144132437880	5.72453773570136	-1.27075183676992
C	-4.06276257370108	7.08147590748332	-1.49267607511519
H	-4.57324601233989	5.37009988065662	2.04405815523333
H	-4.63031998478210	7.87396937388031	1.80346025073291
C	-3.90694240315242	8.02809423468853	-2.63815686150497
N	-4.00244854380990	9.34478004697420	-1.96910454346401

O	-4.29265304973255	9.13454994892259	-0.57013087902111
C	-5.01680033892977	10.27401354100054	-2.50328106779525
C	-4.91609779740592	11.58540421547372	-1.72504742767877
H	-5.55868773759373	12.33524404360276	-2.18819556267726
H	-3.89438531839862	11.96909717012121	-1.73428033548836
H	-5.23397290212999	11.48169822586315	-0.68785419545525
C	-4.62850152600194	10.54666172954226	-3.95667748401465
C	-6.44531990604165	9.73198499537646	-2.42789170047388
H	-5.28524138103437	11.31576163176754	-4.36431748833809
H	-4.72858965591470	9.66877432229513	-4.59662654852809
H	-3.60212317513325	10.90926220487928	-4.03217925905993
H	-7.13839312194061	10.46172690715859	-2.84913710068712
H	-6.75700530213774	9.54465074339027	-1.39968090831559
H	-6.57201606522723	8.80849319046487	-2.99615500786948
C	-2.59429210783343	7.85937072401415	-3.36782221625224
H	-4.72248480294145	7.87127158638861	-3.35448222469805
C	-1.40313397823843	8.34323202086133	-2.83512647523779
C	-0.20349458185036	8.13312424662496	-3.49959807305532
C	-0.18064167811846	7.43763456649768	-4.70288200571427
C	-1.36596656830975	6.95382154180277	-5.23825727416472
C	-2.56679787569806	7.16661435433975	-4.57267532256858
H	-1.41165405488282	8.89100468816757	-1.90118521330294
H	0.71758033051465	8.51539823134878	-3.07810707291976
H	0.75632665125195	7.27755540345956	-5.22081442652448
H	-1.35907013284994	6.41561558553140	-6.17752194457005
H	-3.49210812841994	6.79529874095270	-4.99841973199501

12g

25

Coordinates from ORCA-job FBA097/FBA097 E -663.444492595898

C	-5.04186728681297	7.14198801572668	0.40084269329919

C	-5.52260933329860	6.65945271565889	1.63563433665918
C	-4.78103108103984	5.70486058633657	2.32051983112727
C	-3.63811920573787	5.39953511838982	1.60039088965651
C	-3.13524743696835	5.75699204161204	0.52879963538923
C	-3.83813148356457	6.71654920895568	-0.20535252465289
H	-5.08962480067986	5.29277995313026	3.26912351879777
H	-6.45359656741109	7.01715093369852	2.05593698214058
N	-5.51364240866563	8.05068194667180	-0.48123692408090
N	-4.69153516654341	8.16118908588621	-1.51739822389962
N	-3.68595984207062	7.37979489484756	-1.37460242540089
C	-6.75333125417196	8.80114179359737	-0.43993571981463
C	-7.94929761350891	7.92639216548139	-0.70951436897430
H	-6.66124948789101	9.58863768088535	-1.18756222926405
H	-6.83419960101665	9.27848060186039	0.53653506973973
C	-8.94751177625885	7.78615231655693	0.24695716347337
C	-10.05489224533508	6.98443049995664	-0.00338979554819
C	-10.16688971604082	6.31314574668710	-1.21206500085590
C	-9.16935255789247	6.44729313741577	-2.17134954801747
C	-8.06746659802629	7.25092640338317	-1.92224037663462
H	-7.29664206785065	7.35347332788471	-2.67765425095129
H	-11.02743336341771	5.68653512263141	-1.40825403855986
H	-9.25270317781737	5.92716096599819	-3.11694281295418
H	-8.86067914912866	8.30682799973176	1.19342379477675
H	-10.82653677885057	6.88299773701552	0.74890432454921

12h

23

Coordinates from ORCA-job FBA058/FBA058 E -537.171899448500

C	-4.99670831542303	4.08687677258296	-0.15042756802573
C	-4.86179229321440	2.72719917631265	0.05622093336160
C	-3.59947852570954	2.15957231654842	0.37707743482365

C	-2.64684905064670	3.13898534770270	0.43885735740528
C	-2.75223858018277	4.37120070316973	0.26791240475739
C	-3.92168336871166	4.99209607818179	-0.05461589940303
H	-5.71545828787210	2.06482738534327	-0.02824863074589
H	-3.46976456946839	1.09859545365147	0.53382261102260
C	-6.18378328472264	4.99775199790593	-0.49308875692026
C	-6.43938085429695	5.82971155571806	0.77293693474573
C	-5.41842104313468	6.66967396343427	0.86583433668674
C	-4.51994212623639	6.36517387389713	-0.34261096459041
H	-7.26262661542698	5.67231373192299	1.45516755870185
H	-5.18193895755286	7.38129681622411	1.64387899113048
O	-5.50626370975592	5.96785191956190	-1.29592285774996
C	-7.36838121133734	4.38419881093619	-1.18198361510802
H	-7.84710777522227	3.65890534011173	-0.52421647316970
H	-7.07384662843711	3.86815376188812	-2.09589568391869
H	-8.10813882428525	5.14420190970733	-1.43485321089360
C	-3.60339055335918	7.44547123956889	-0.83722694770251
H	-2.86972372301363	7.68791929618370	-0.06855505774557
H	-4.15948944835780	8.35399076593155	-1.06878335973931
H	-3.06407225363233	7.13001178351506	-1.73045953692264

12i

20

Coordinates from ORCA-job FBA127/FBA127 E -552.015445275590

C	-4.40551415496274	7.27421035038818	0.34418564675424
C	-4.92298420238694	6.01932023451848	0.73905218167259
C	-4.11271380065172	4.94331274655779	1.09906773561054
C	-2.79108128509674	5.32221384770524	1.01711285152511
C	-2.31401038989313	6.41078930712736	0.67567356753287
C	-3.01227533254165	7.53129287896522	0.26456014705789
H	-4.50896014944390	3.98179789592751	1.39171968083050

C	-2.42111611021619	8.80724356787243	-0.16348043651710
H	-5.99980648473336	5.90002803700446	0.76396883588631
O	-5.32426088105650	8.21404973590485	0.05192434219514
C	-2.83185029223081	9.49095164285589	-1.30626292559466
C	-2.19783555236428	10.67504753135806	-1.64670920254950
C	-1.16947004929894	11.13471751290286	-0.84044521105708
N	-0.75756049774420	10.48764512610151	0.24476469174207
C	-1.36850535407259	9.36057628397145	0.56845391988734
H	-2.48903931507129	11.22716037983114	-2.52960823509809
H	-0.65335820578870	12.05684038612922	-1.08241911708189
H	-3.61161321549109	9.09549011856808	-1.94595534937783
H	-1.01418841766516	8.85293974509829	1.46046262095191
H	-4.92841630929003	9.09497267121187	0.01220425562966

3-OTf-benzyne

17

Coordinates from ORCA-job Output/717/717 E -1189.658710070713

C	-1.31015354346795	0.23194799861951	1.14973362634877
C	-2.70291215667095	0.29076182494121	1.07185500940422
C	-3.40578614082280	0.12775427524814	-0.13700356631714
C	-2.46235272742173	-0.07852895780339	-1.08789399698503
C	-1.22160728649548	-0.16087497071325	-1.18888335629798
C	-0.54839057290506	0.00146812849716	0.00224750618775
O	0.82606001345877	-0.05017536369824	0.15374661853535
S	1.74414541292774	-0.23765484218807	-1.15439723546362
O	1.66113218360966	0.92311400495238	-1.99740066296162
O	1.60904638834450	-1.56981488326200	-1.67567609921875
C	3.33465567192615	-0.15030880046361	-0.21761644601480
F	3.39200151040994	-1.12546352961593	0.66476264417354
F	3.43725932839843	1.01435620320868	0.38726590346483
F	4.30565262234906	-0.28547132051720	-1.10066864320920
H	-0.82511612376672	0.36643195252146	2.10820261138983

H	-3.27244986737038	0.47001993687908	1.97541598544545
H	-4.47959871250316	0.16897134339406	-0.22877189848160

4-OTf-benzyne

17

Coordinates from ORCA-job Output/775/775 E -1189.654704898375

C	0.76048424001417	0.17788199143734	-0.55114545049909
C	1.11195221752244	1.00738681901794	0.52529182502416
C	2.38148466904956	0.94880054248746	1.10583747833323
C	3.12400564318814	-0.01146245333785	0.46183231784881
C	2.82450415492360	-0.74143614883599	-0.49203012746502
C	1.61381183931668	-0.75986930326246	-1.14469011506532
O	-0.50527193734216	0.36579604902351	-1.10599527550707
S	-1.68564491088535	-0.69635674973777	-0.85757278323395
O	-1.15518905821781	-1.99469588337190	-0.54685093620018
O	-2.63639345083260	-0.45829531066699	-1.90387970922885
C	-2.42728436950727	-0.02384923308131	0.69805830369466
F	-3.56119466725456	-0.66471785142760	0.90862410733823
F	-1.60692214063844	-0.22805433866022	1.71029658177096
F	-2.66133406720604	1.26558307692683	0.56148236655003
H	0.38950445322670	1.72121661227390	0.90164444336304
H	2.68206151529568	1.58032287431063	1.92869454934379
H	1.33206686934727	-1.36573969309550	-1.99511757606746

Crystallography data

Table S4: Crystal data and structure refinement for 8.

CCDC #	2535225
Empirical formula	$C_{26}H_{30}F_3IO_6S_2Si$
Formula weight	714.61
Temperature/K	100.00(10)
Crystal system	triclinic
Space group	P-1
a/Å	9.2507(3)
b/Å	11.9952(5)
c/Å	14.6990(5)
α/°	108.583(3)
β/°	94.391(3)
γ/°	95.367(3)
Volume/Å^3	1529.44(10)
Z	2
ρ_{calc}g/cm^3	1.552
μ/mm^{-1}	10.368
F(000)	720.0
Crystal size/mm^3	0.588 × 0.049 × 0.038
Radiation	Cu K_α (λ = 1.54184 Å)
2Θ range for data collection/°	6.384 to 159.858
Index ranges	-11 ≤ h ≤ 11, -15 ≤ k ≤ 14, -17 ≤ l ≤ 18
Reflections collected	24913
Independent reflections	6168 [R_{int} = 0.0923, R_{sigma} = 0.0541]
Data/restraints/parameters	6168/0/359
Goodness-of-fit on F^2	1.139
Final R indexes [I>=2σ (I)]	R_1 = 0.0571, wR_2 = 0.1611
Final R indexes [all data]	R_1 = 0.0597, wR_2 = 0.1654
Largest diff. peak/hole / e Å^{-3}	2.21/-2.89

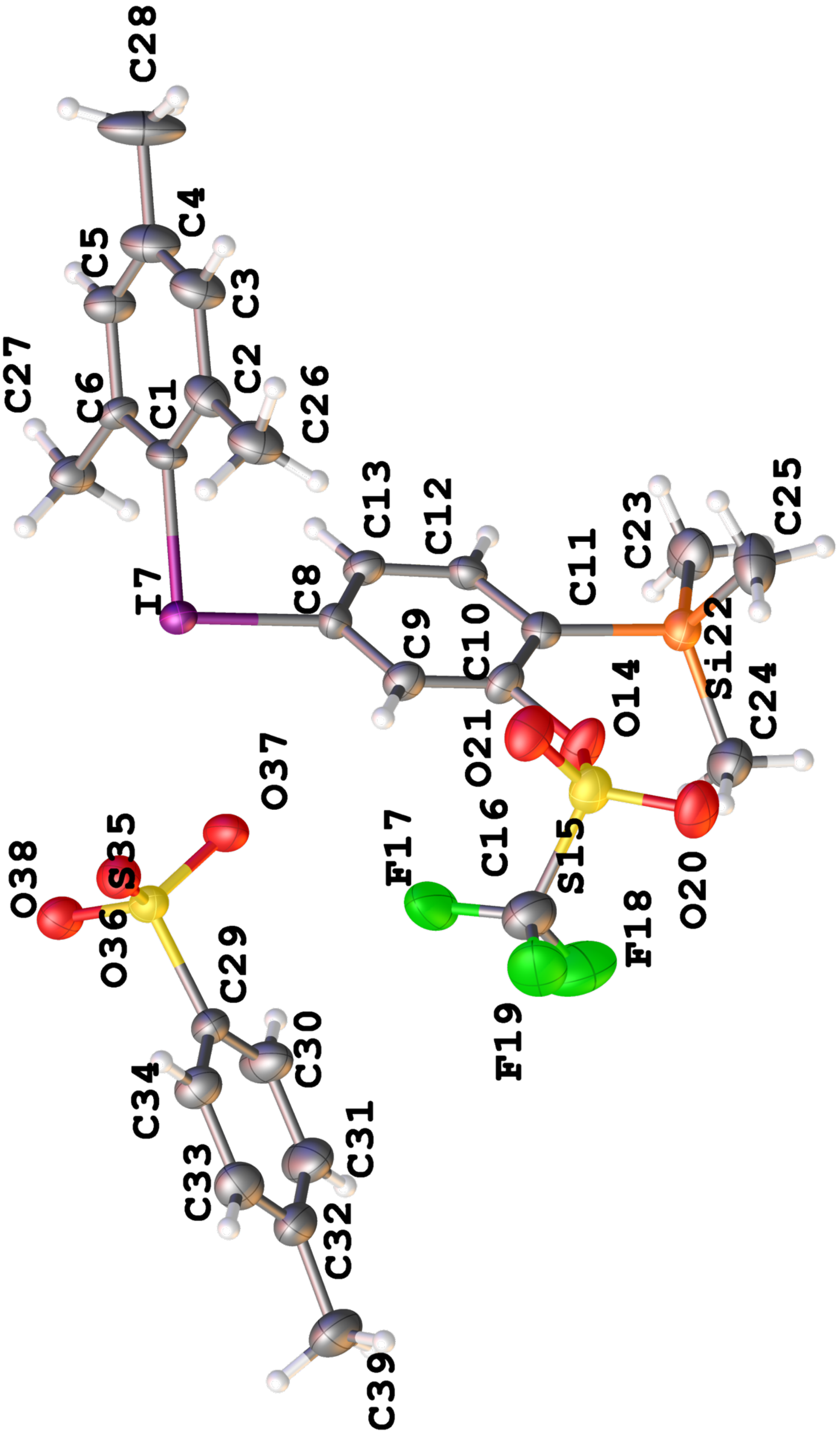

Figure S3: X-Ray crystal structure of **8**. Thermal ellipsoids are plotted at the 50% probability level.

Table S5 Crystal data and structure refinement for AO(11f).

CCDC#	2535224
Empirical formula	$C_{20}H_{22}F_3NO_4SSi$
Formula weight	457.53
Temperature/K	99.98(15)
Crystal system	monoclinic
Space group	$P2_1/c$
a/Å	10.23820(10)
b/Å	10.05530(10)
c/Å	21.3543(3)
α/°	90
β/°	102.2690(10)
γ/°	90
Volume/Å^3	2148.18(4)
Z	4
ρ_{calc}g/cm^3	1.415
μ/mm^{-1}	2.353
F(000)	952.0
Crystal size/mm^3	0.187 × 0.111 × 0.055
Radiation	Cu K_α (λ = 1.54184 Å)
2Θ range for data collection/°	8.474 to 158.448
Index ranges	-13 ≤ h ≤ 13, -12 ≤ k ≤ 11, -27 ≤ l ≤ 27
Reflections collected	21222
Independent reflections	4463 [R_{int} = 0.0242, R_{sigma} = 0.0170]
Data/restraints/parameters	4463/0/278
Goodness-of-fit on F^2	1.045
Final R indexes [I>=2σ (I)]	R_1 = 0.0291, wR_2 = 0.0755
Final R indexes [all data]	R_1 = 0.0303, wR_2 = 0.0763
Largest diff. peak/hole / e Å^{-3}	0.41/-0.41

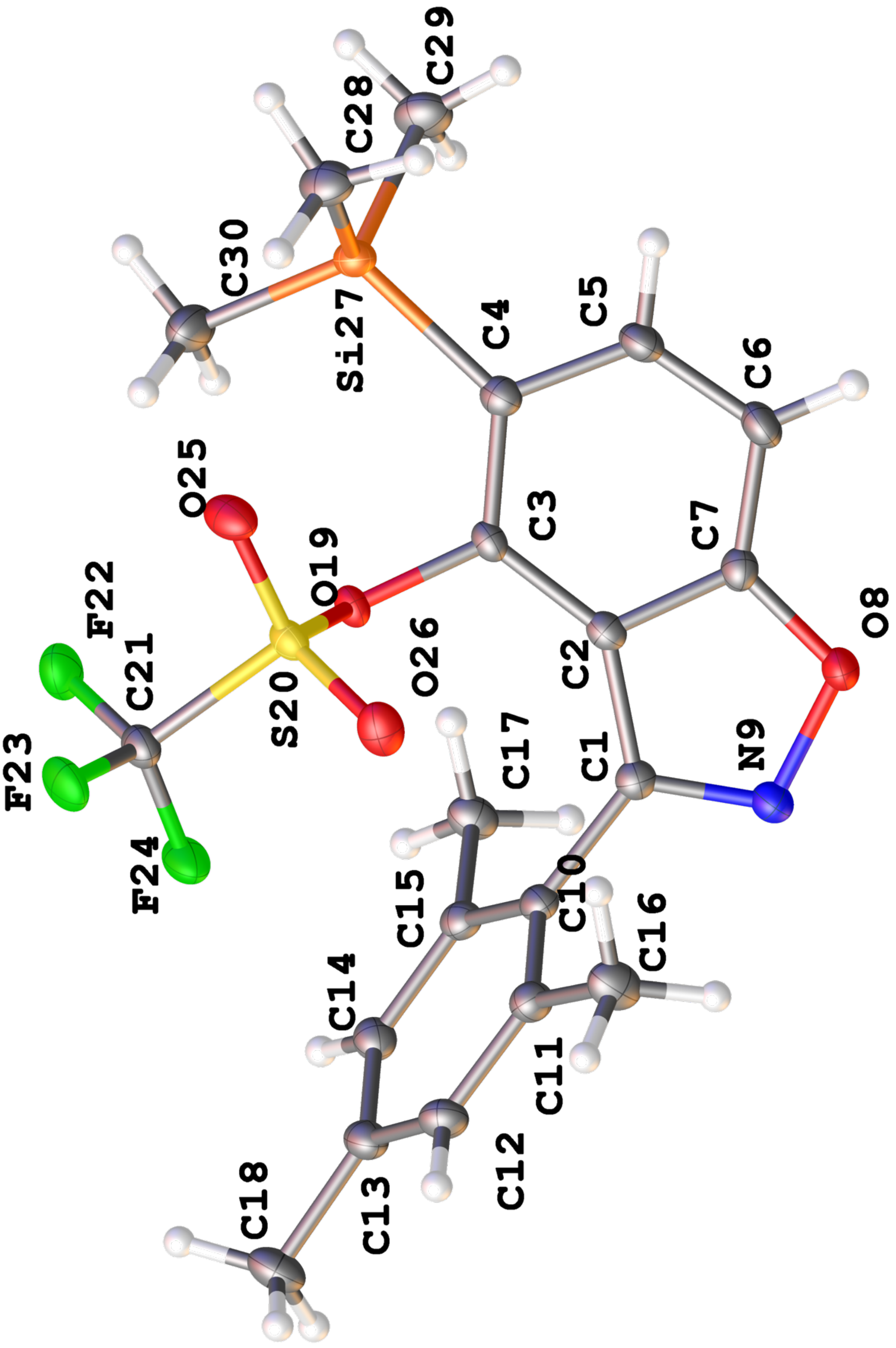

Figure S4: X-Ray crystal structure of **11f**. Thermal ellipsoids are plotted at the 50% probability level.

References

(1) Fulmer, G. R.; Miller, A. J. M.; Sherden, N. H.; Gottlieb, H. E.; Nudelman, A.; Stoltz, B. M.; Bercaw, J. E.; Goldberg, K. I. NMR Chemical Shifts of Trace Impurities: Common Laboratory Solvents, Organics, and Gases in Deuterated Solvents Relevant to the Organometallic Chemist. *Organometallics* **2010**, *29*, 2176-2179

(2) (a) Barnå, F.; Hribersek, M.; Orthaber, A.; Pilarski, L. T. Synthesis and crystal structures of boryl ortho-silylaryl trifluoromethanesulfonates. *Acta Cryst. E* **2024**, *80*, 143-147. (b) Demory, E.; Devaraj, K.; Orthaber, A.; Gates, P. J.; Pilarski, L. T. Boryl (Hetero)aryne Precursors as Versatile Arylation Reagents: Synthesis through C–H Activation and Orthogonal Reactivity. *Angew. Chem. Int. Ed.* **2015**, *54*, 11765-11769

(3) Ye, C.; Twamley, B.; Shreeve, J. n. M. Straightforward Syntheses of Hypervalent Iodine(III) Reagents Mediated by Selectfluor. *Org. Lett.* **2005**, *7*, 3961-3964

(4) Matoušek, V.; Pietrasiak, E.; Sigrist, L.; Czarniecki, B.; Togni, A. O-Trifluoromethylation of N,N-Disubstituted Hydroxylamines with Hypervalent Iodine Reagents. *Eur. J. Org. Chem.* **2014**, *2014*, 3087-3092

(5) Murray, R. W.; Iyanar, K.; Chen, J.; Wearing, J. T. Synthesis of Nitrones Using the Methyltrioxorhenium/Hydrogen Peroxide System. *J. Org. Chem.* **1996**, *61*, 8099-8102

(6) McIntosh, M. L.; Naffziger, M. R.; Ashburn, B. O.; Zakharov, L. N.; Carter, R. G. Highly regioselective nitrile oxide dipolar cycloadditions with ortho-nitrophenyl alkynes. *Org. Biomol. Chem.* **2012**, *10*, 9204-9213

(7) Sakai, T.; Doi, H.; Tomioka, K. Chiral ligand-controlled asymmetric conjugate amination of enoates with lithium mesitylmethyl(trimethylsilyl)amide. *Tetrahedron* **2006**, *62*, 8351-8359

(8) Altintas, O.; Glassner, M.; Rodriguez-Emmenegger, C.; Welle, A.; Trouillet, V.; Barner-Kowollik, C. Macromolecular Surface Design: Photopatterning of Functional Stable Nitrile Oxides. *Angew. Chem. Int. Ed.* **2015**, *54*, 5777-5783

(9) Thoman, C. J.; Voaden, D. J. 3-Phenylsydnone. *Organic Syntheses* **1965**, *45*,

(10) Chen, Y.; Renson, S.; Monbaliu, J.-C. M. On Demand Flow Platform for the Generation of Anhydrous Dinitrogen Trioxide and Its Further Use in N-Nitrosative Reactions. *Angew. Chem. Int. Ed.* **2022**, *61*, e202210146

(11) Fractional factorial designs. NIST. https://www.itl.nist.gov/div898/handbook/pri/section3/pri334.htm.

(12) Nilova, A.; Sibbald, P. A.; Valente, E. J.; González-Montiel, G. A.; Richardson, H. C.; Brown, K. S.; Cheong, P. H.-Y.; Stuart, D. R. Regioselective Synthesis of 1,2,3,4-Tetrasubstituted Arenes by Vicinal Functionalization of Arynes Derived from Aryl(Mes)iodonium Salts. *Chem. Eur. J.* **2021**, *27*, 7168-7175

(13) Fujita, M.; Kim, W. H.; Sakanishi, Y.; Fujiwara, K.; Hirayama, S.; Okuyama, T.; Ohki, Y.; Tatsumi, K.; Yoshioka, Y. Elimination−Addition Mechanism for Nucleophilic Substitution Reaction of Cyclohexenyl Iodonium Salts and Regioselectivity of Nucleophilic Addition to the Cyclohexyne Intermediate. *J. Am. Chem. Soc.* **2004**, *126*, 7548-7558

(14) Murray, P. M.; Bellany, F.; Benhamou, L.; Bučar, D.-K.; Tabor, A. B.; Sheppard, T. D. The application of design of experiments (DoE) reaction optimisation and solvent selection in the development of new synthetic chemistry. *Org. Biomol. Chem.* **2016**, *14*, 2373-2384

(15) Hanwell, M. D.; Curtis, D. E.; Lonie, D. C.; Vandermeersch, T.; Zurek, E.; Hutchison, G. R. Avogadro: an advanced semantic chemical editor, visualization, and analysis platform. *Journal of Cheminformatics* **2012**, *4*, 17

(16) Rappe, A. K.; Casewit, C. J.; Colwell, K. S.; Goddard, W. A., III; Skiff, W. M. UFF, a full periodic table force field for molecular mechanics and molecular dynamics simulations. *J. Am. Chem. Soc.* **1992**, *114*, 10024-10035

(17) (a) Neese, F. Software Update: The ORCA Program System—Version 6.0. *WIREs Computational Molecular Science* **2025**, *15*, e70019. (b) Neese, F. An improvement of the

resolution of the identity approximation for the formation of the Coulomb matrix. *Journal of Computational Chemistry* **2003**, *24*, 1740-1747. (c) Neese, F.; Wennmohs, F.; Hansen, A.; Becker, U. Efficient, approximate and parallel Hartree–Fock and hybrid DFT calculations. A 'chain-of-spheres' algorithm for the Hartree–Fock exchange. *Chemical Physics* **2009**, *356*, 98-109. (d) 154104Grimme, S.; Antony, J.; Ehrlich, S.; Krieg, H. A consistent and accurate ab initio parametrization of density functional dispersion correction (DFT-D) for the 94 elements H-Pu. *The Journal of Chemical Physics* **2010**, *132*, . (e) Grimme, S.; Ehrlich, S.; Goerigk, L. Effect of the damping function in dispersion corrected density functional theory. *Journal of Computational Chemistry* **2011**, *32*, 1456-1465. (f) Bykov, D.; Petrenko, T.; Izsák, R.; Kossmann, S.; Becker, U.; Valeev, E.; Neese, F. Efficient implementation of the analytic second derivatives of Hartree–Fock and hybrid DFT energies: a detailed analysis of different approximations. *Molecular Physics* **2015**, *113*, 1961-1977. (g) Garcia-Ratés, M.; Neese, F. Efficient implementation of the analytical second derivatives of hartree–fock and hybrid DFT energies within the framework of the conductor-like polarizable continuum model. *Journal of Computational Chemistry* **2019**, *40*, 1816-1828. (h) Garcia-Ratés, M.; Neese, F. Effect of the Solute Cavity on the Solvation Energy and its Derivatives within the Framework of the Gaussian Charge Scheme. *Journal of Computational Chemistry* **2020**, *41*, 922-939. (i) 104109Helmich-Paris, B.; de Souza, B.; Neese, F.; Izsák, R. An improved chain of spheres for exchange algorithm. *The Journal of Chemical Physics* **2021**, *155*, . (j) Neese, F. The SHARK integral generation and digestion system. *Journal of Computational Chemistry* **2023**, *44*, 381-396

(18) Grimme, S.; Brandenburg, J. G.; Bannwarth, C.; Hansen, A. Consistent structures and interactions by density functional theory with small atomic orbital basis sets. *The Journal of Chemical Physics* **2015**, *143*,

(19) Marenich, A. V.; Cramer, C. J.; Truhlar, D. G. Universal Solvation Model Based on Solute Electron Density and on a Continuum Model of the Solvent Defined by the Bulk Dielectric Constant and Atomic Surface Tensions. *The Journal of Physical Chemistry B* **2009**, *113*, 6378-6396

NMR spectra – starting materials

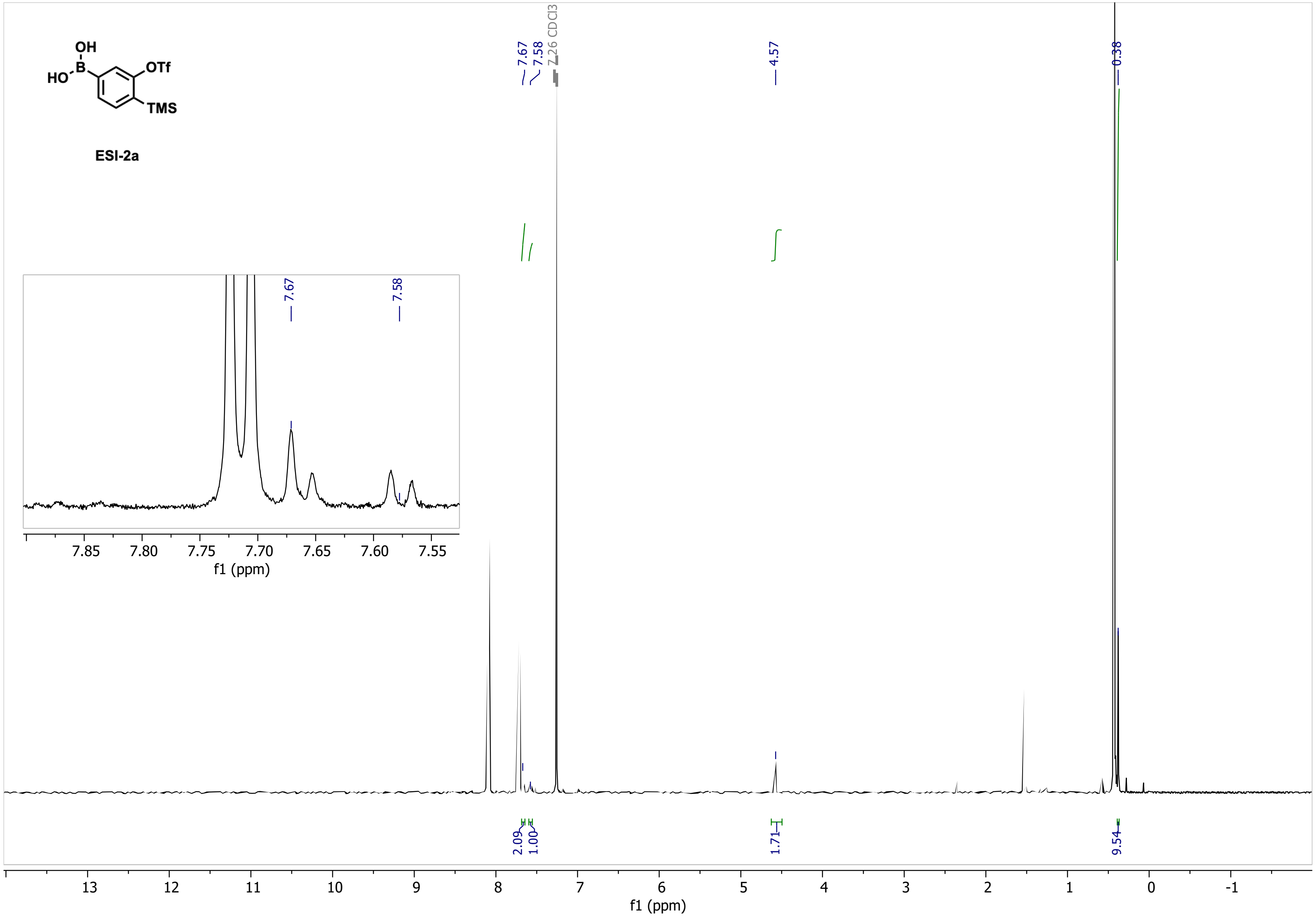

^{1}H NMR (400 MHz, $CDCl_3$) spectrum of compound **ESI-2a**.

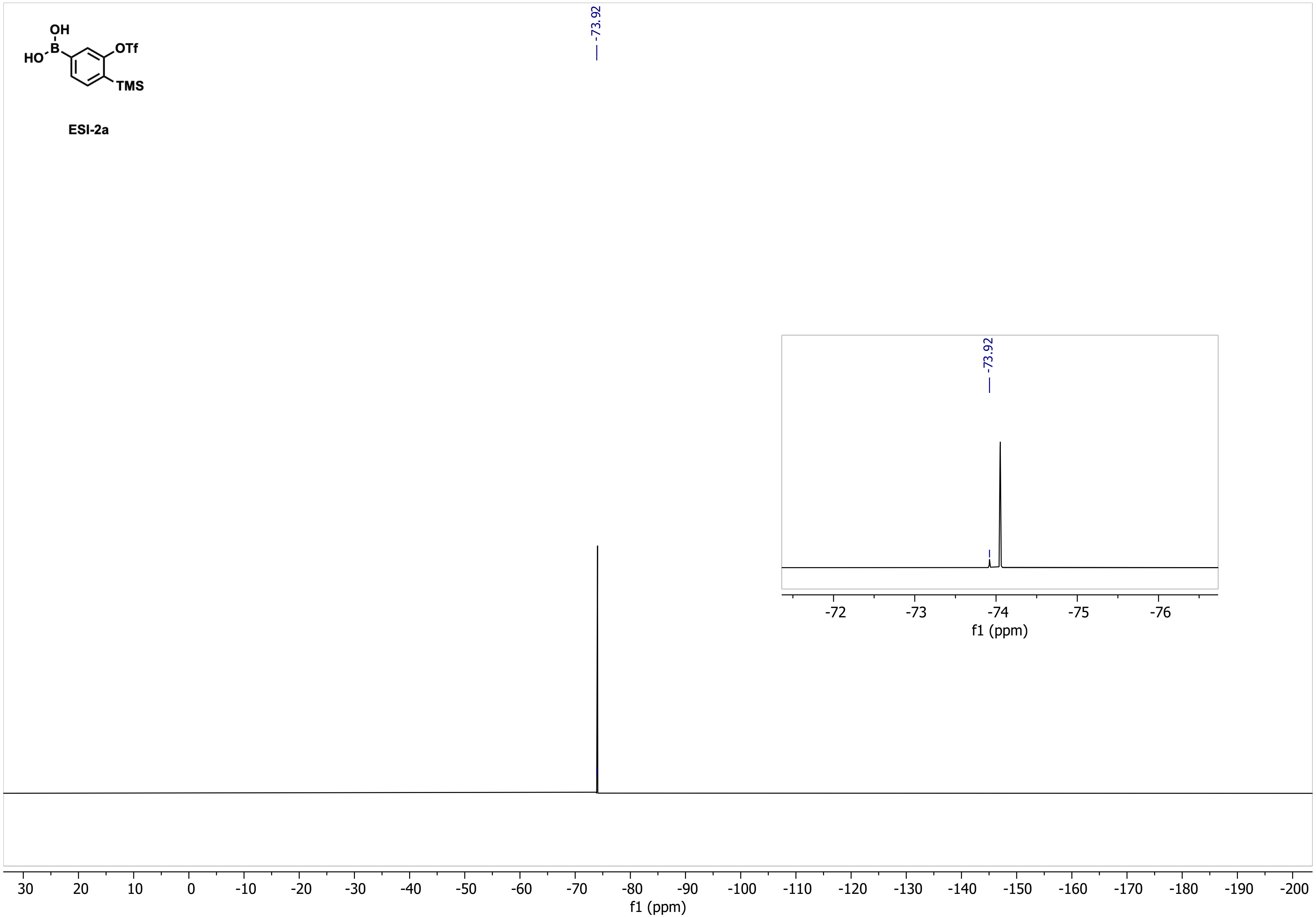

^{19}F NMR (376 MHz, $CDCl_3$) spectrum of compound **ESI-2a**.

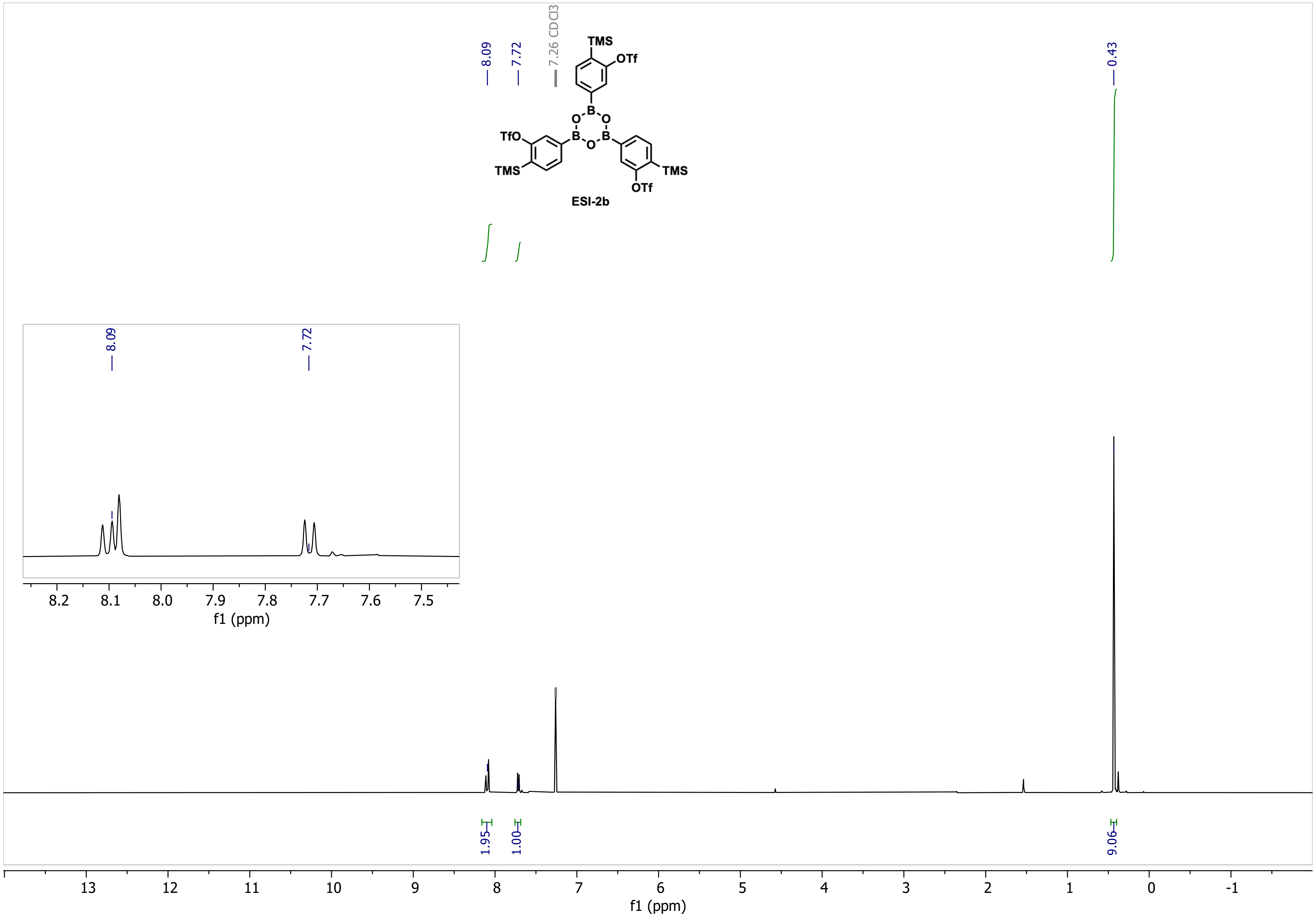

^{1}H NMR (400 MHz, $CDCl_3$) spectrum of compound **ESI-2b**.

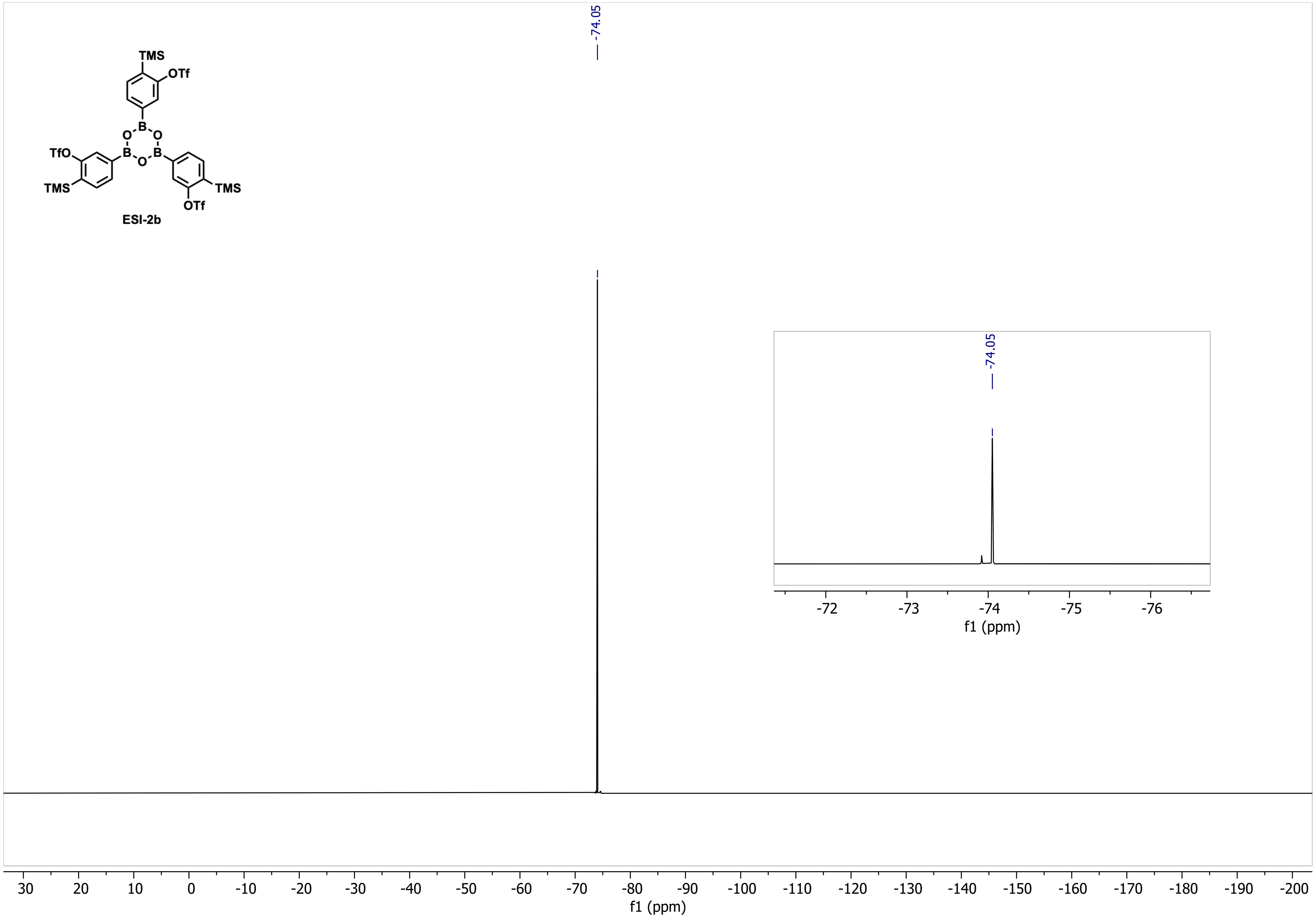

^{19}F NMR (376 MHz, $CDCl_3$) spectrum of compound **ESI-2b**.

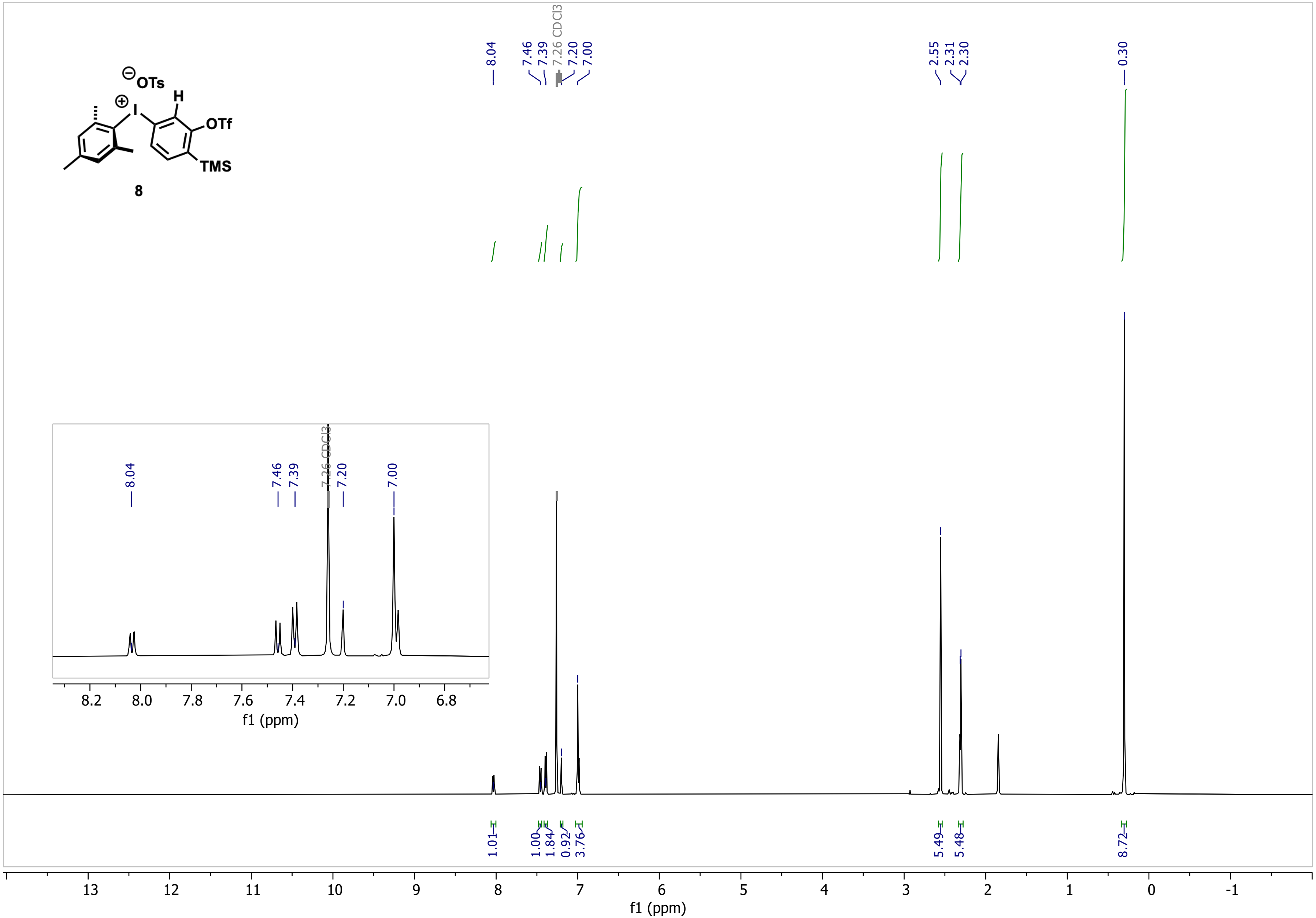

^{1}H NMR (500 MHz, $CDCl_3$) spectrum of compound **8**

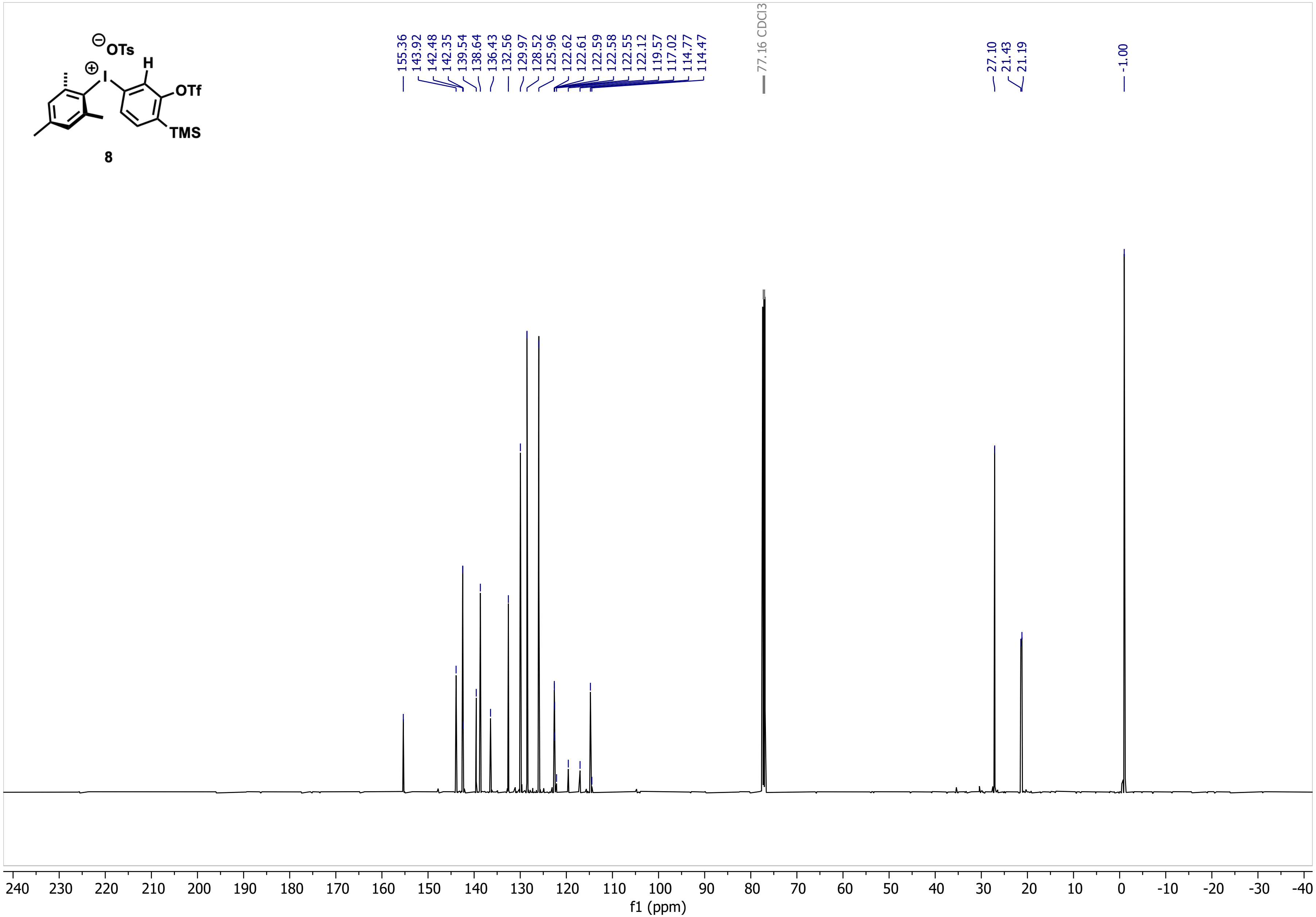

$^{13}C\{^1H\}$ NMR (126 MHz, $CDCl_3$) spectrum of compound **8**.

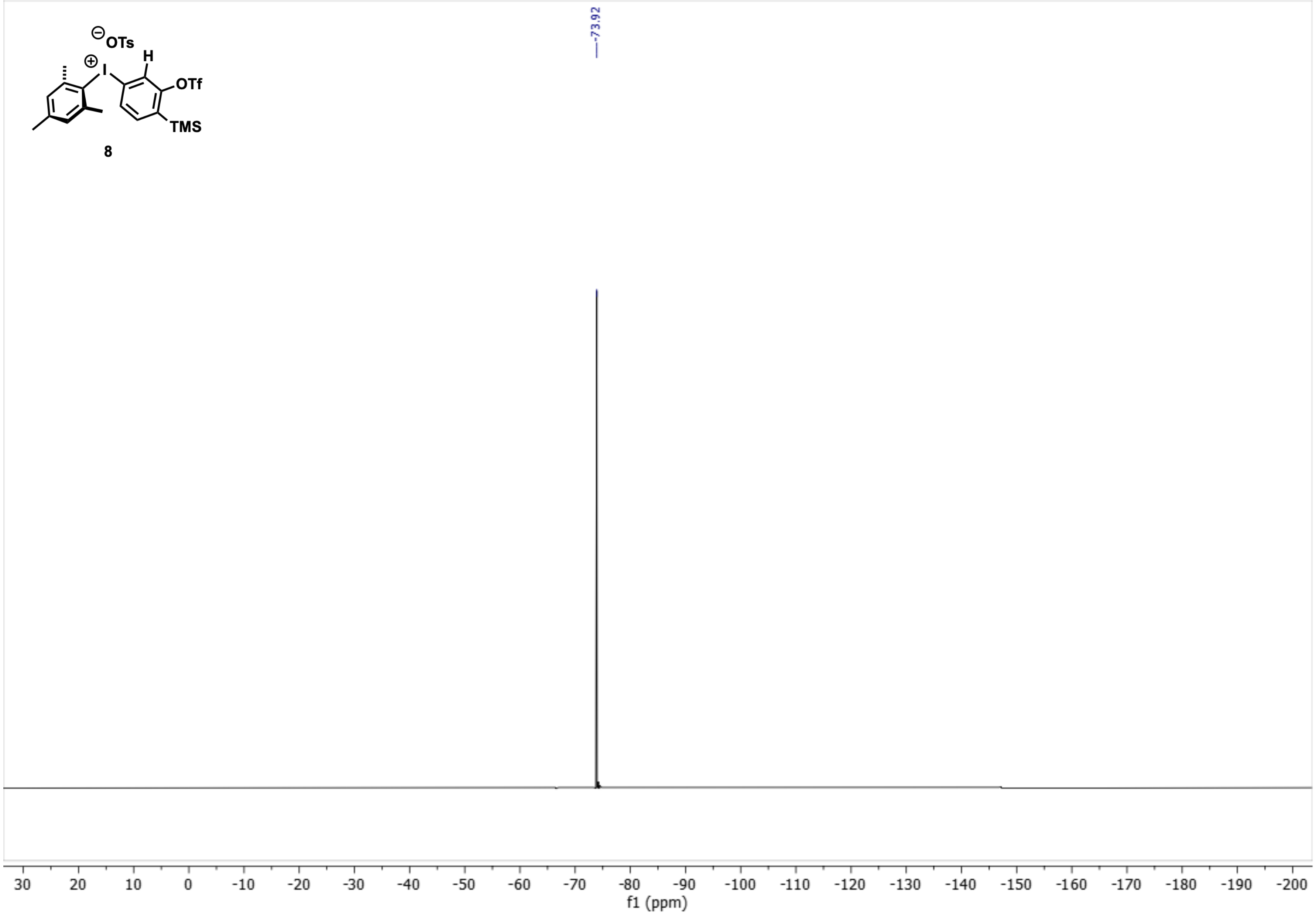

^{19}F NMR (376 MHz, $CDCl_3$) spectrum of compound **8**.

NMR spectra – Scheme 2

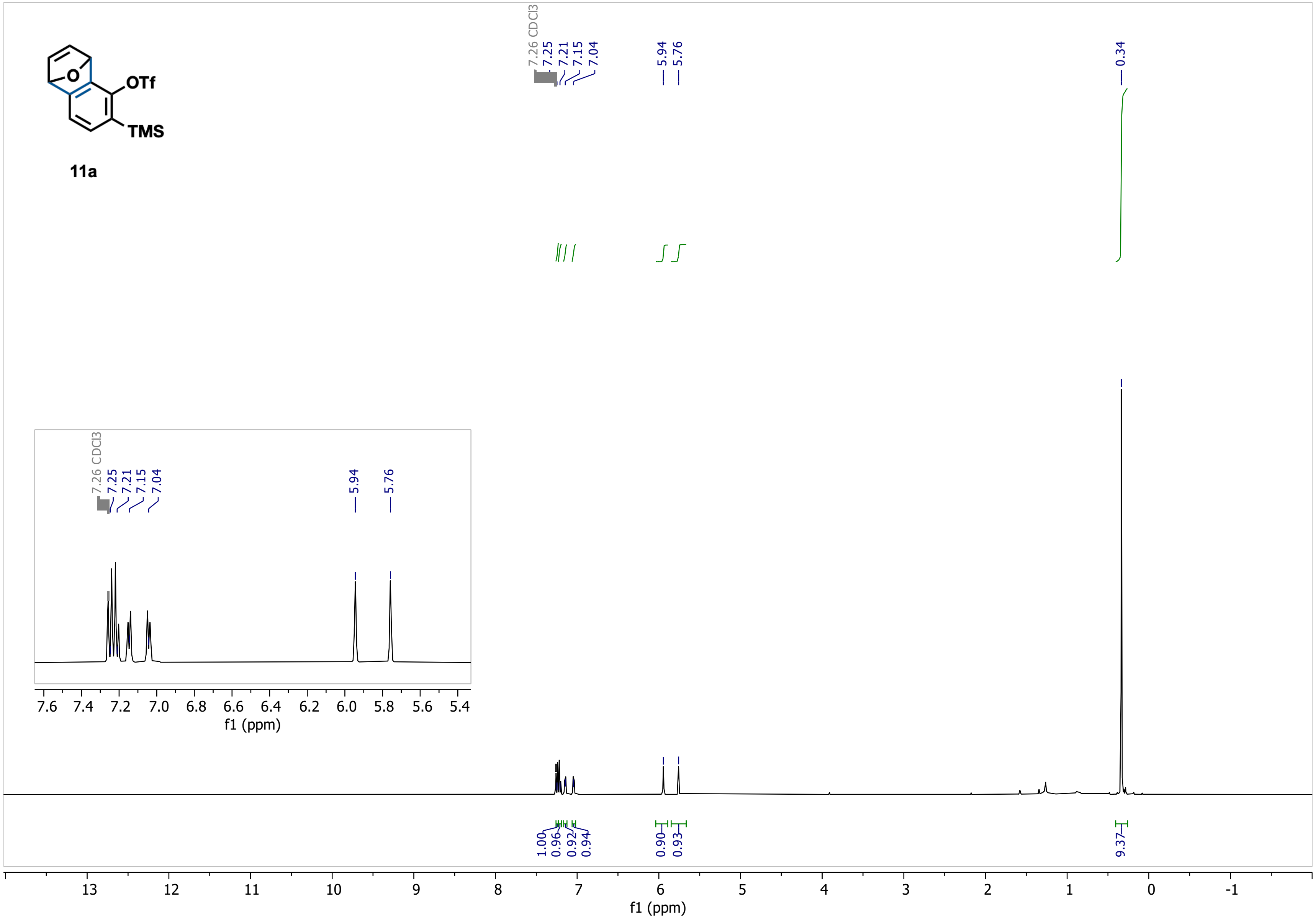

^1H NMR (400 MHz, $CDCl_3$) spectrum of compound **11a**.

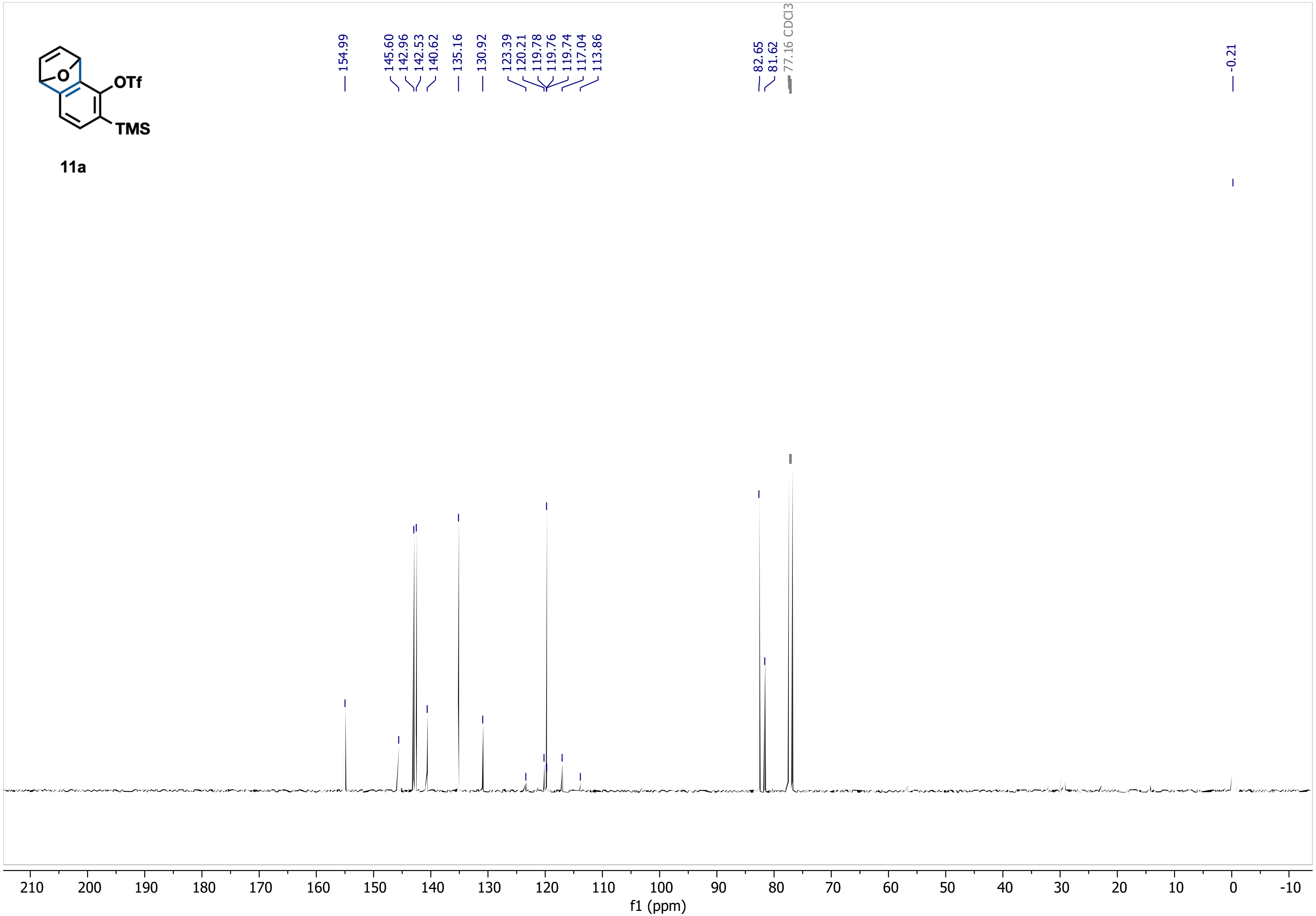

$^{13}C\{^{1}H\}$ NMR (101 MHz, $CDCl_3$) NMR spectrum of compound **11a**.

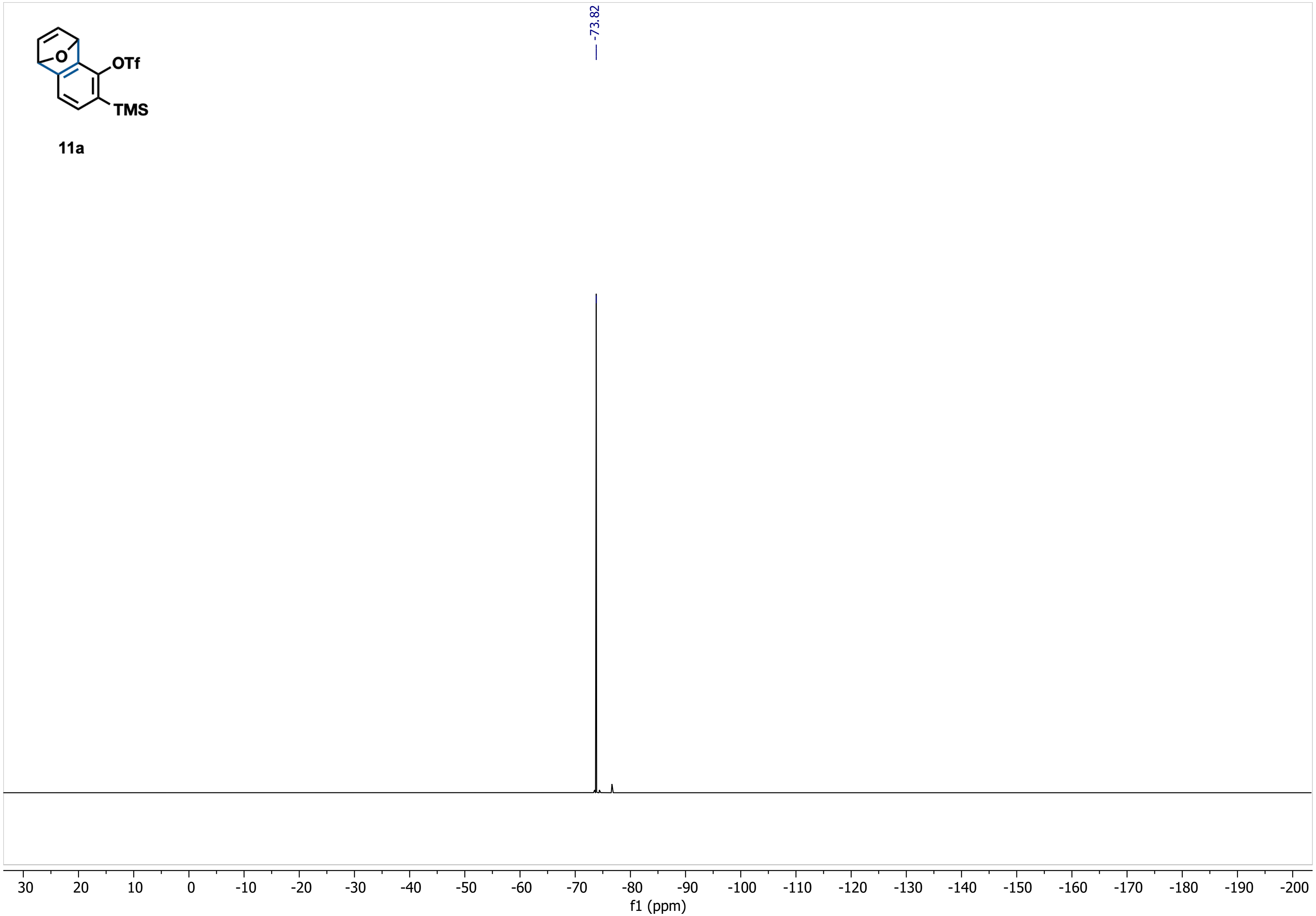

^{19}F NMR (376 MHz, $CDCl_3$) spectrum of compound **11a**.

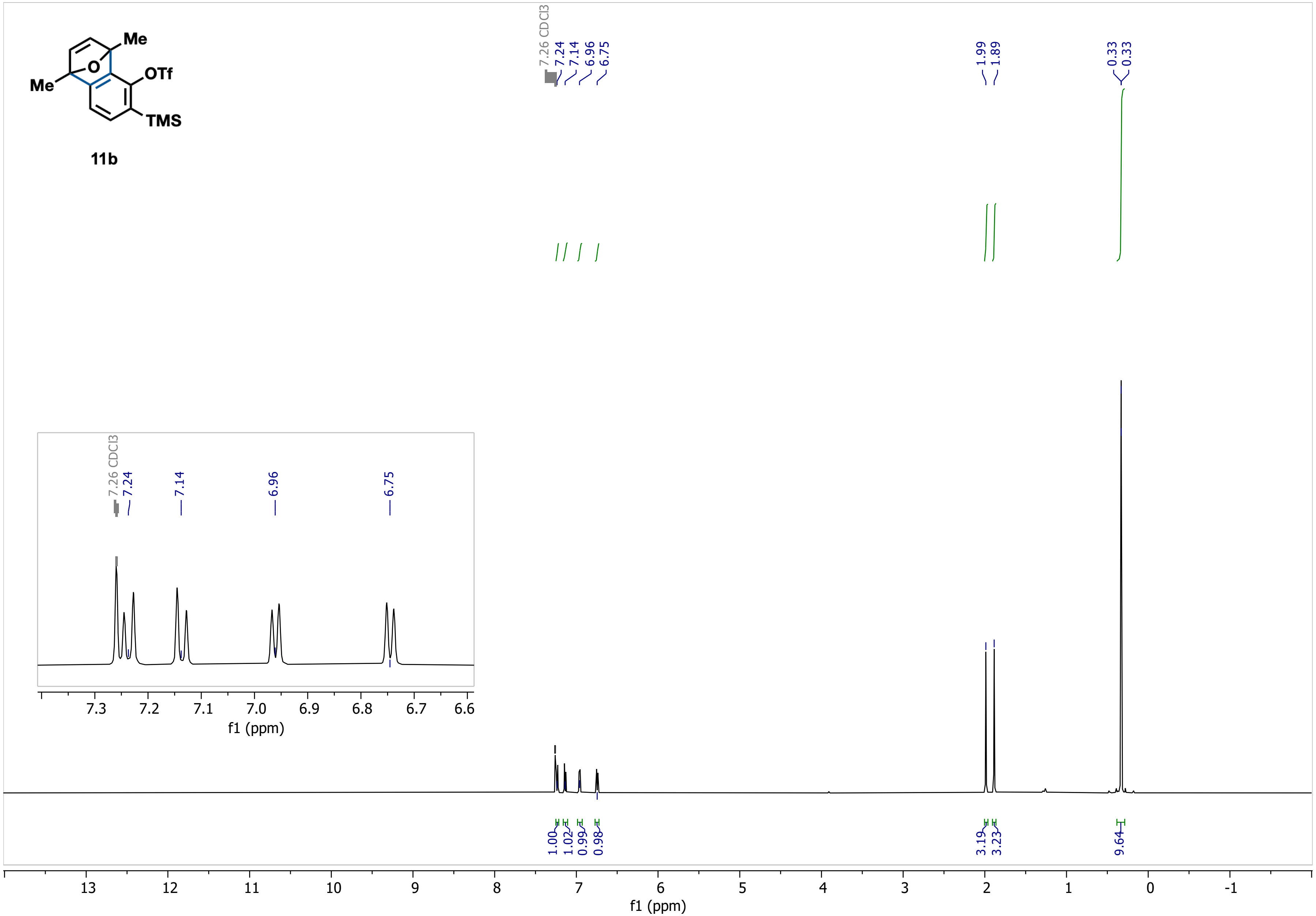

^{1}H NMR (400 MHz, $CDCl_3$) spectrum of compound **11b**.

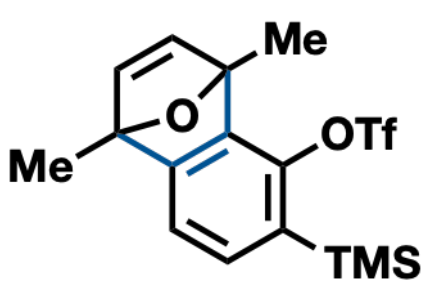

11b

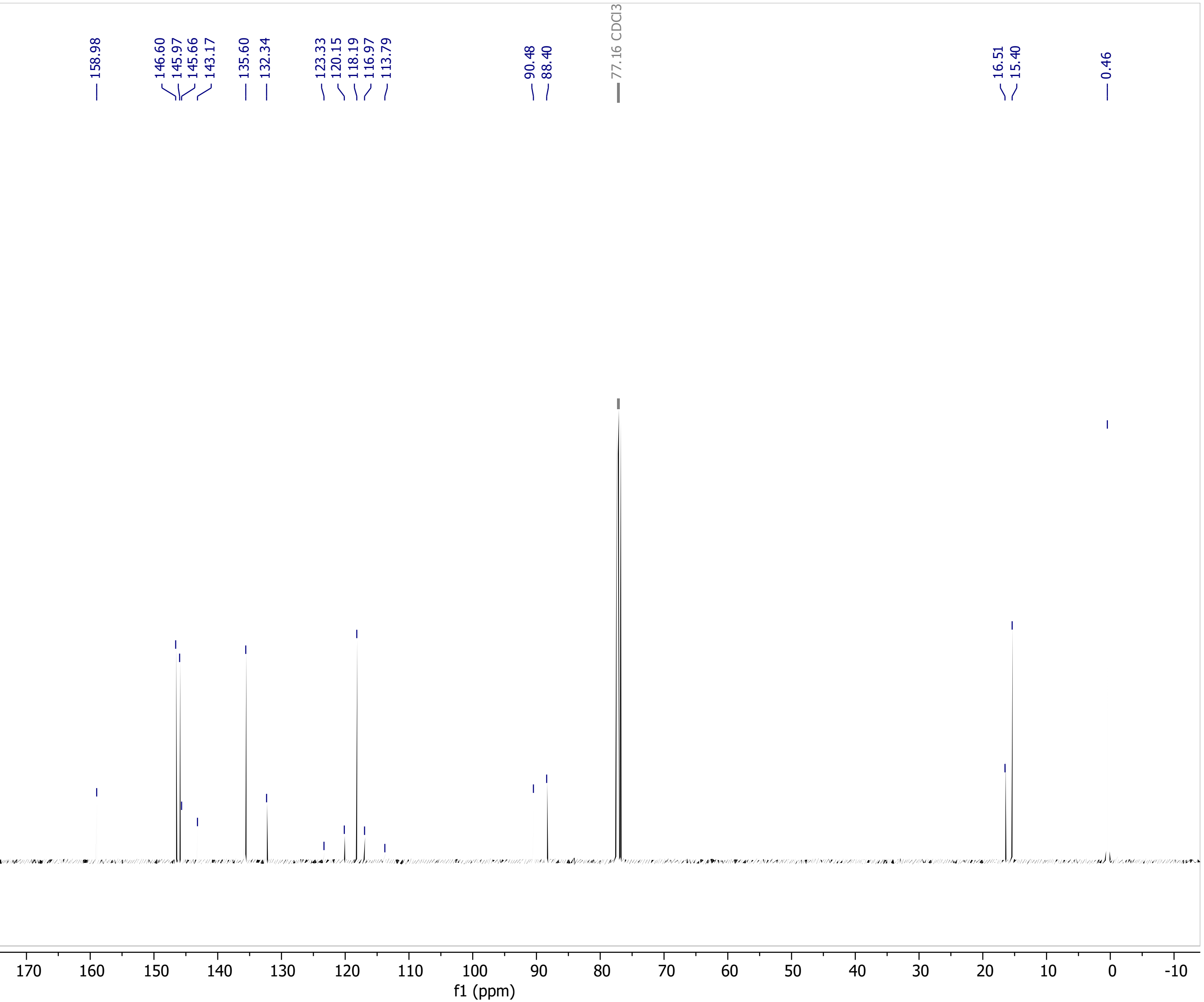

$^{13}C\{^{1}H\}$ NMR (101 MHz, $CDCl_3$) NMR spectrum of compound **11b**.

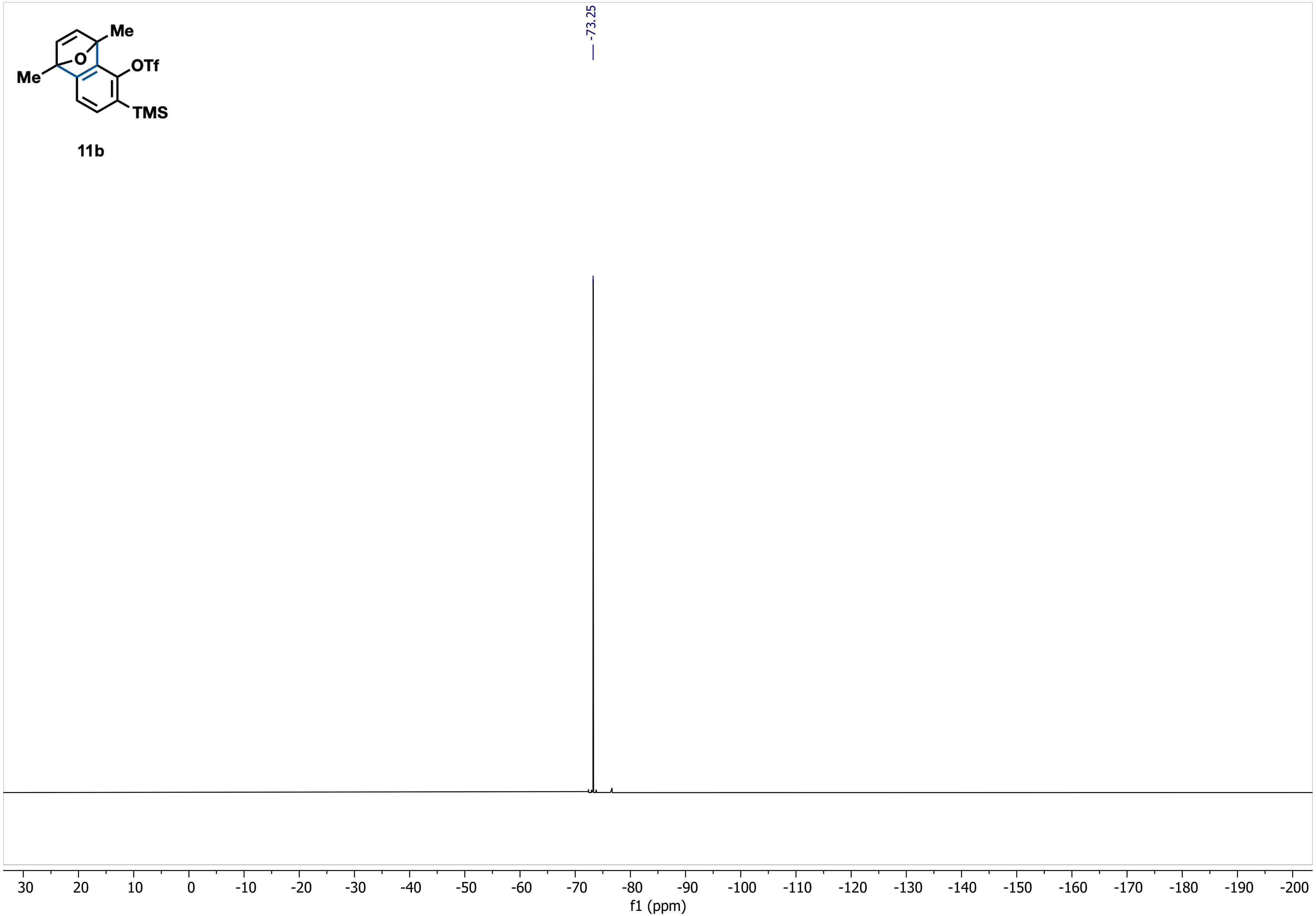

^{19}F NMR (376 MHz, $CDCl_3$) spectrum of compound **11b**.

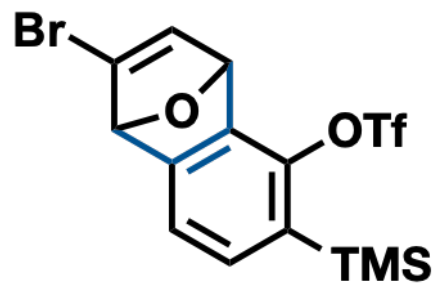

11c

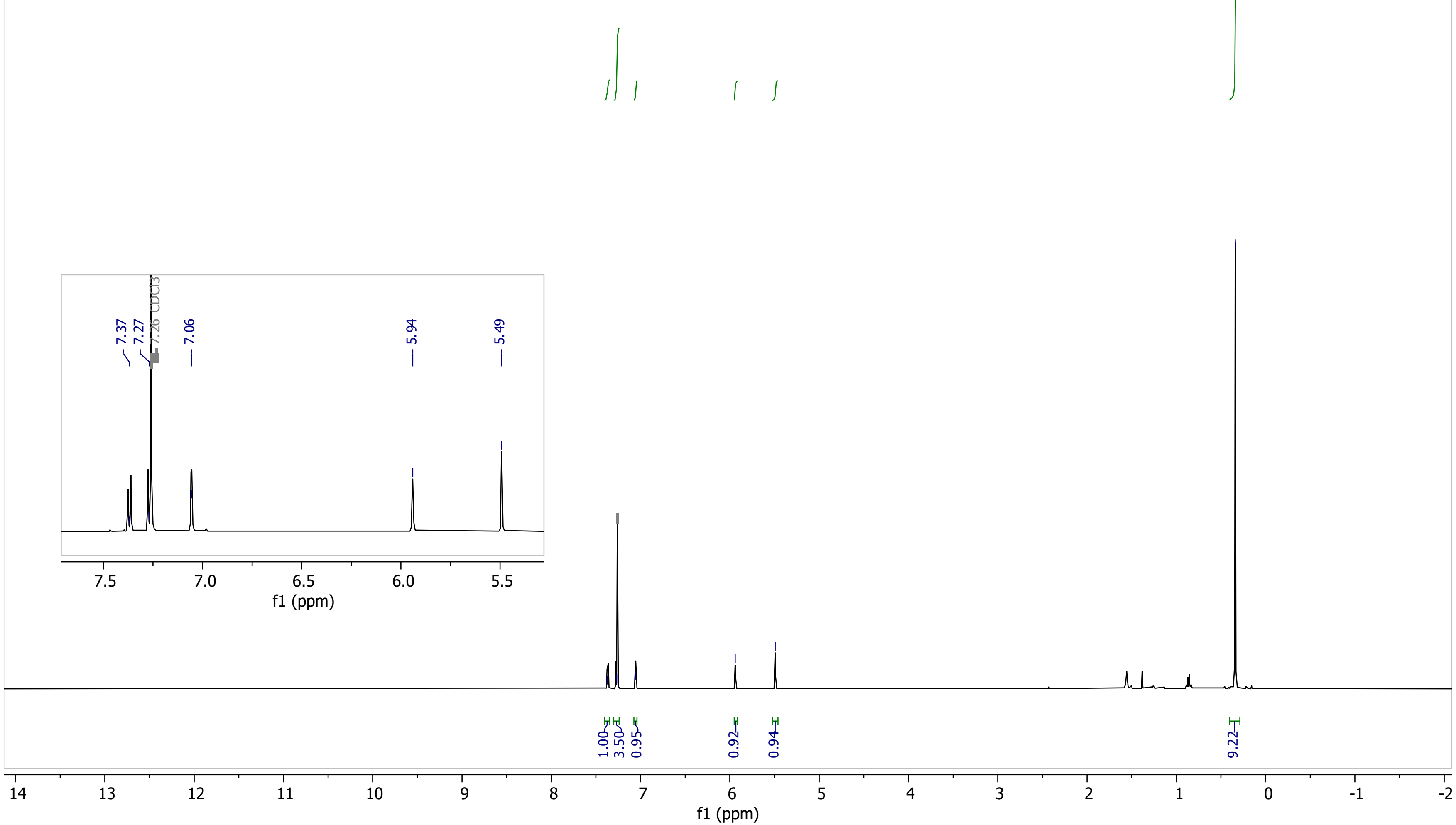

^{1}H NMR (500 MHz, $CDCl_3$) spectrum of compound **11c**.

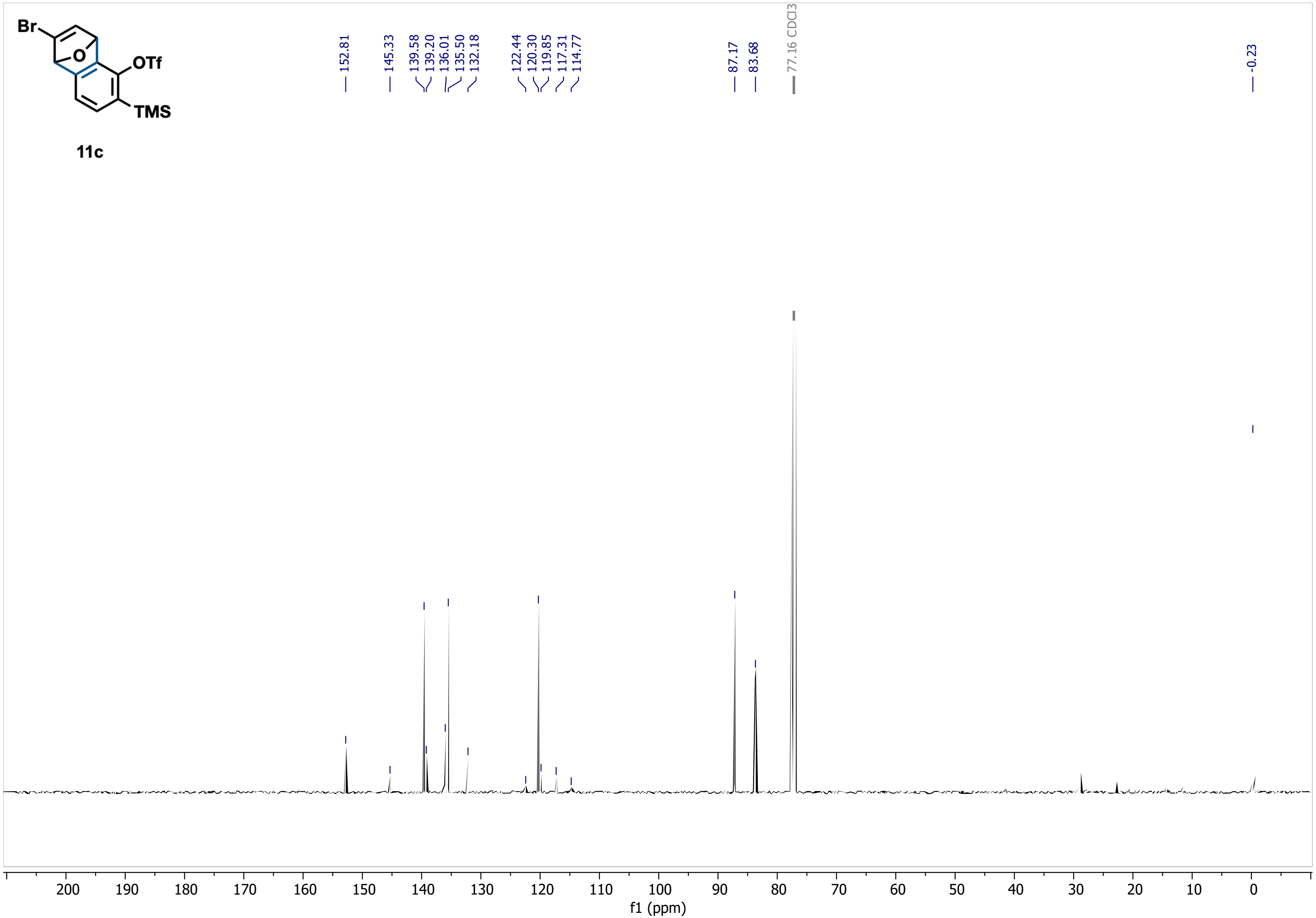

$^{13}C\{^{1}H\}$ (126 MHz, $CDCl_3$) NMR spectrum of compound **11c**.

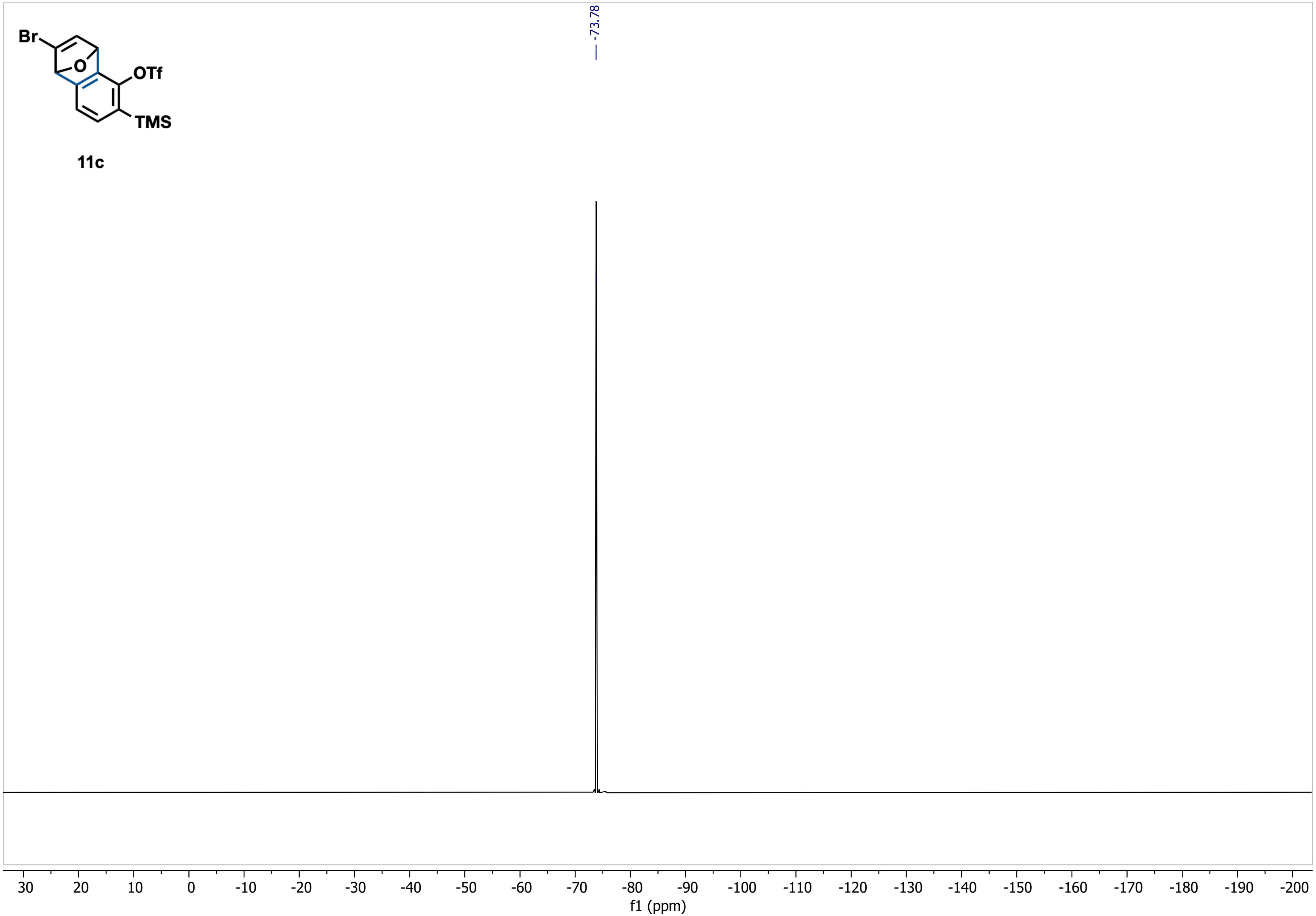

^{19}F NMR (376 MHz, $CDCl_3$) spectrum of compound **11c**.

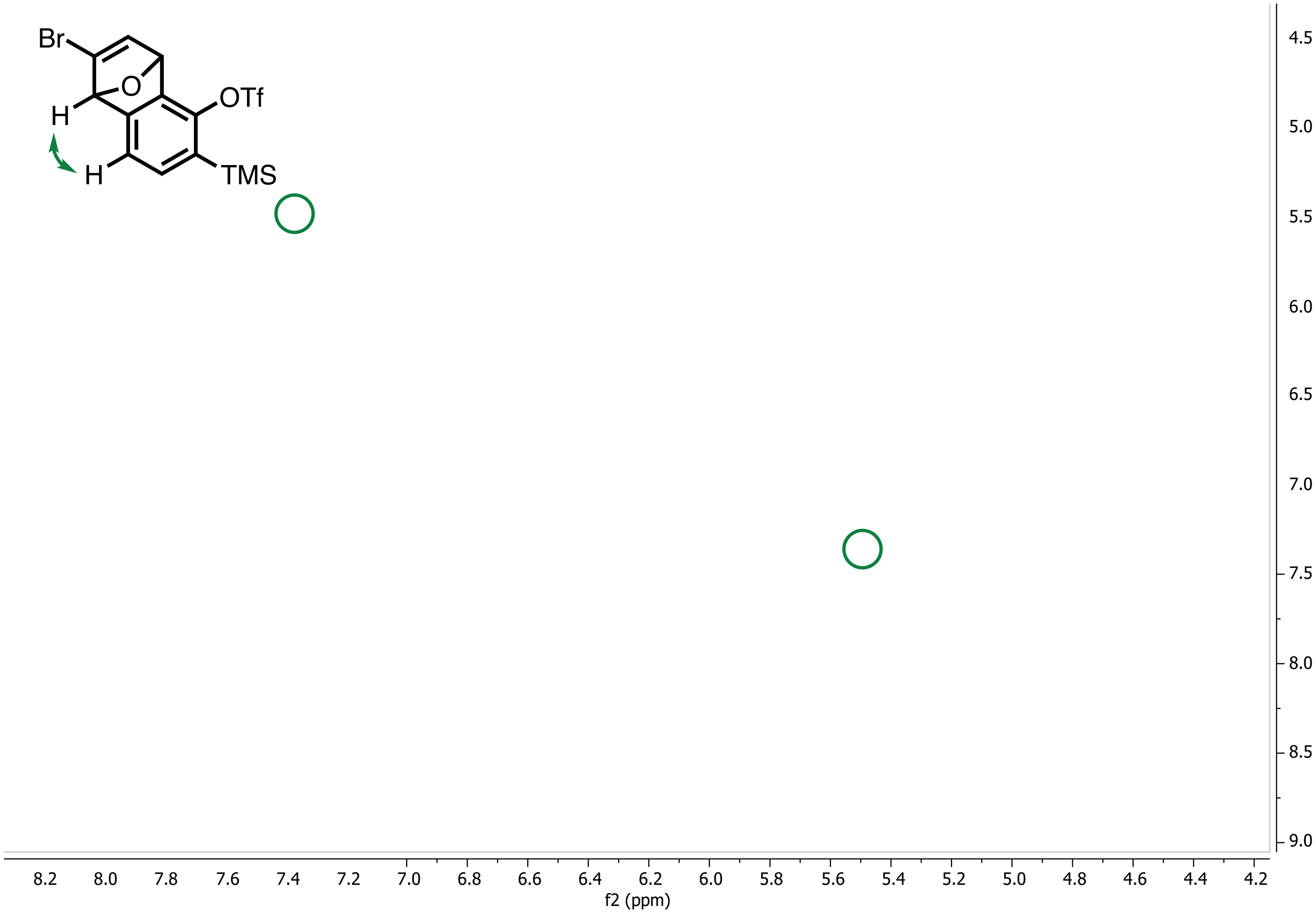

NOESY (500 MHz, $CDCl_3$) spectrum establishing regiostructure of compound **11c**.

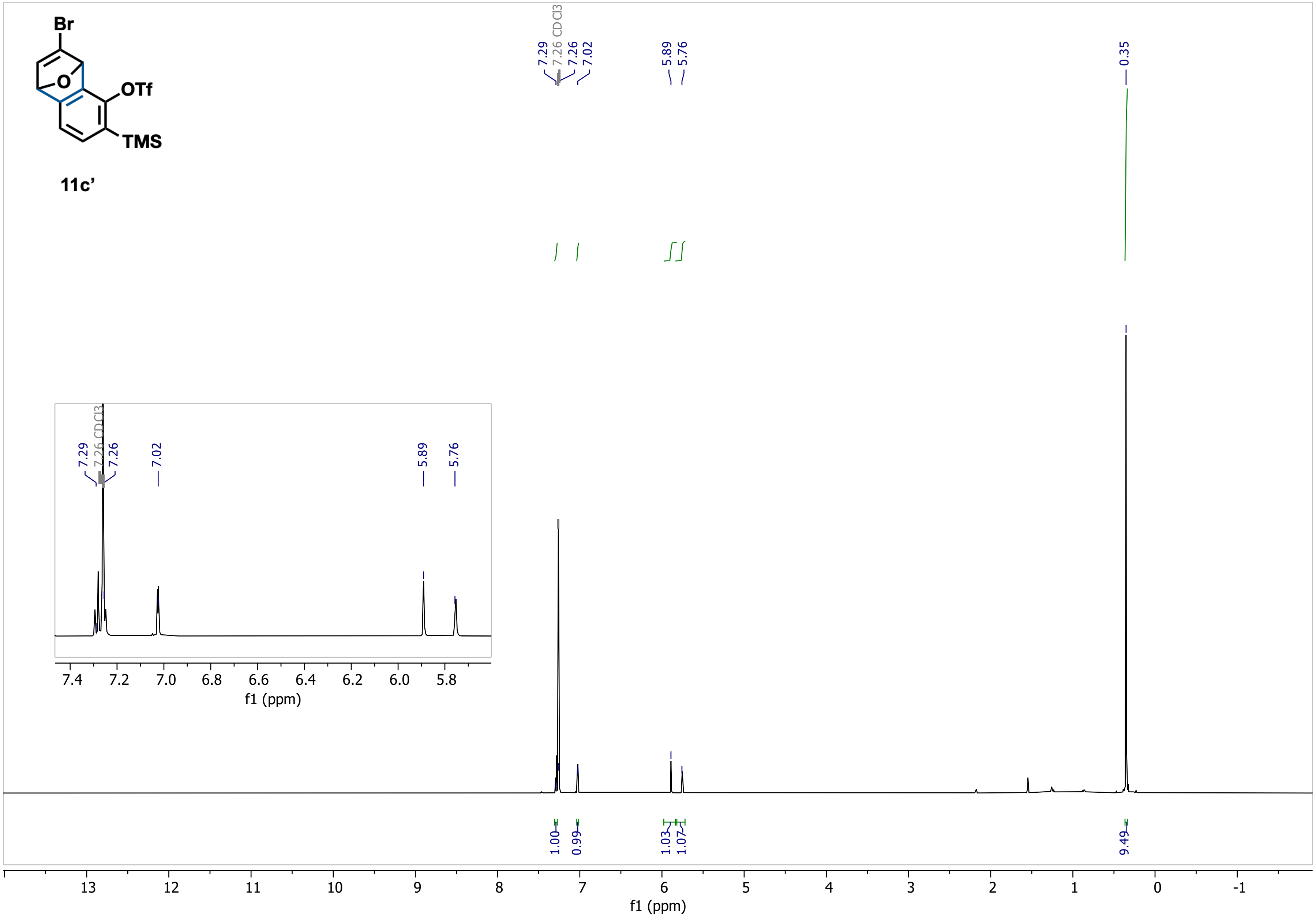

^{1}H NMR (500 MHz, $CDCl_3$) spectrum of compound **11c'**.

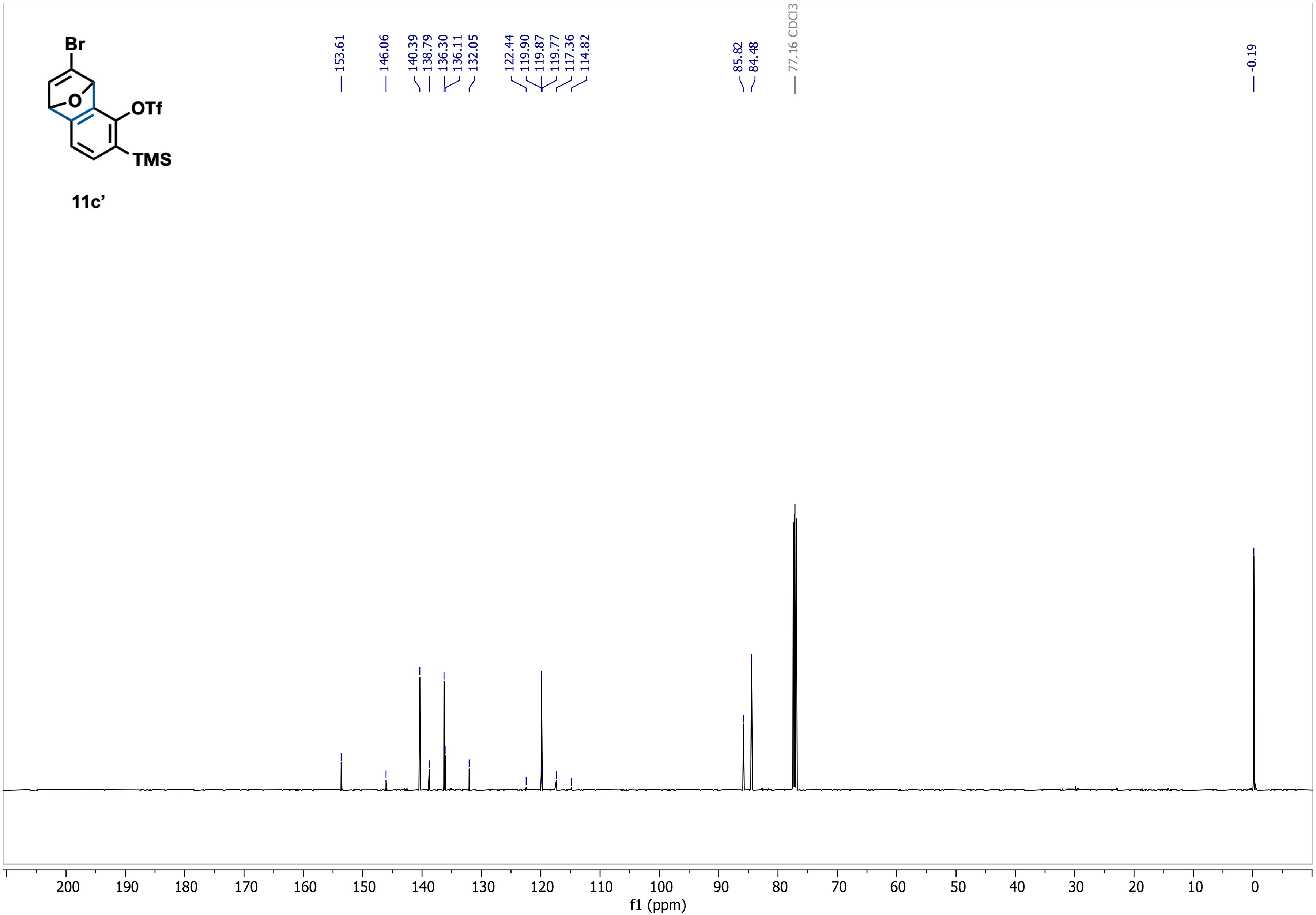

$^{13}C\{^{1}H\}$ NMR (126 MHz, $CDCl_3$) spectrum of compound **11c'**.

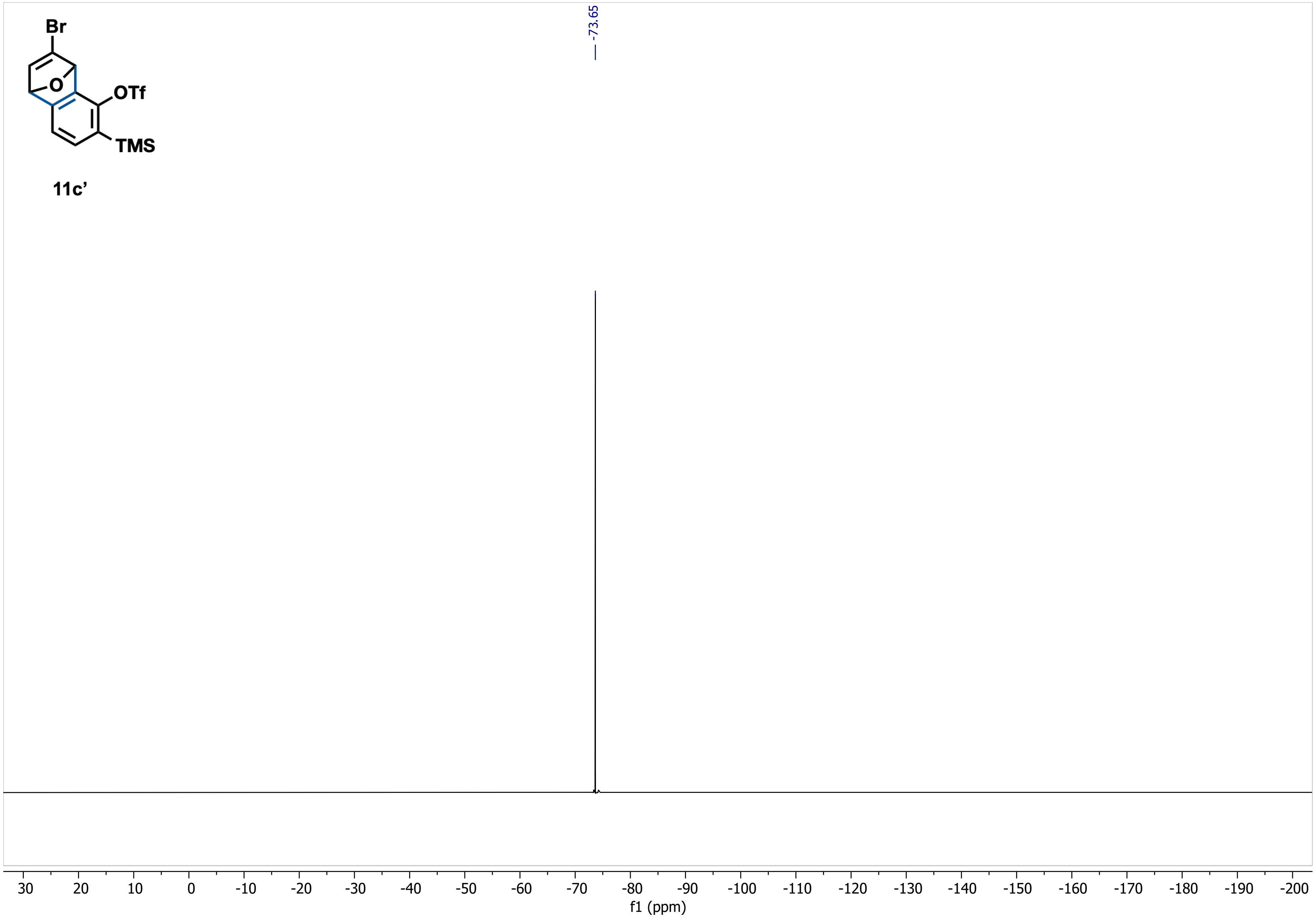

^{19}F NMR (376 MHz, $CDCl_3$) spectrum of compound **11c'**.

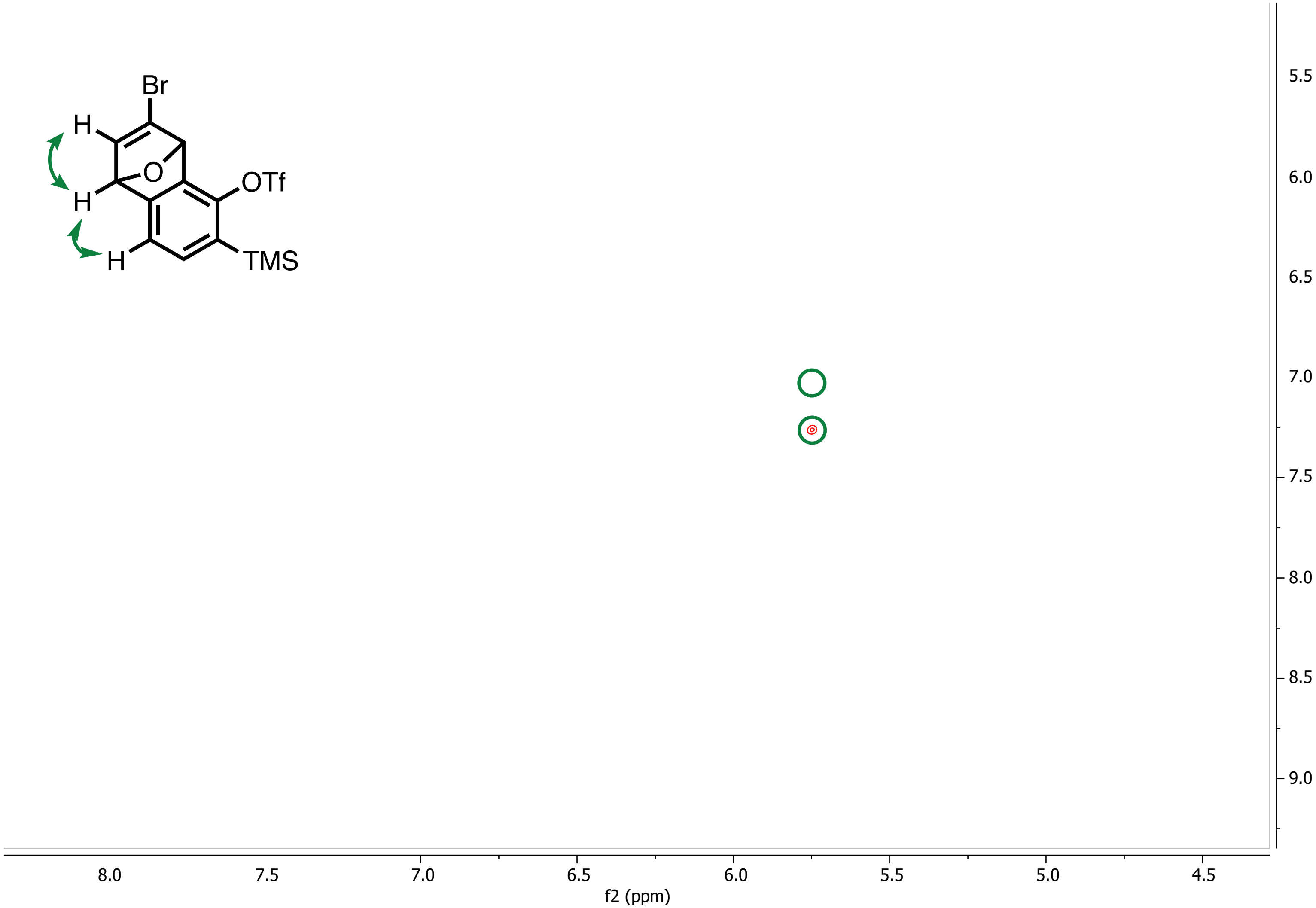

NOESY (500 MHz, $CDCl_3$) spectrum and proof of regiostructure of compound **11c'**.

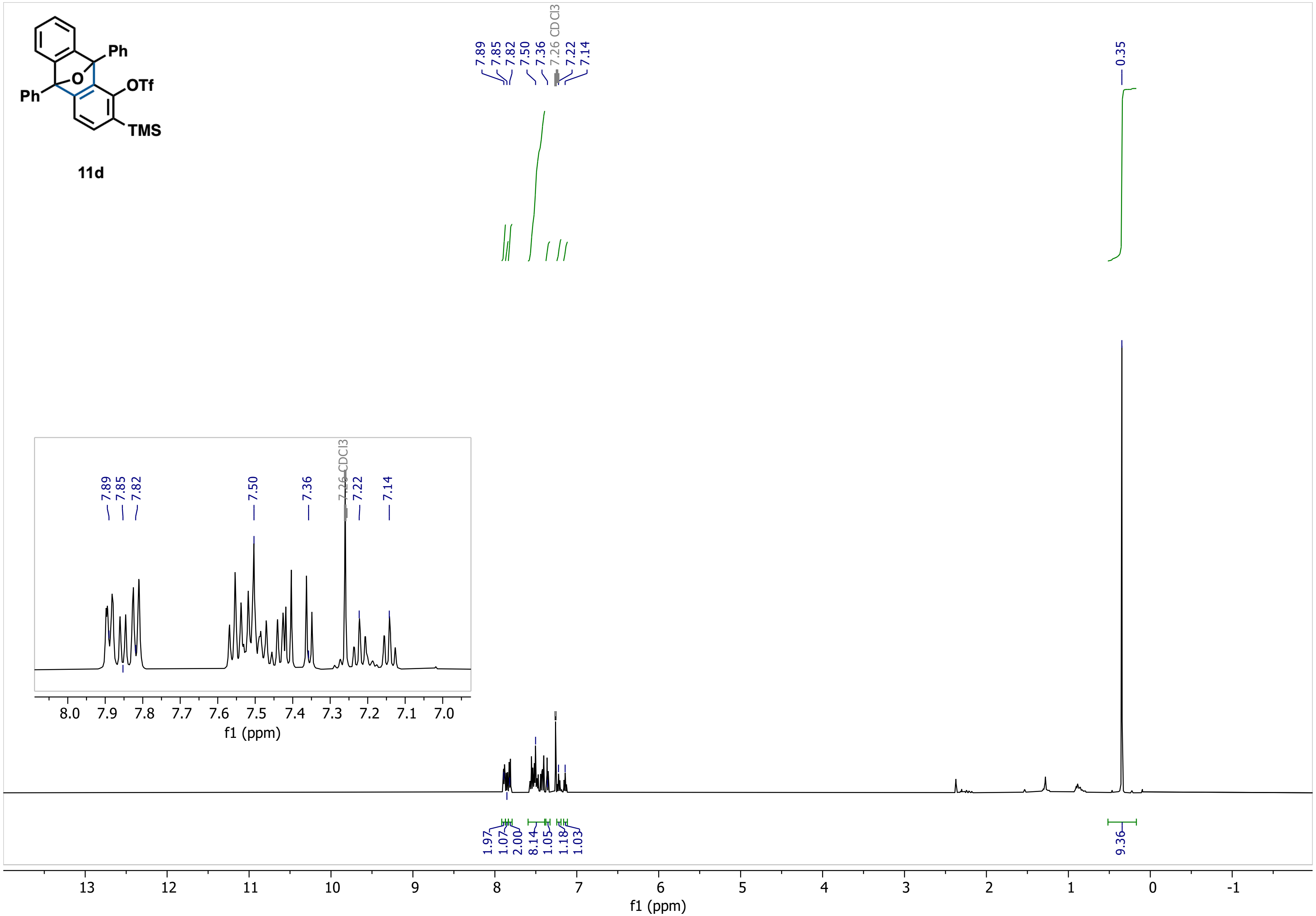

^{1}H NMR (500 MHz, $CDCl_3$) spectrum of compound **11d**.

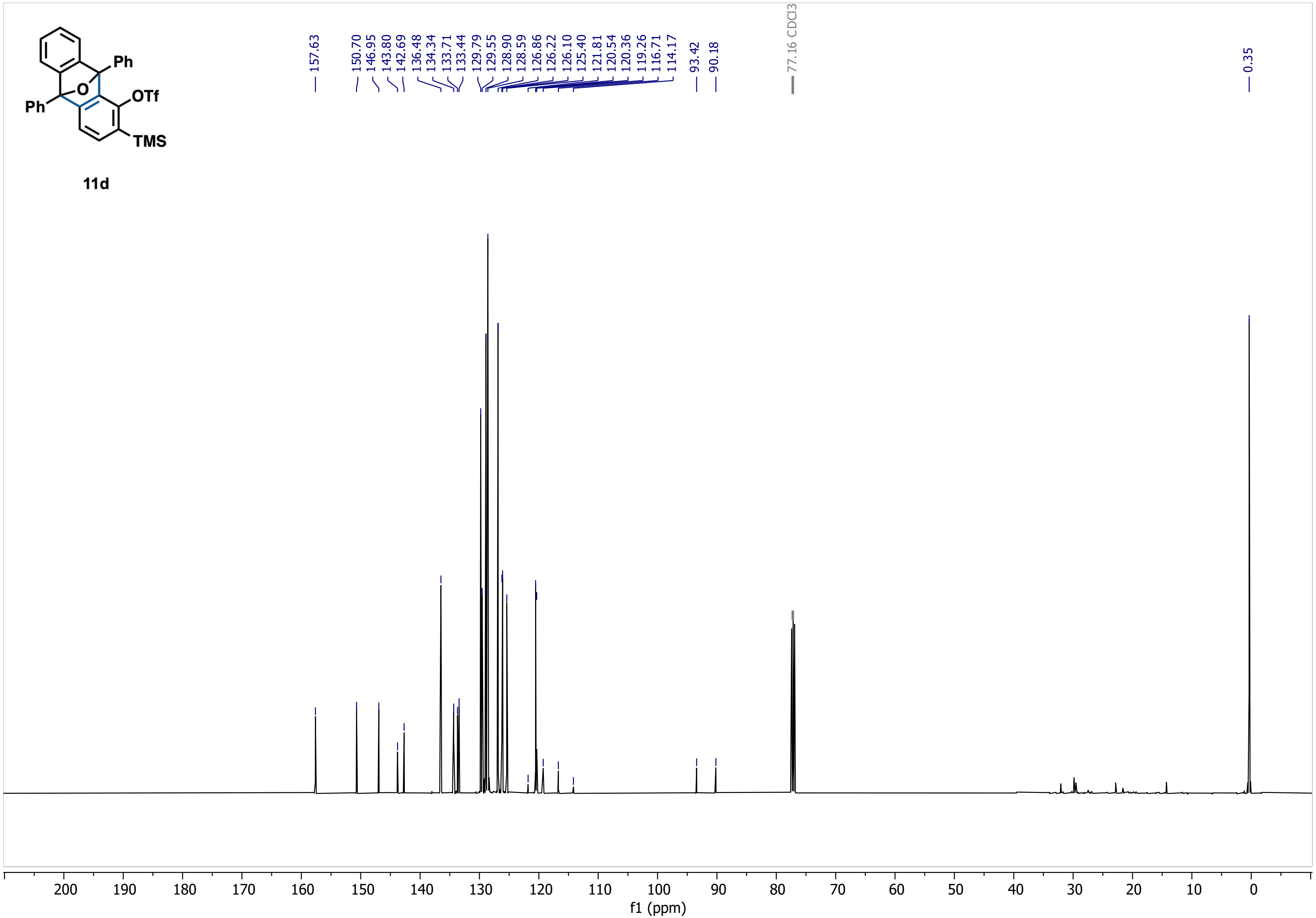

^{113}C{^{1}H} NMR (126 MHz, $CDCl_3$) NMR spectrum of compound **11d**.

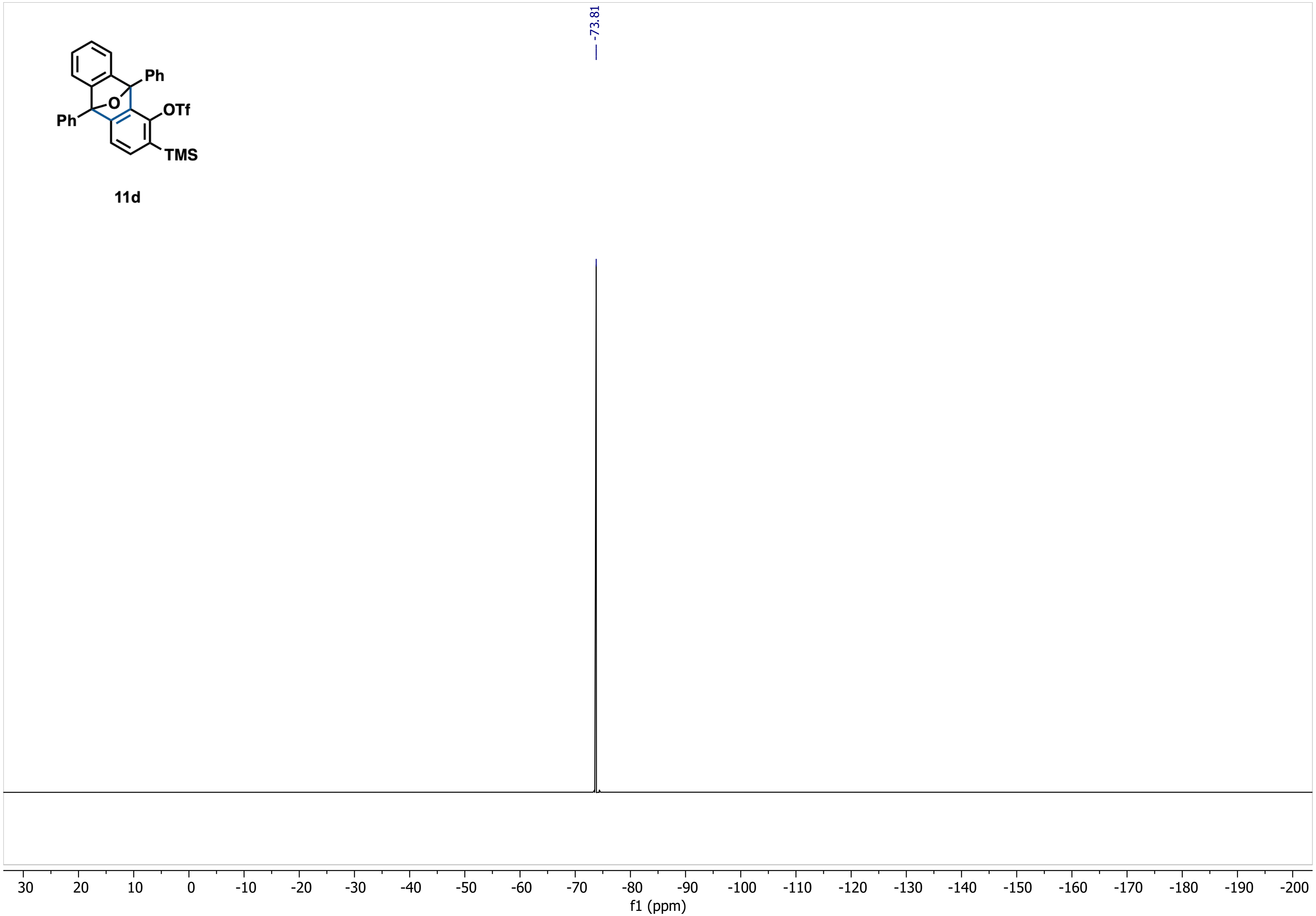

^{19}F NMR (376 MHz, $CDCl_3$) spectrum of compound **11d**.

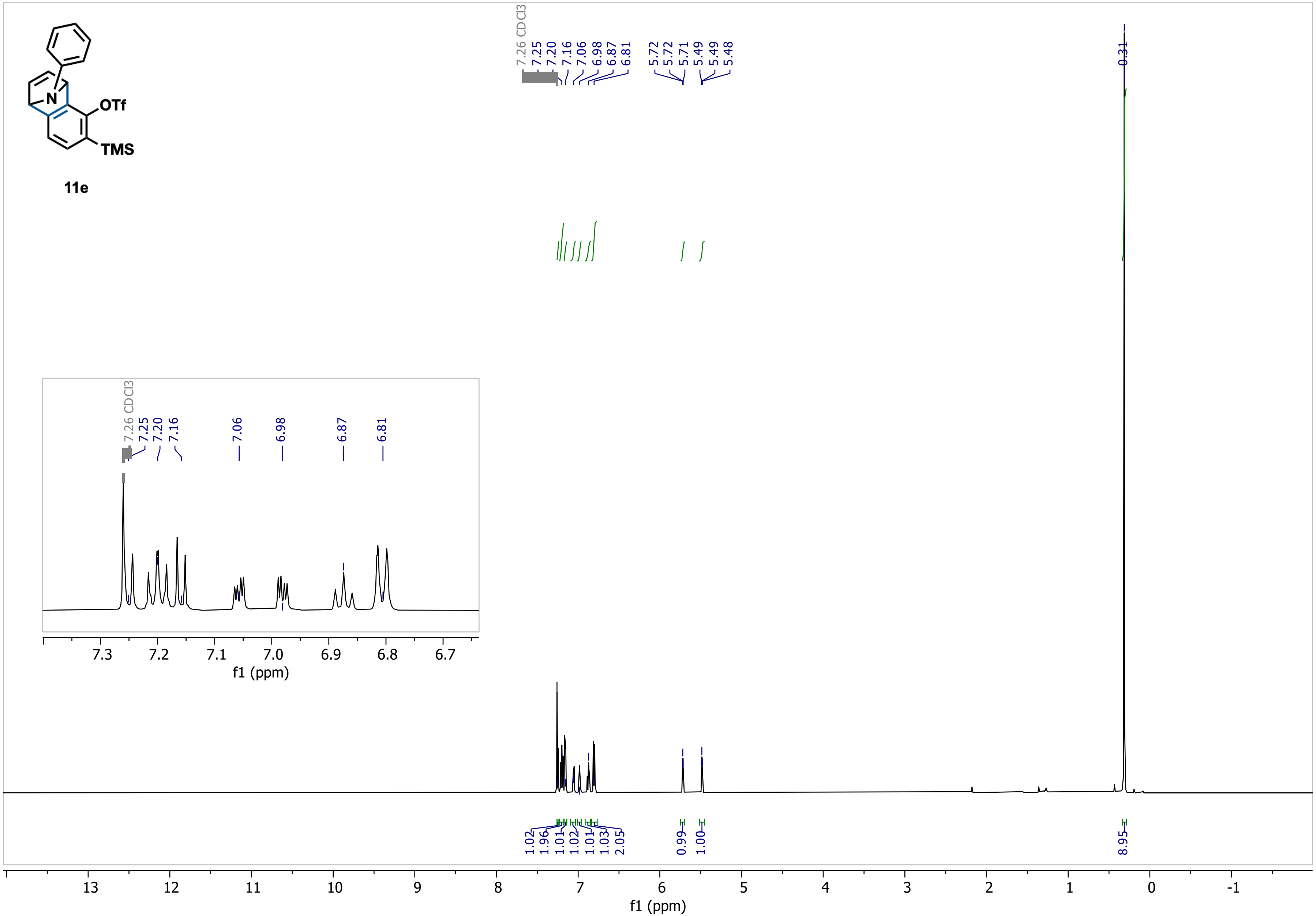

^{1}H NMR (500 MHz, $CDCl_3$) spectrum of compound **11e**.

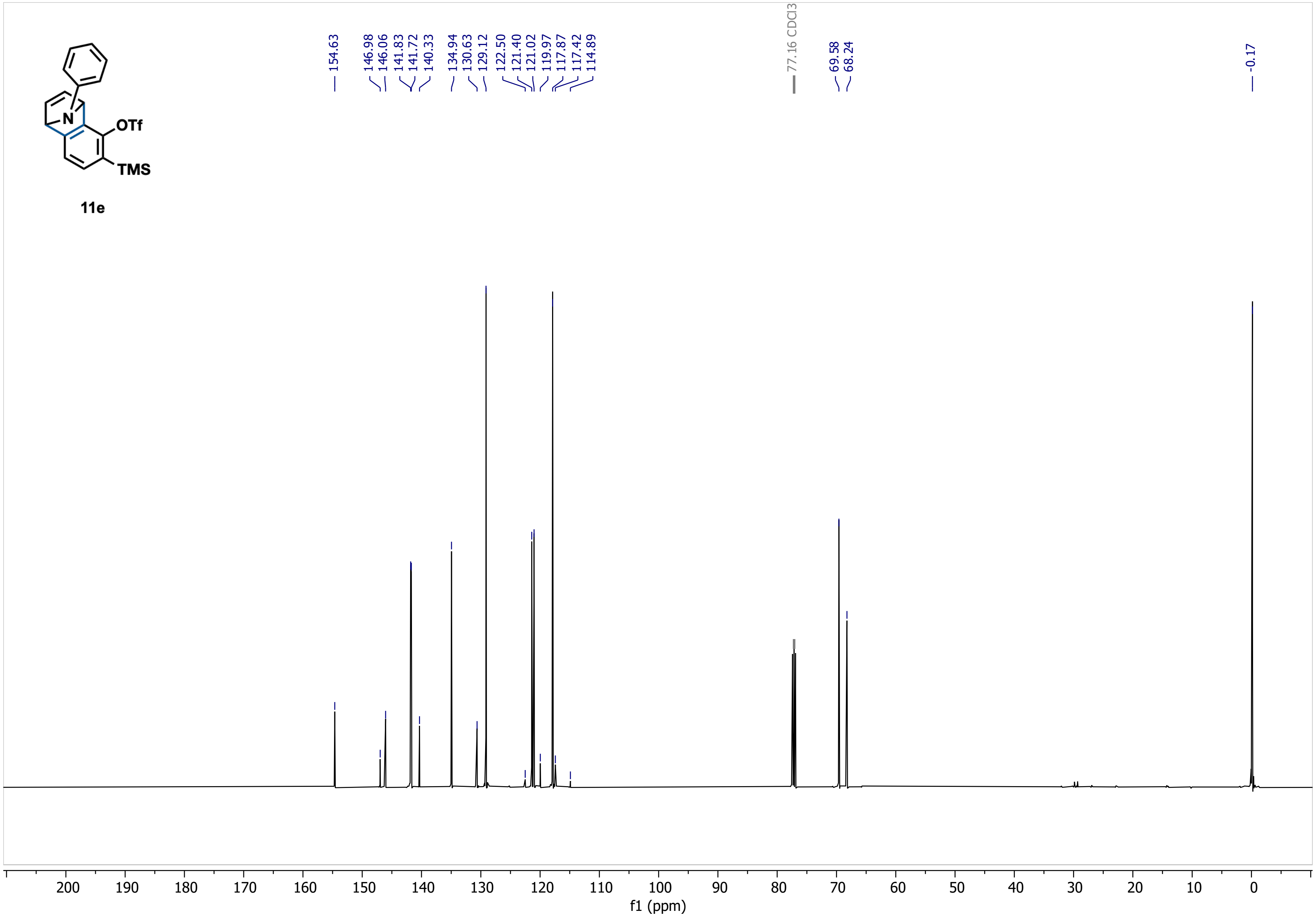

$^{13}C\{^{1}H\}$ NMR (126 MHz, $CDCl_3$) NMR spectrum of compound **11e.**

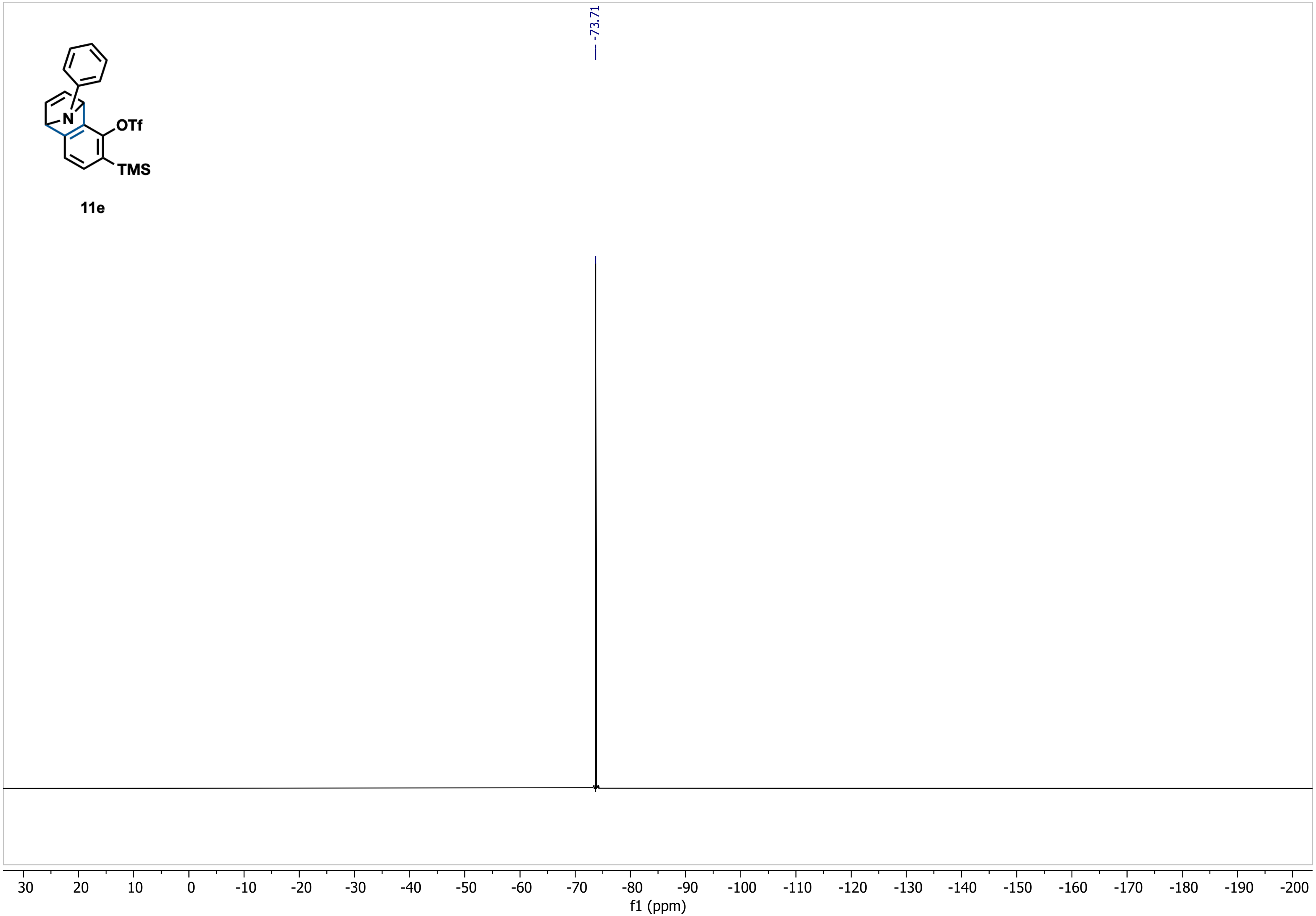

^{19}F NMR (376 MHz, $CDCl_3$) spectrum of compound **11e**.

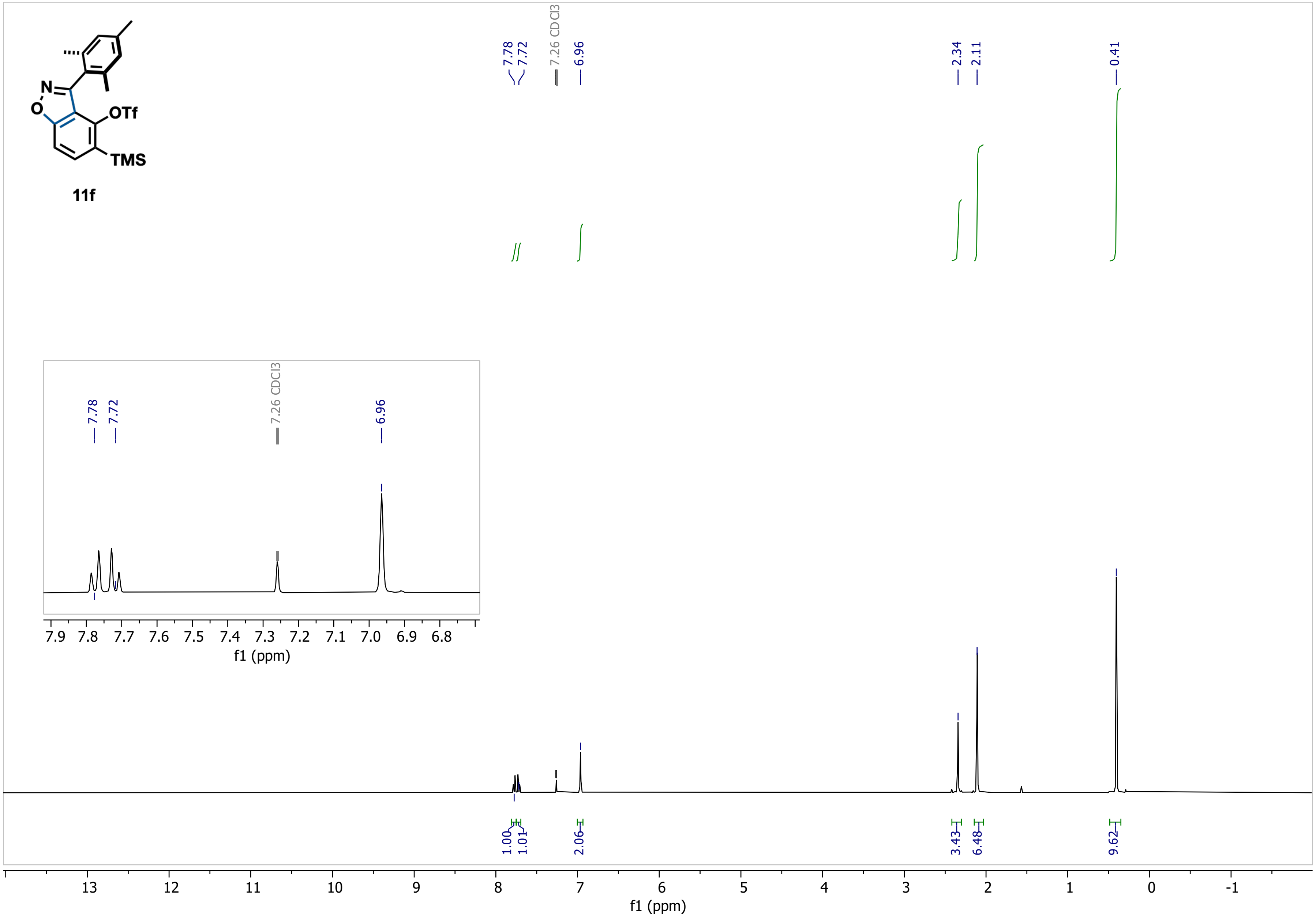

^{1}H NMR (400 MHz, $CDCl_3$) spectrum of compound **11f**.

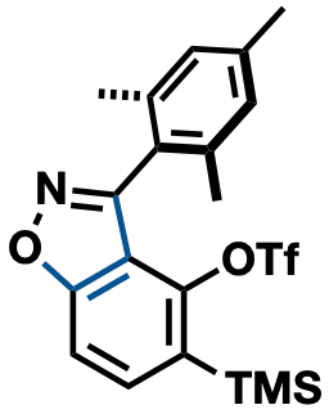

11f

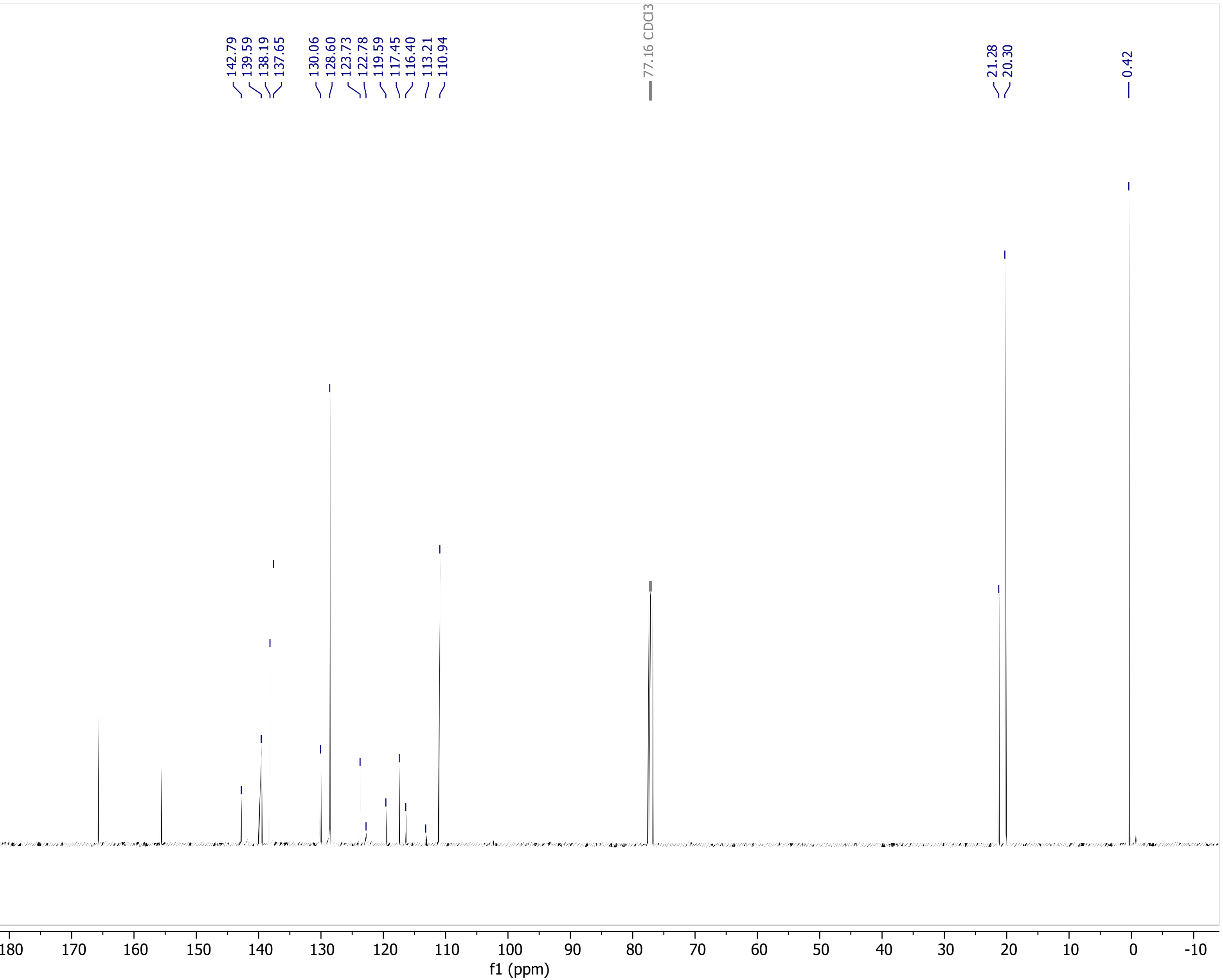

$^{13}C\{^{1}H\}$ NMR (101 MHz, $CDCl_3$) spectrum of compound **11f**.

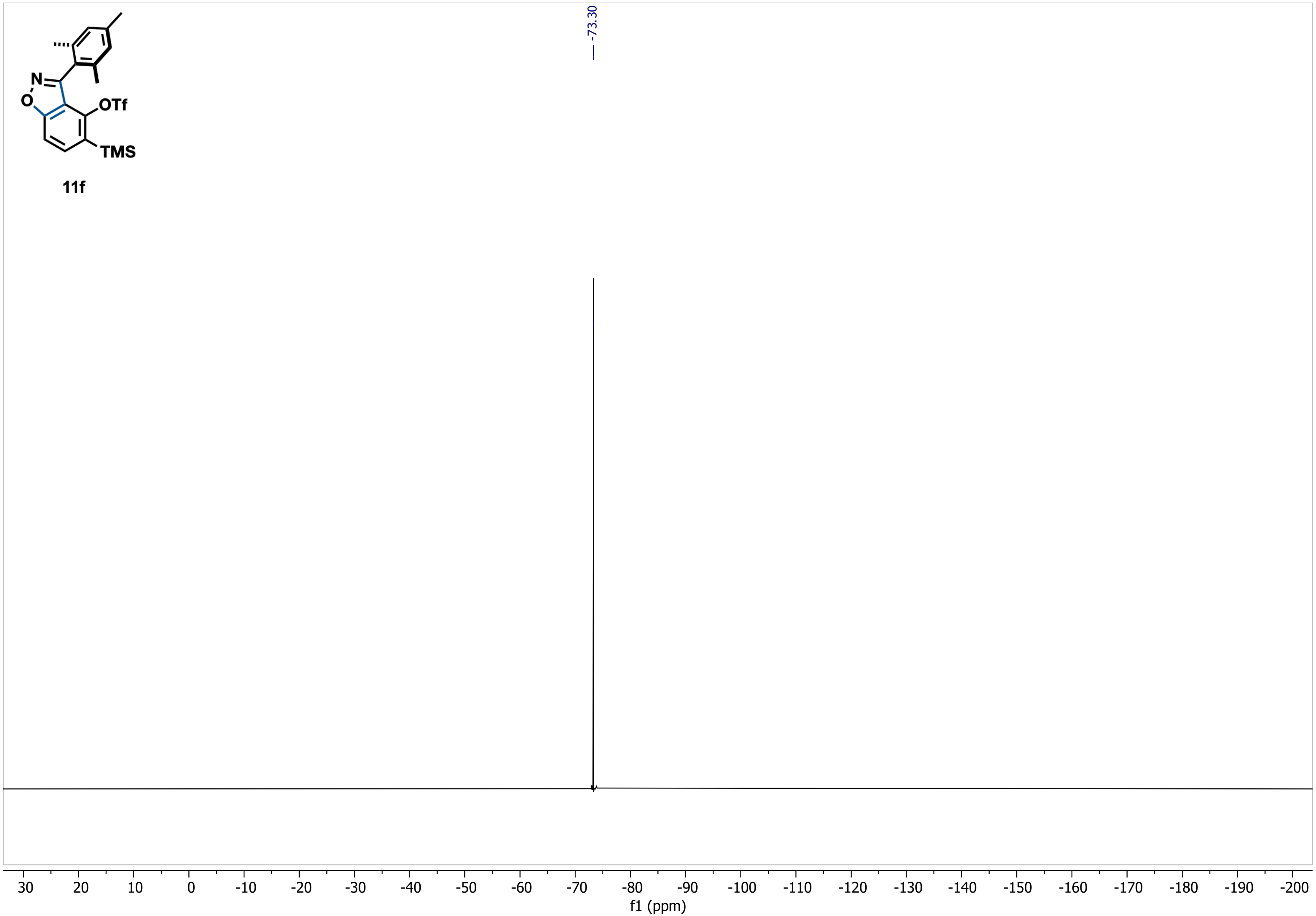

^{19}F NMR (376 MHz, $CDCl_3$) spectrum of compound **11f**.

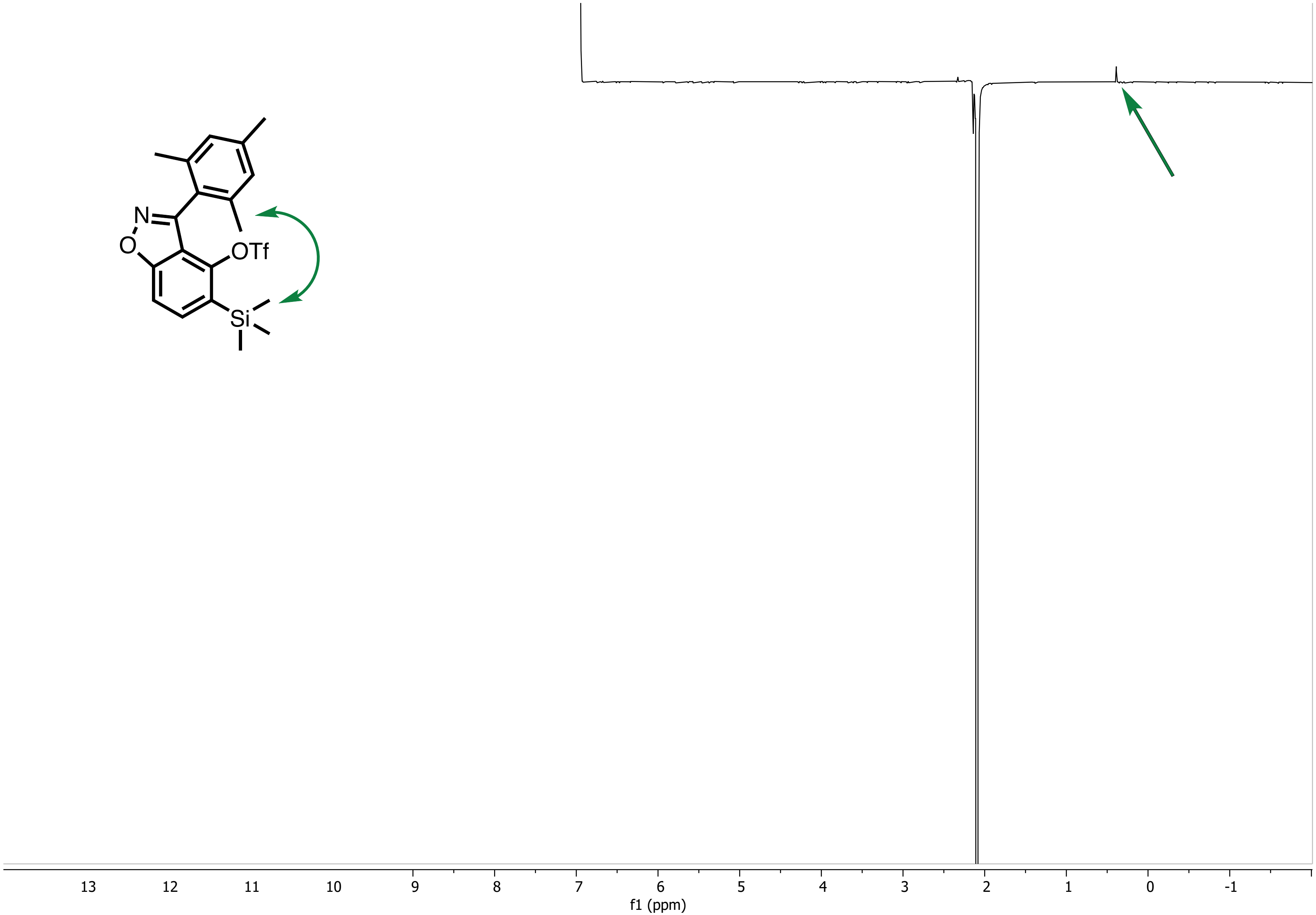

NOESY-1D (400 MHz, $CDCl_3$) spectrum of **11f** (satd. 2.11 ppm), establishing the regioisomeric identity.

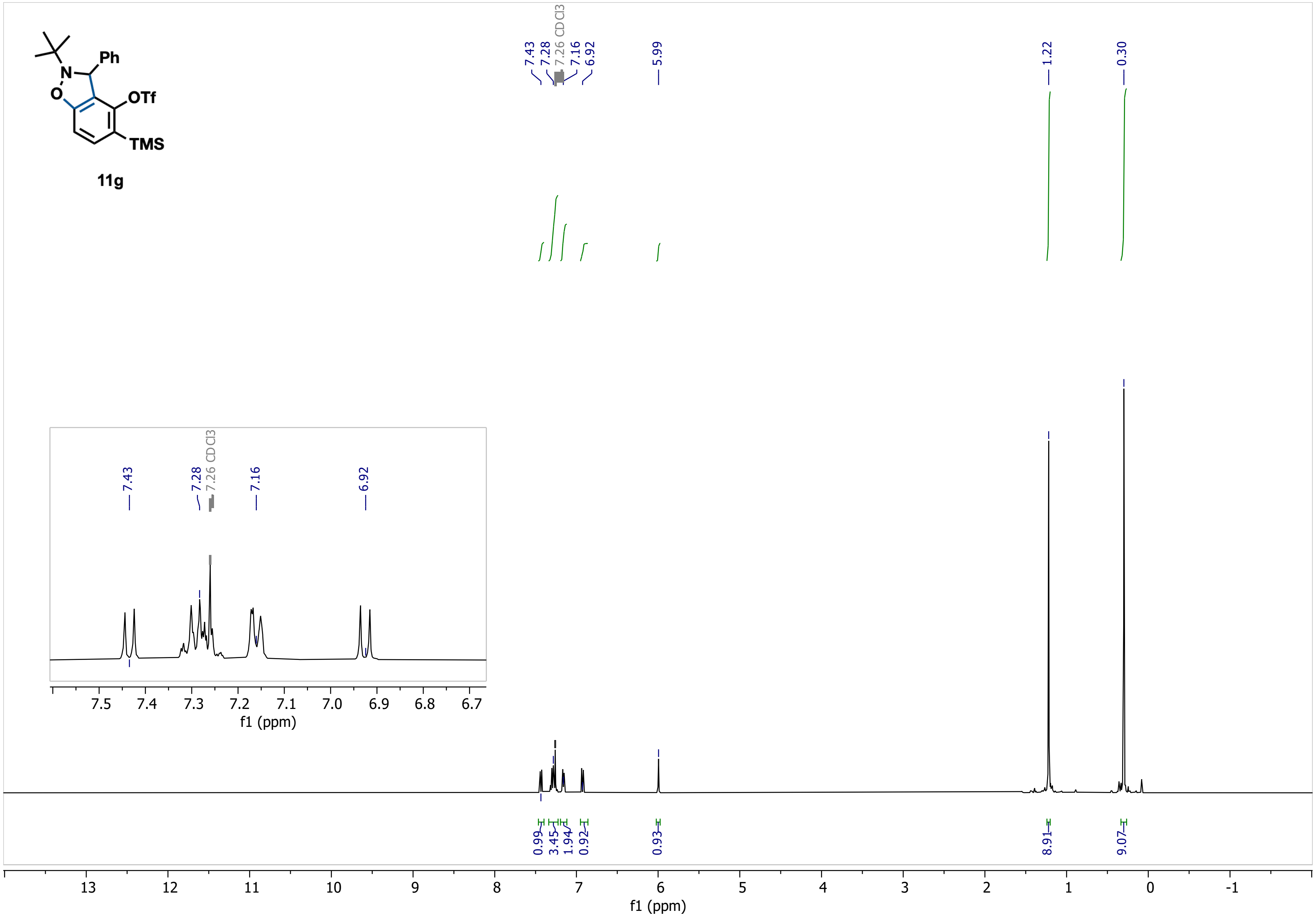

^{1}H NMR (400 MHz, $CDCl_3$) spectrum of compound **11g**.

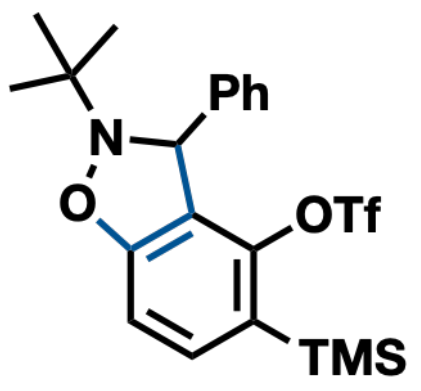

11g

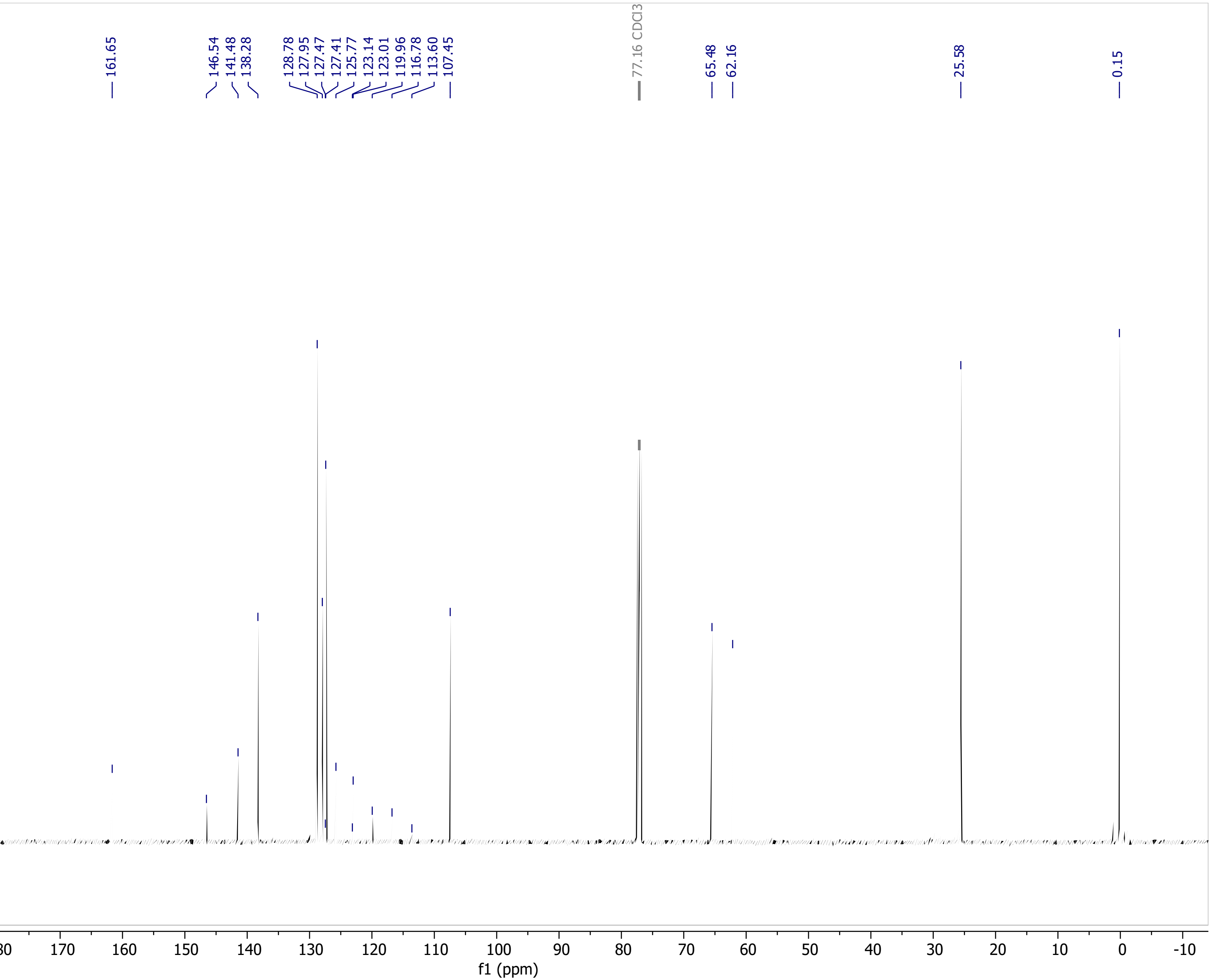

$^{13}C\{^{1}H\}$ NMR (101 MHz, $CDCl_3$) spectrum of compound **11g**.

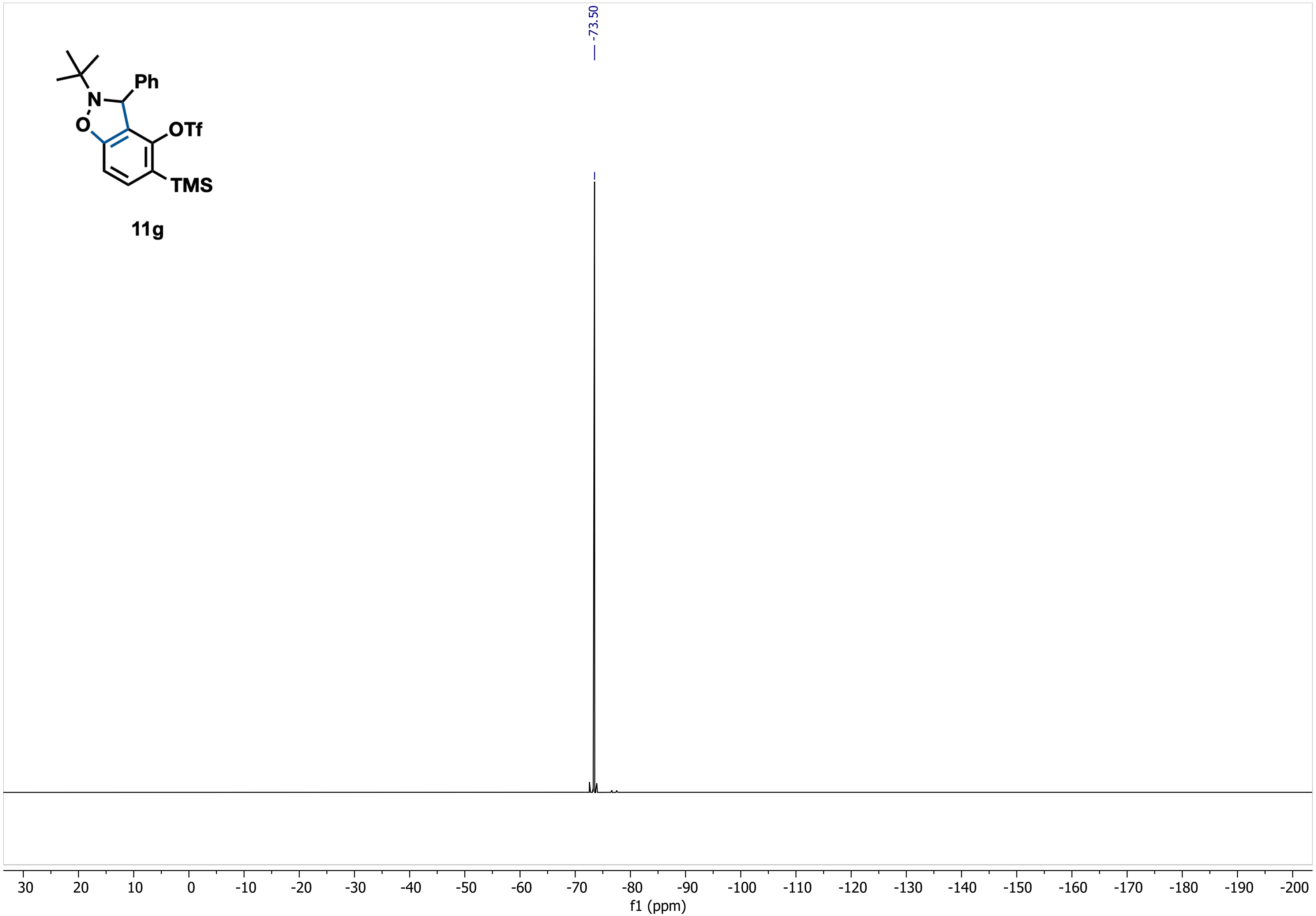

^{19}F NMR (376 MHz, $CDCl_3$) spectrum of compound **11g**.

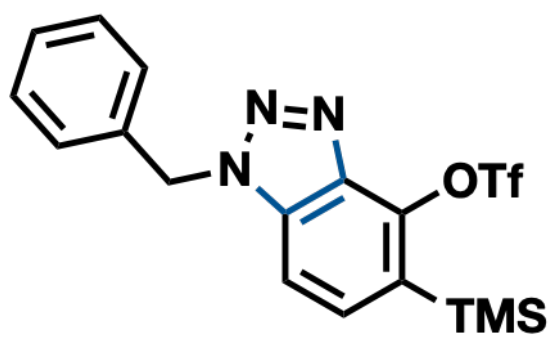

11h

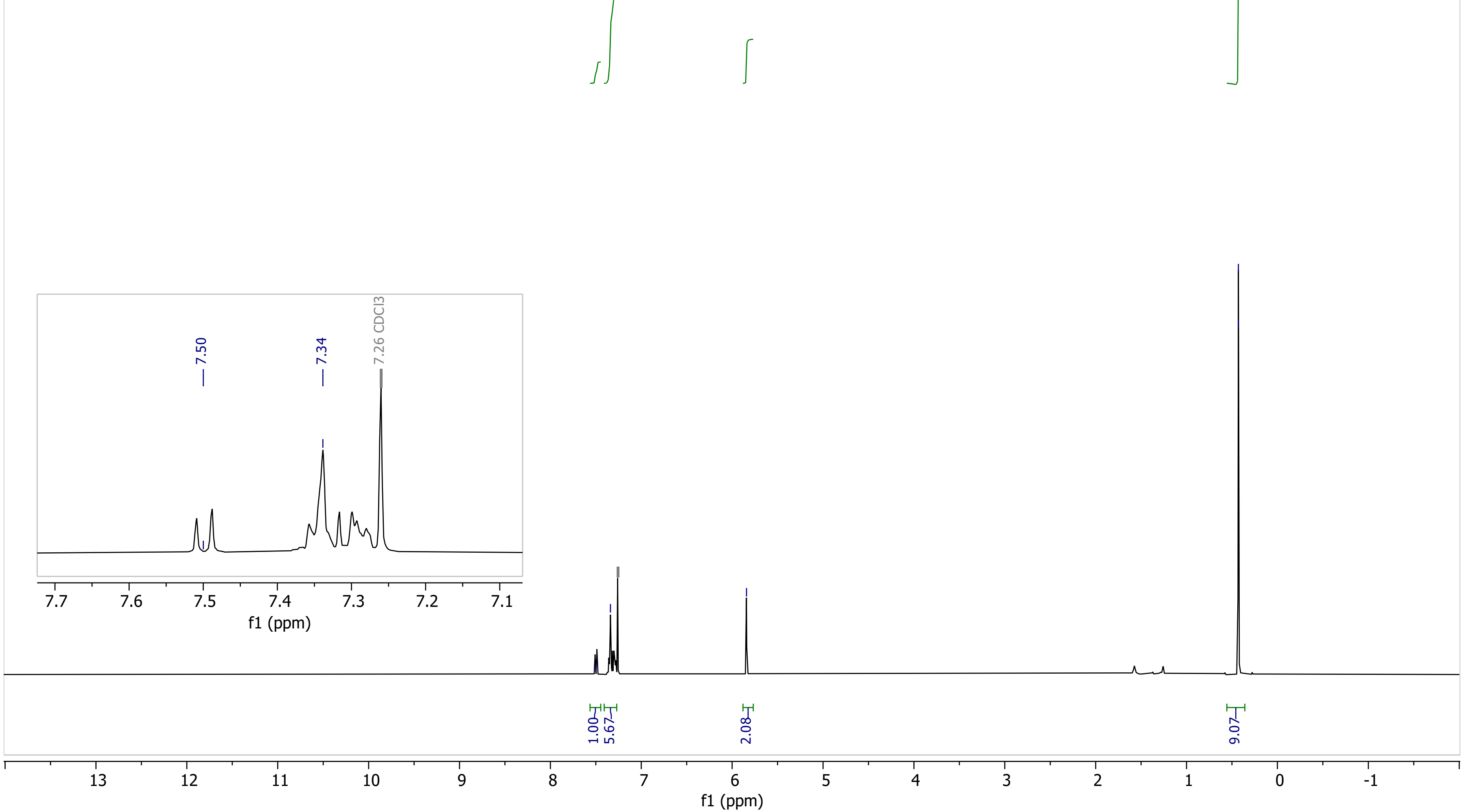

^{1}H NMR (400 MHz, $CDCl_3$) spectrum ($CDCl_3$) of compound **11h**.

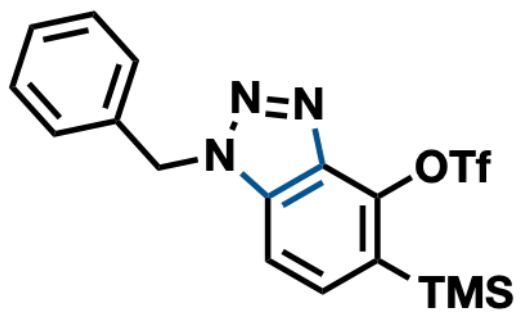

11h

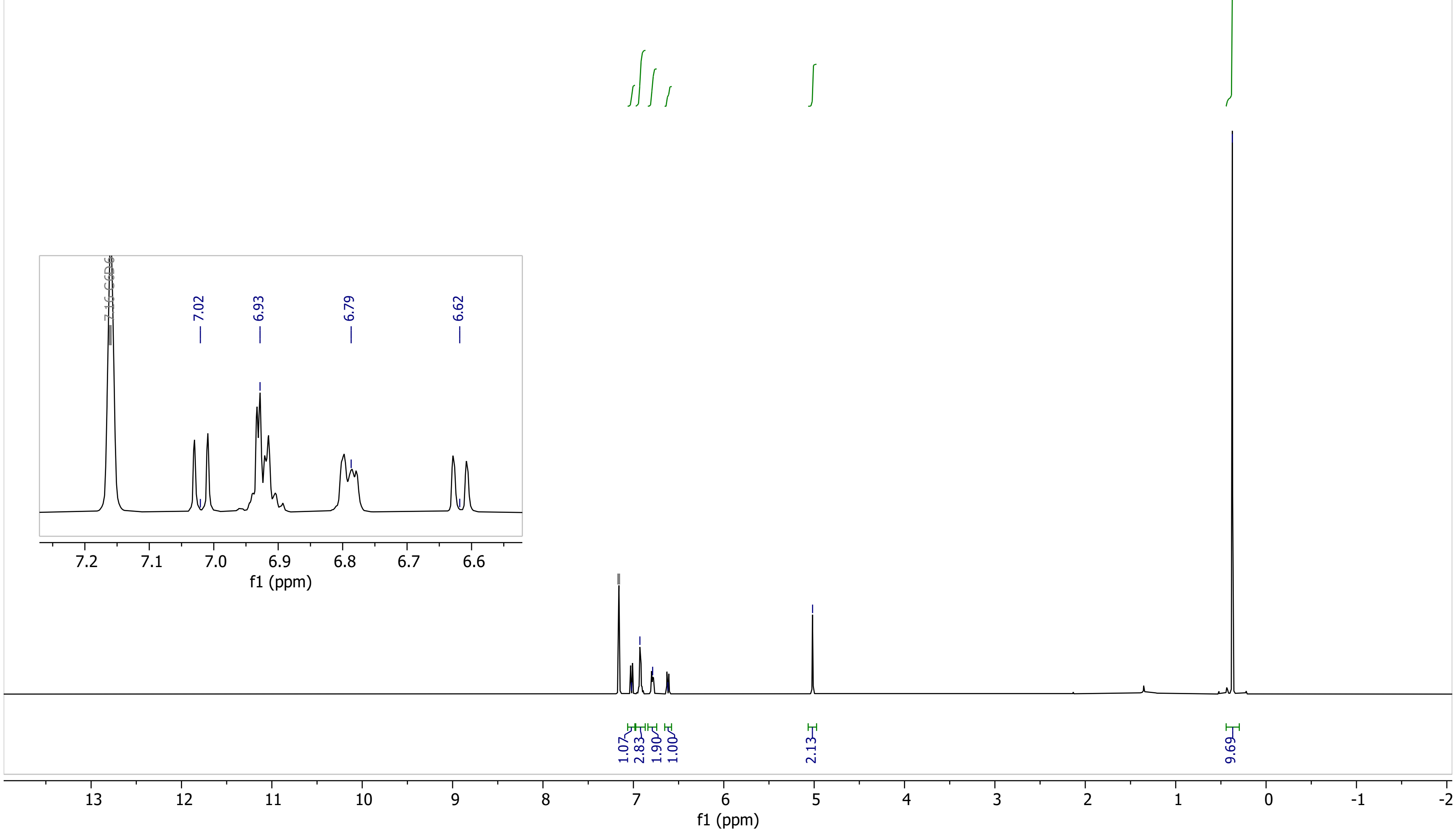

^{1}H NMR spectrum (C_6D_6) of compound **11h**.

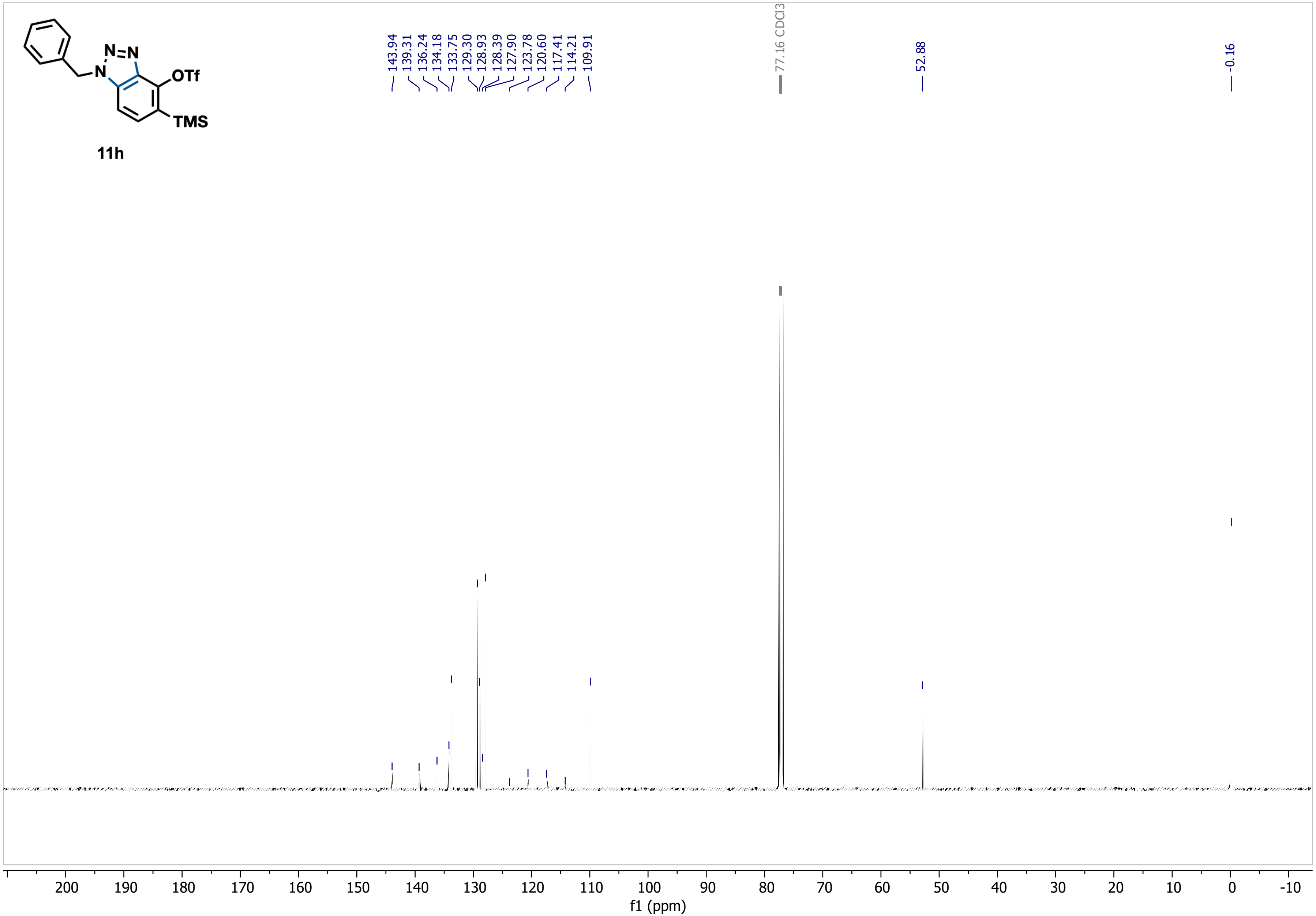

11h

$^{13}C\{^{1}H\}$ NMR (101 MHz, $CDCl_3$) spectrum of compound **11h**.

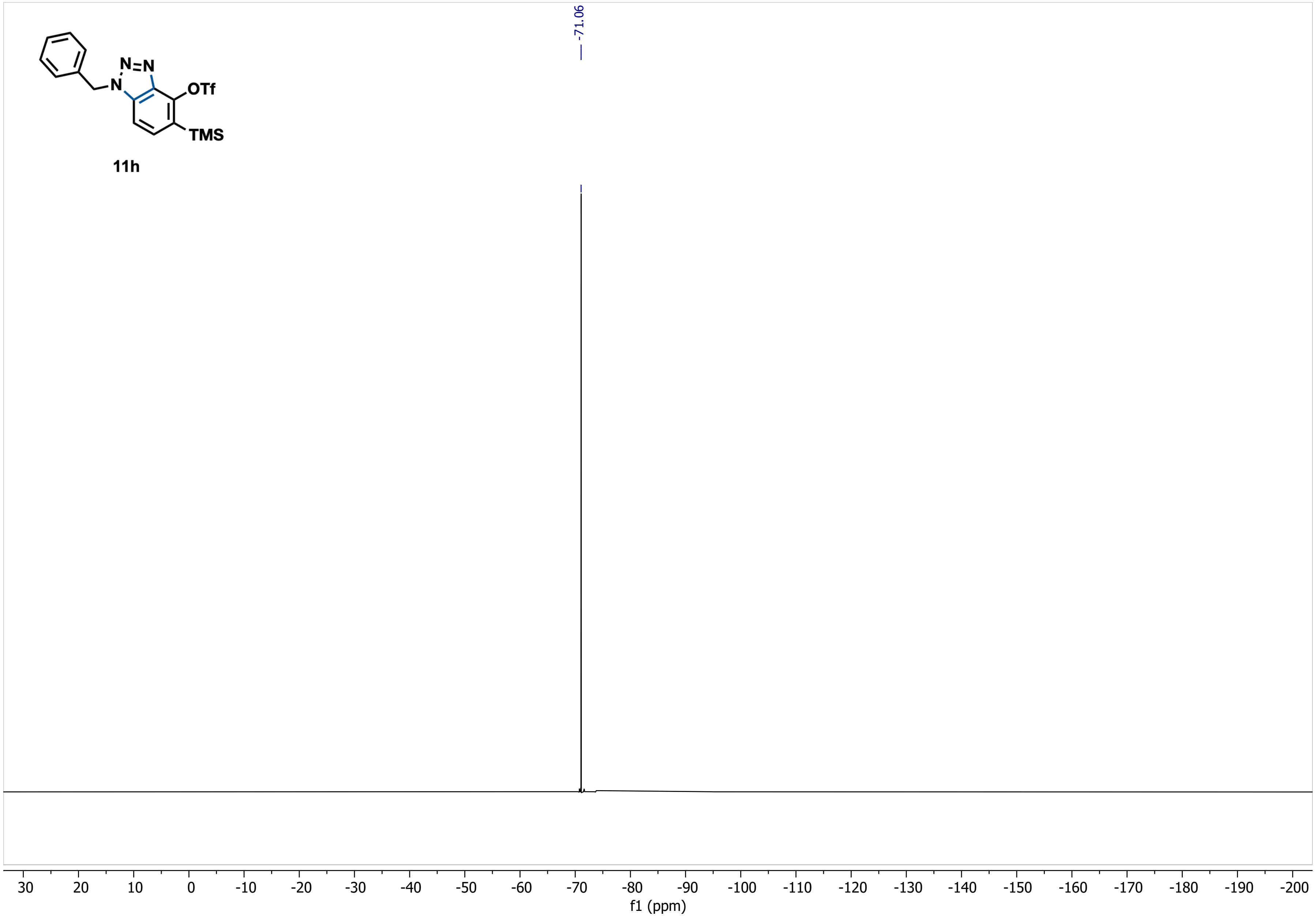

^{19}F NMR (376 MHz, $CDCl_3$) spectrum of compound **11h**.

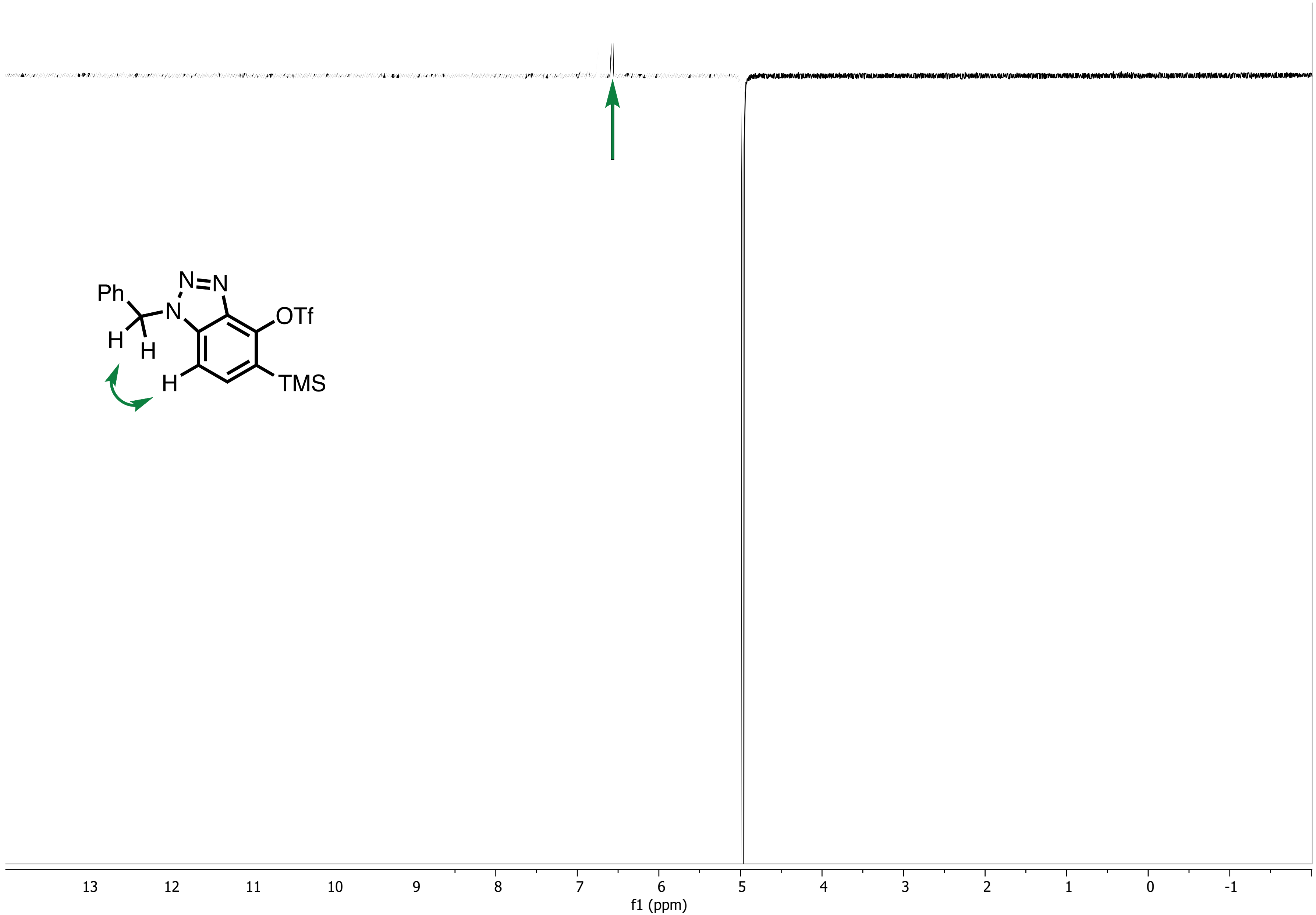

NOESY-1D (400 MHz, C_6D_6) spectrum of **11h** (satd. 4.97 ppm), establishing the regioisomeric identity.

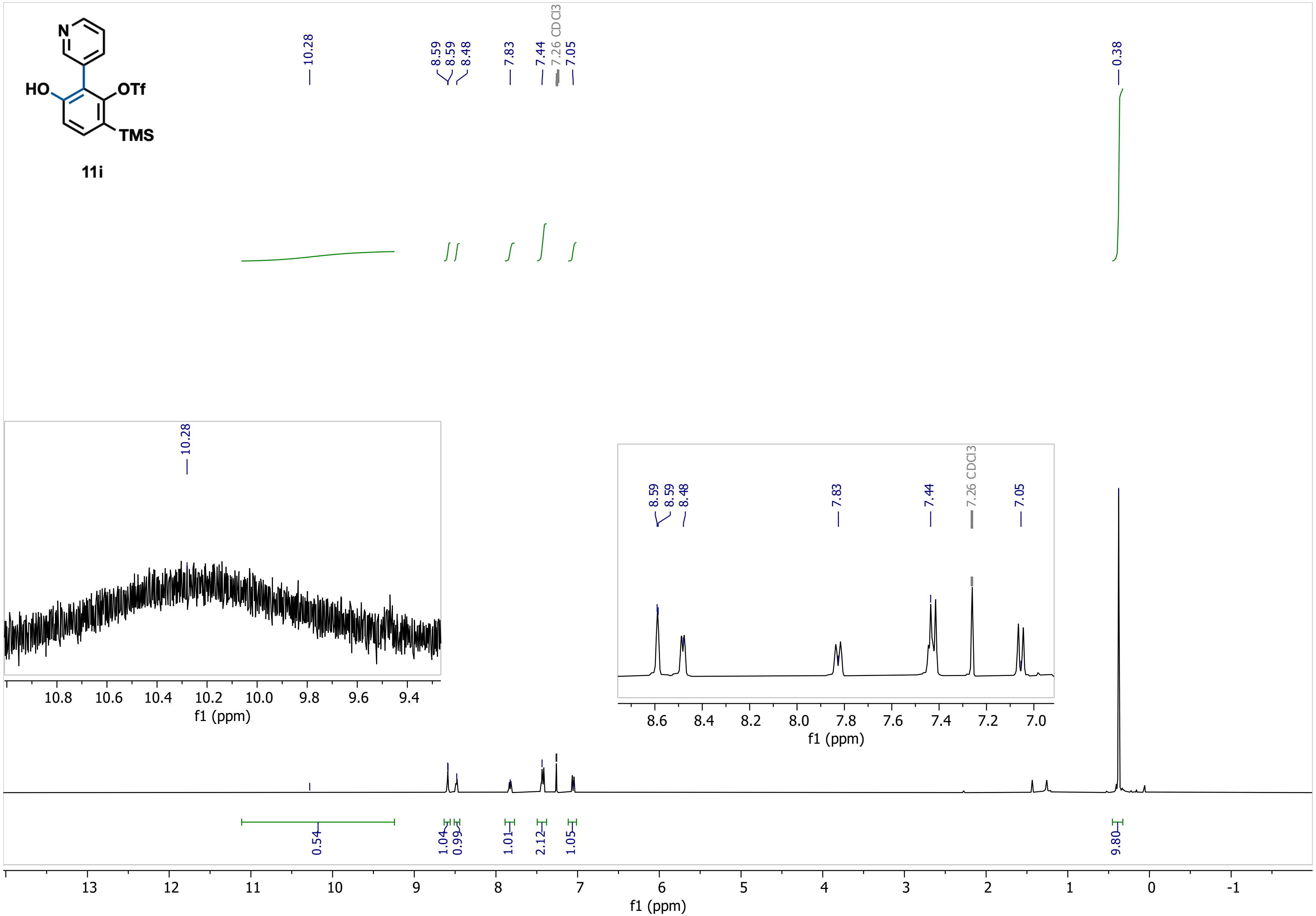

^{1}H NMR (400 MHz, $CDCl_3$) spectrum of compound **11i**.

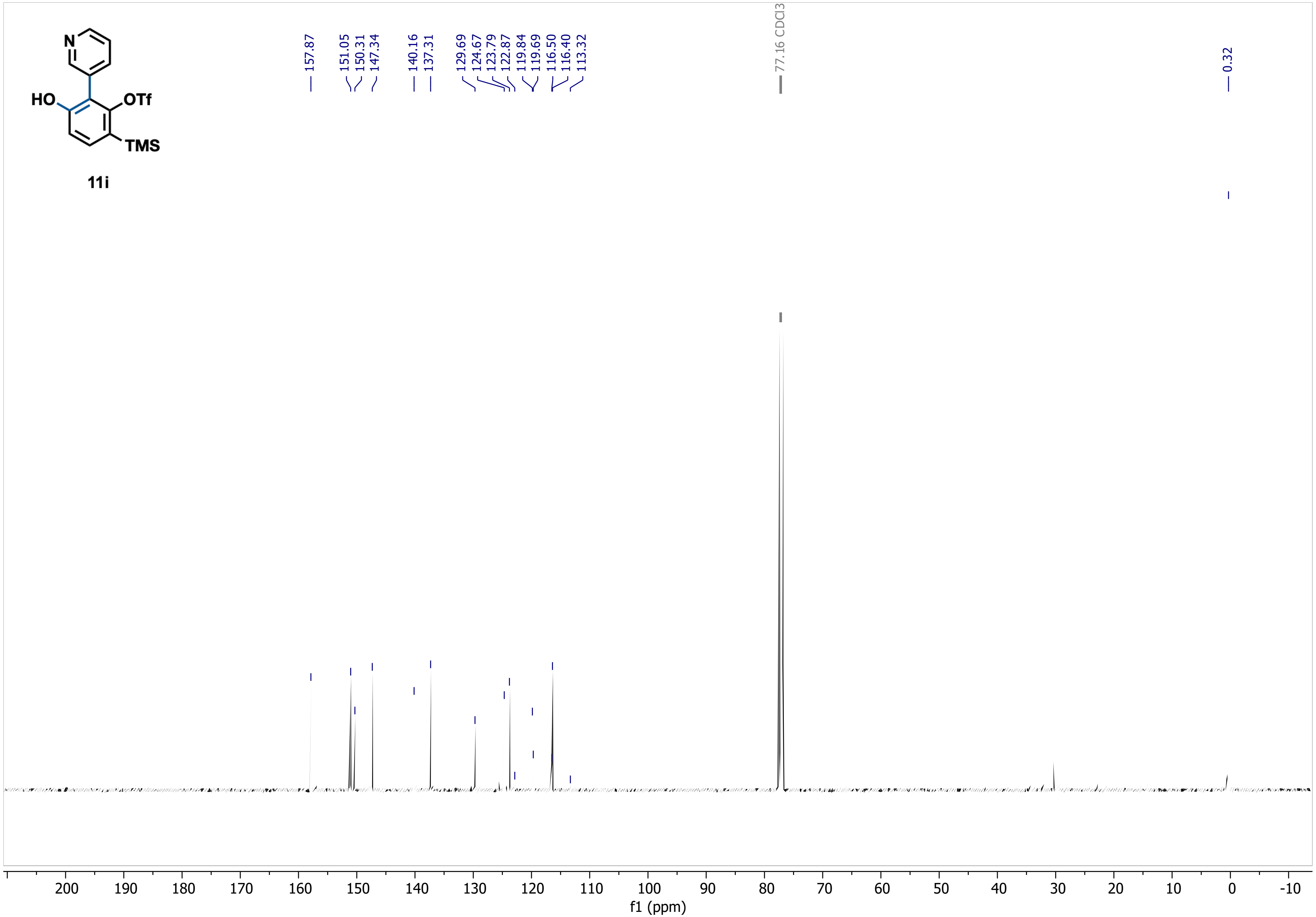

$^{13}C\{^{1}H\}$ NMR (101 MHz, $CDCl_3$) spectrum of compound **11i.**

NMR spectra – Scheme 3

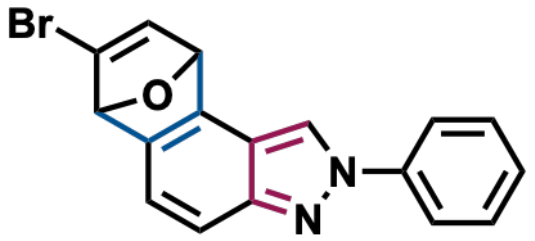

13a

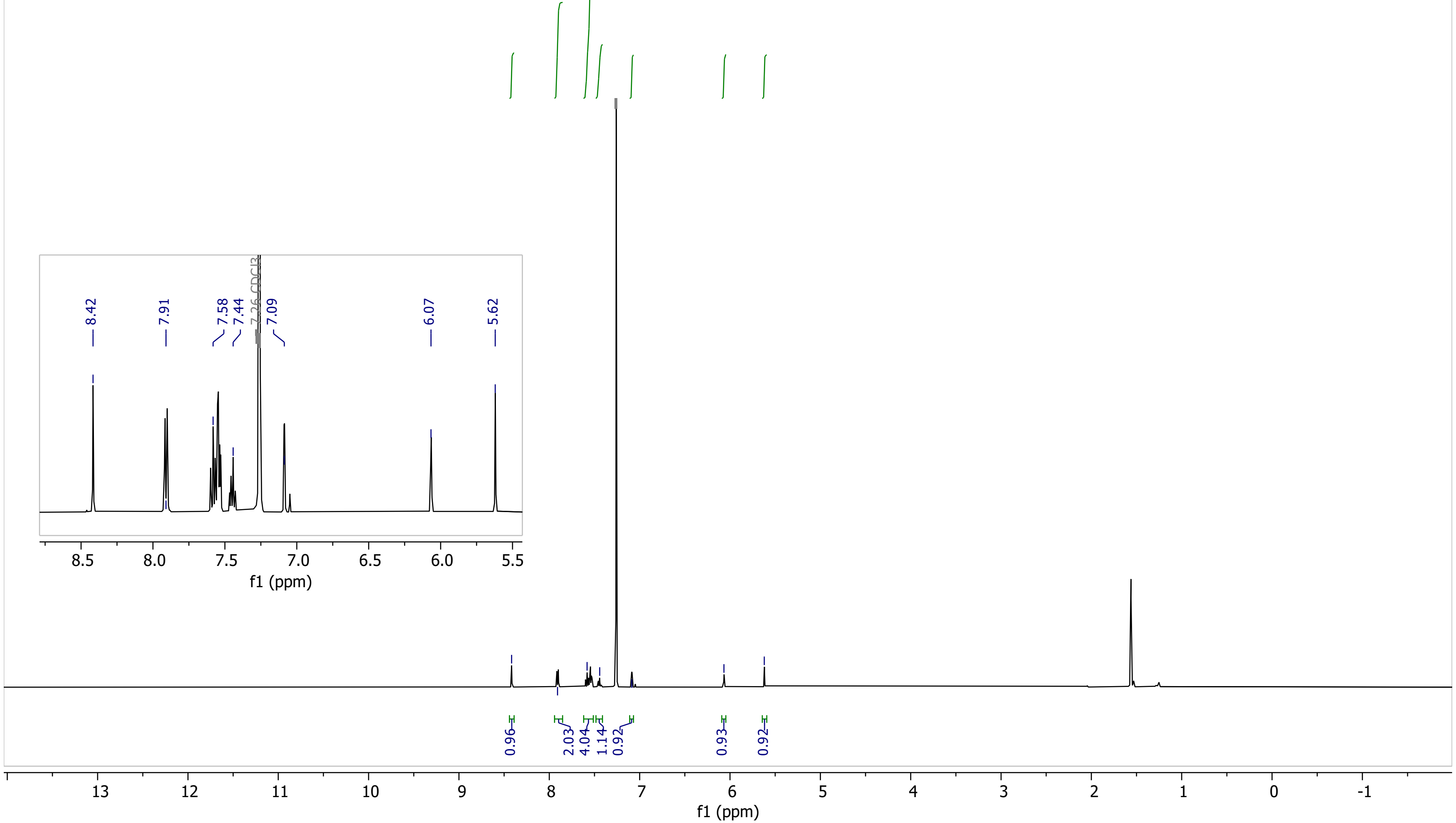

^{1}H NMR (500 MHz, $CDCl_3$) spectrum of compound **13a**.

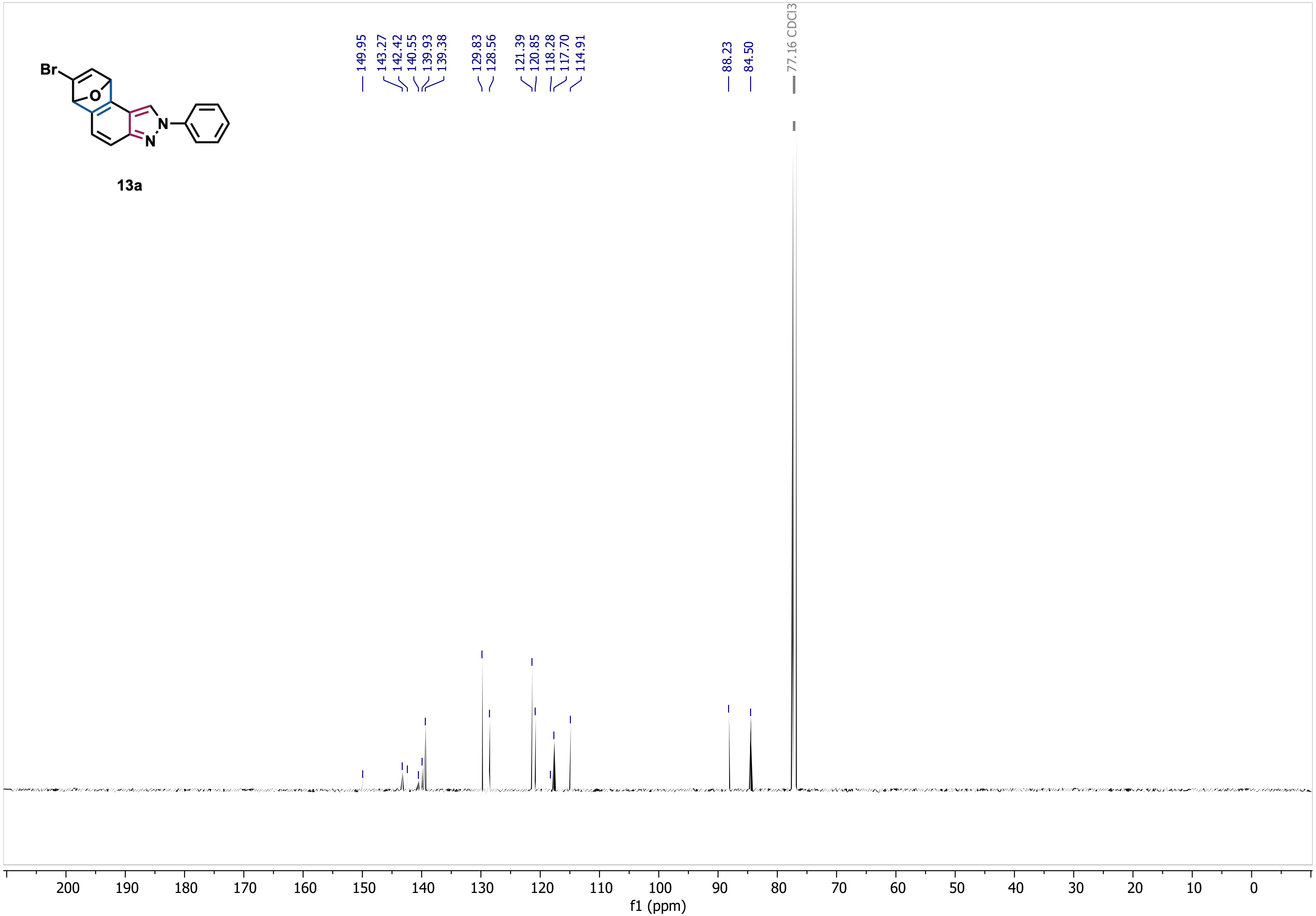

^{3}C{^{1}H} NMR (126 MHz, $CDCl_3$) spectrum of compound **13a**.

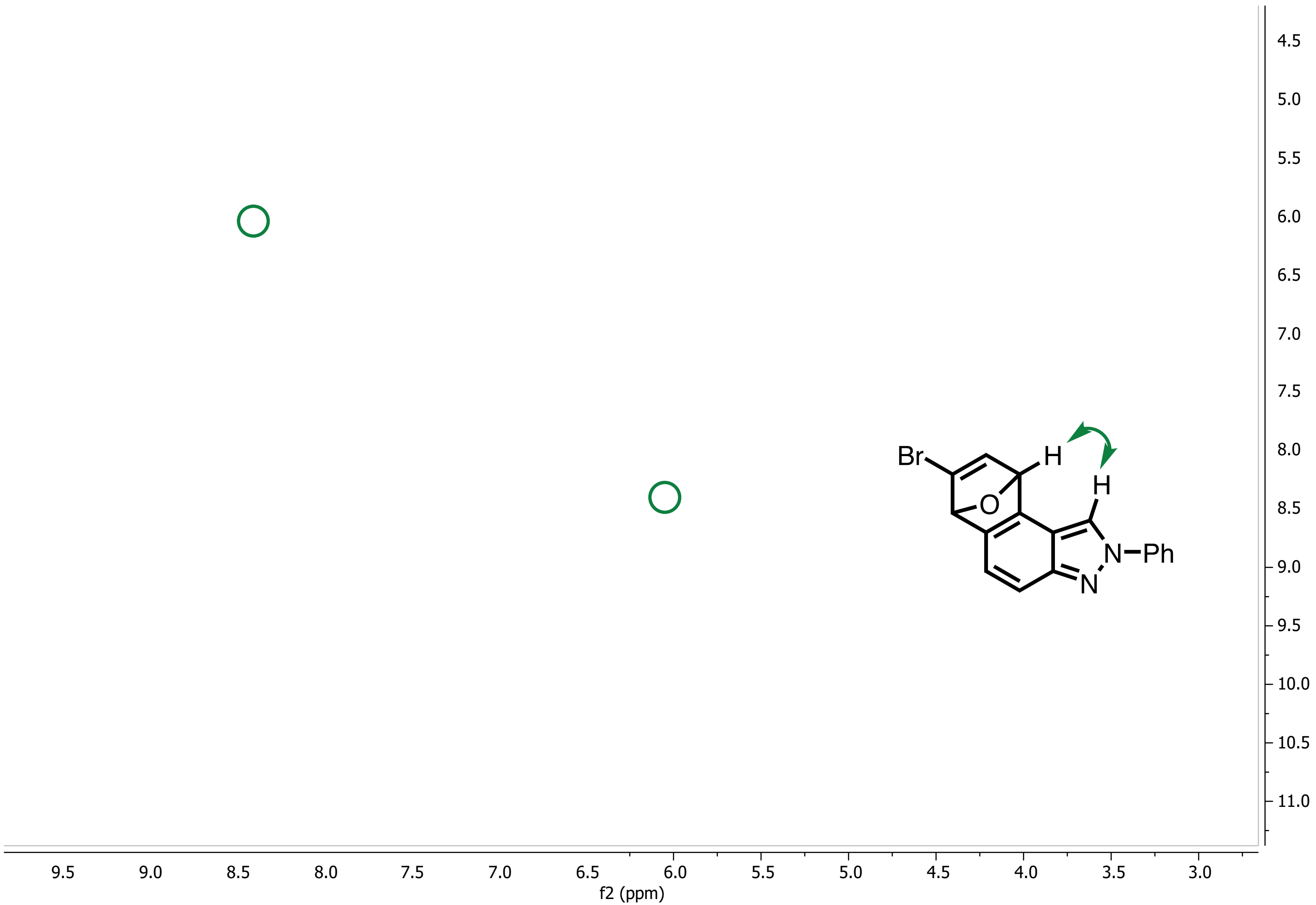

NOESY (500 MHz, $CDCl_3$) spectrum of compound **13a**, establishing the regioisomeric identity.

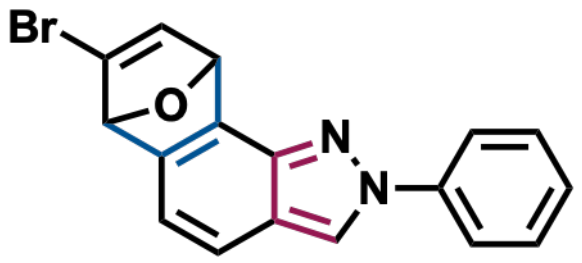

13a'

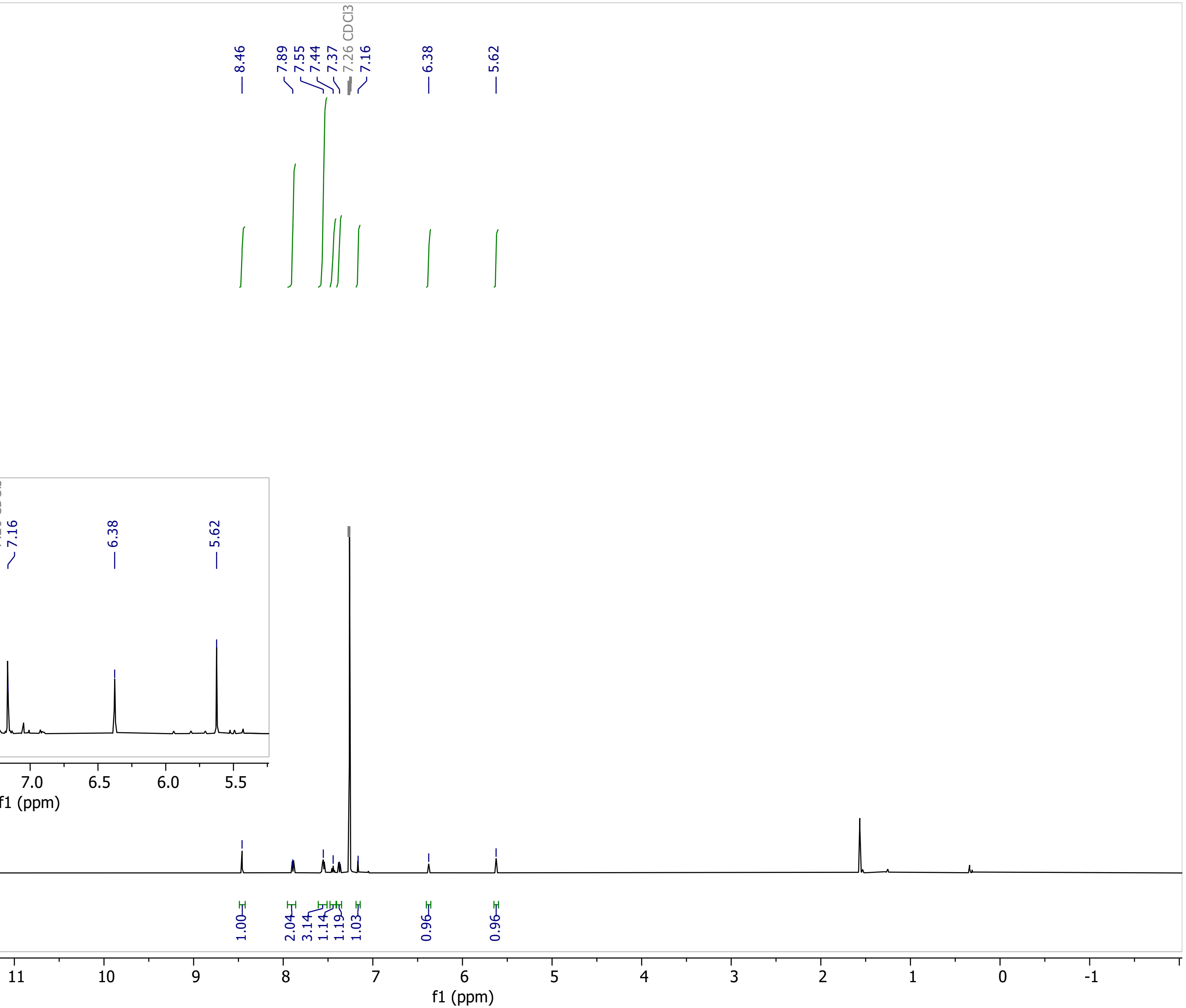

^{1}H NMR (500 MHz, $CDCl_3$) spectrum of compound **13a'**.

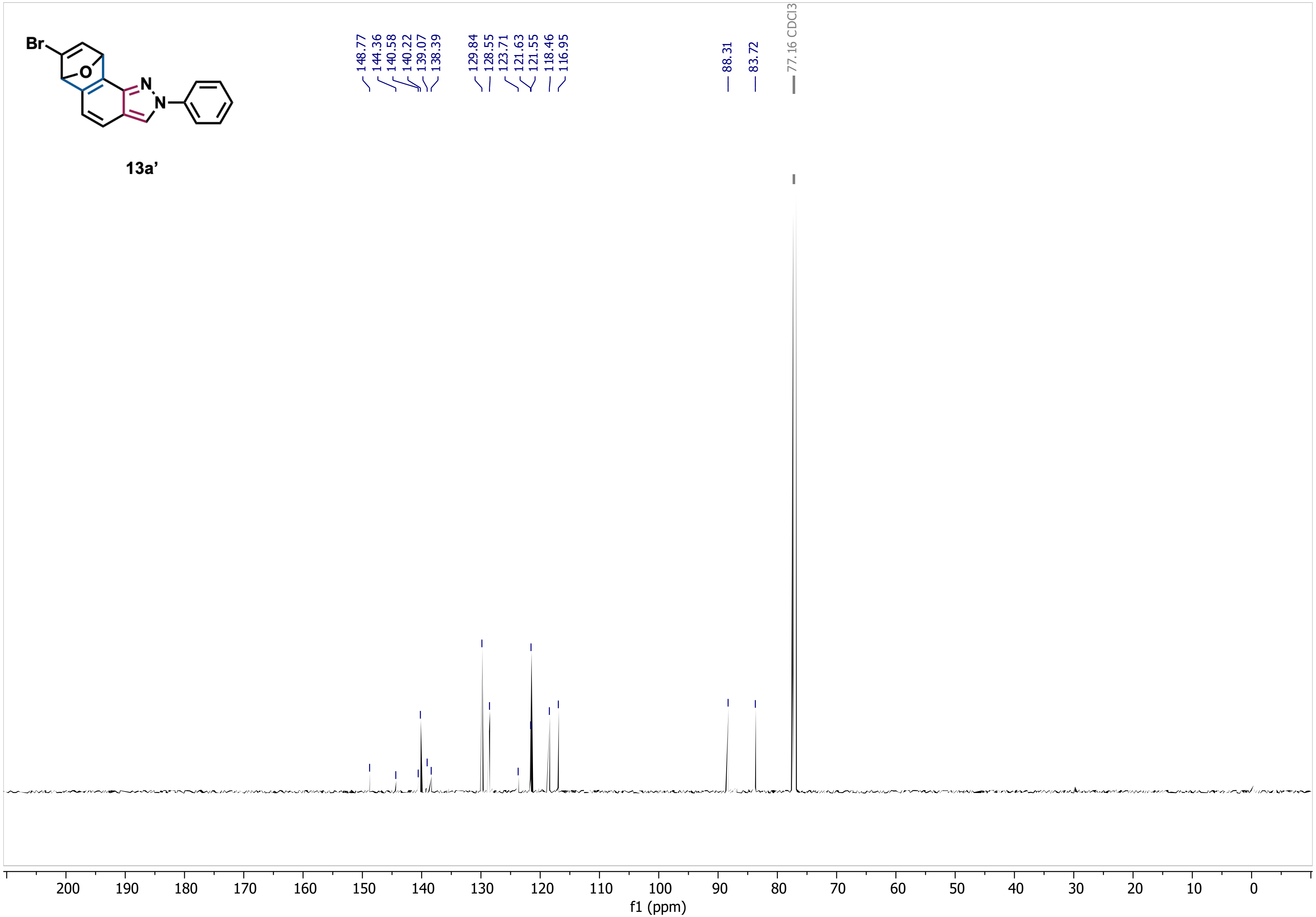

$^{3}C\{^{1}H\}$ NMR (126 MHz, $CDCl_3$) spectrum of compound **13a’**.

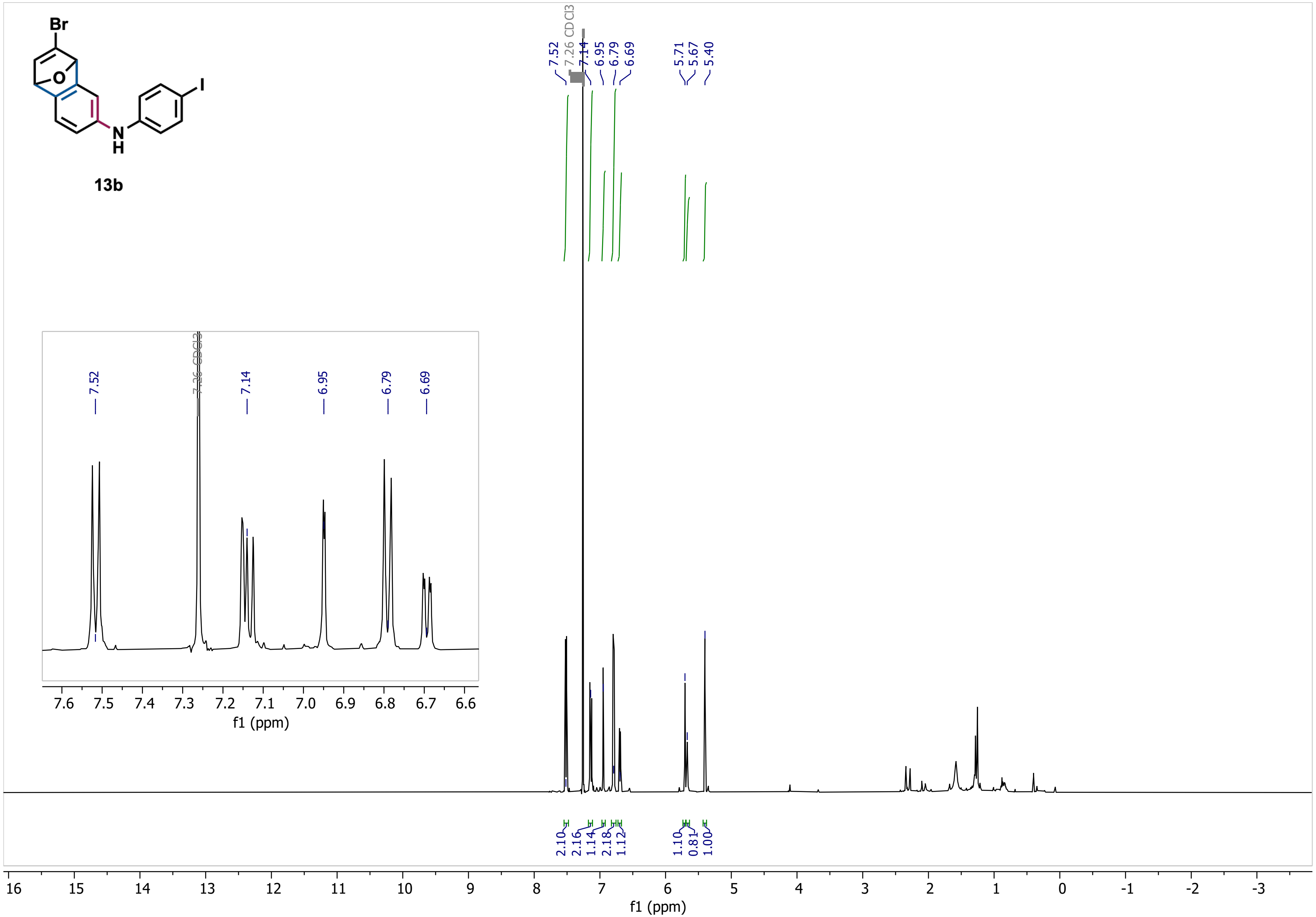

^{1}H NMR (500 MHz, $CDCl_3$) spectrum of compound **13b**.

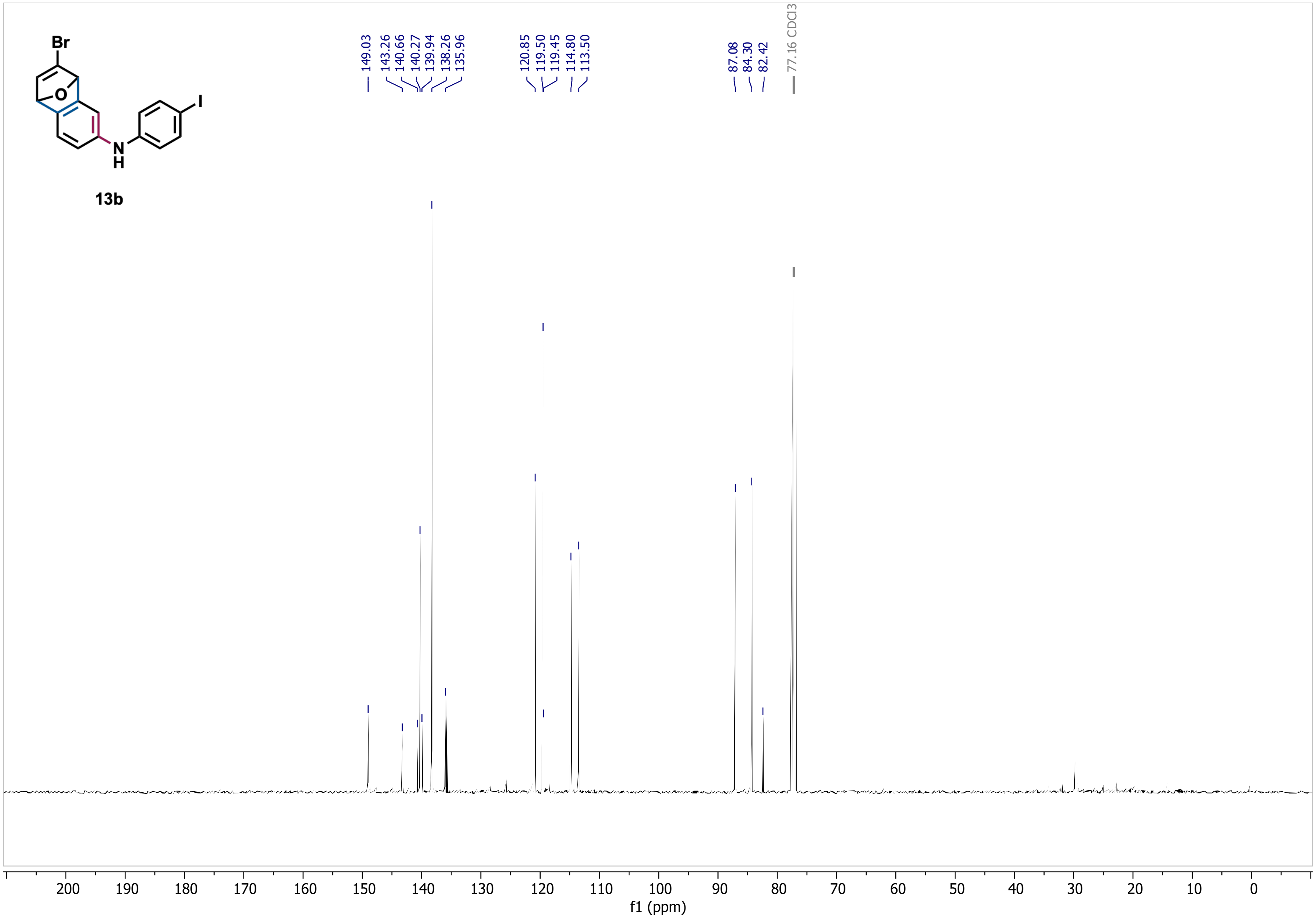

^{3}C{^{1}H} NMR (126 MHz, $CDCl_3$) spectrum of compound **13b**.

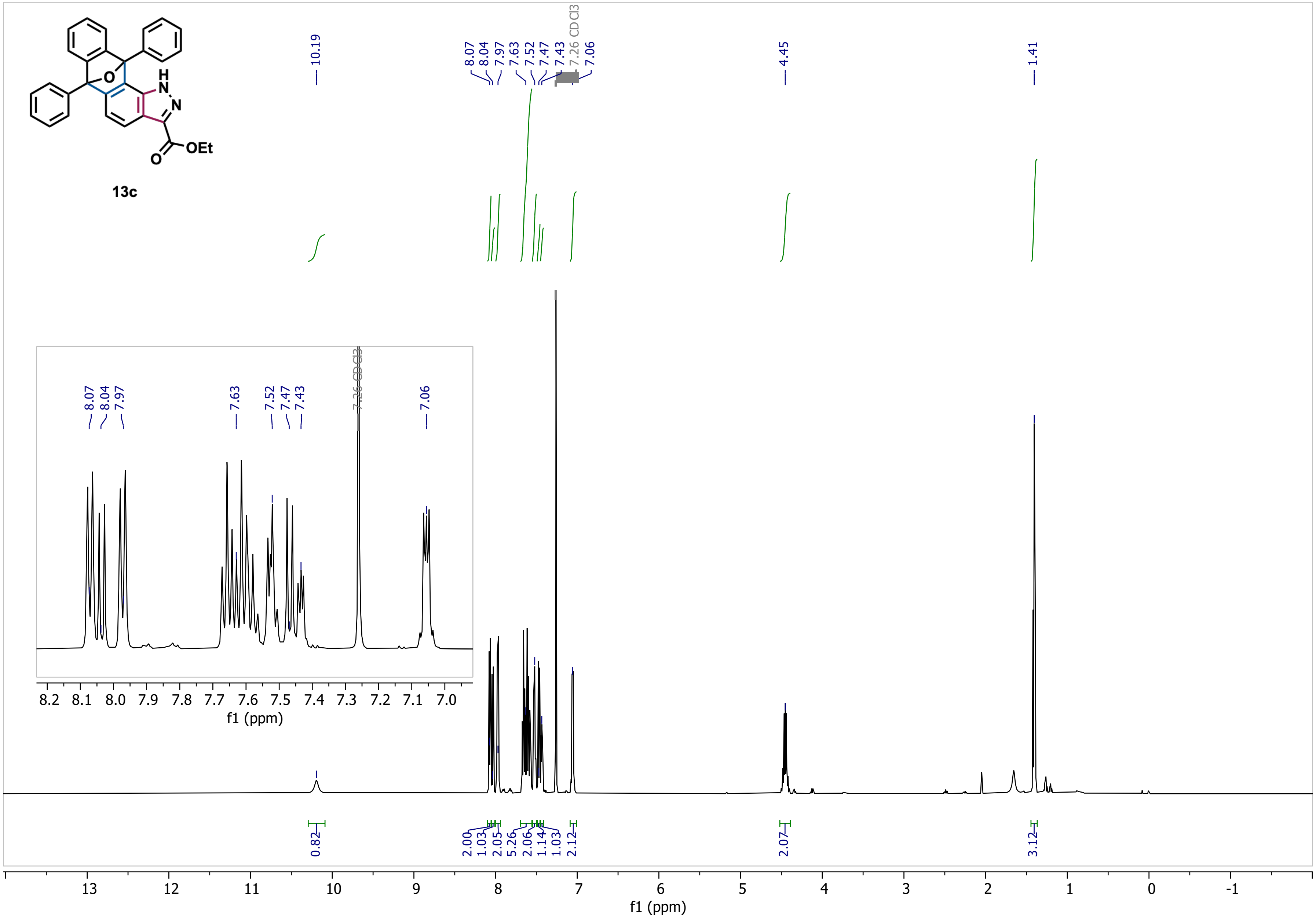

^{1}H NMR (500 MHz, $CDCl_3$) spectrum of compound **13c**.

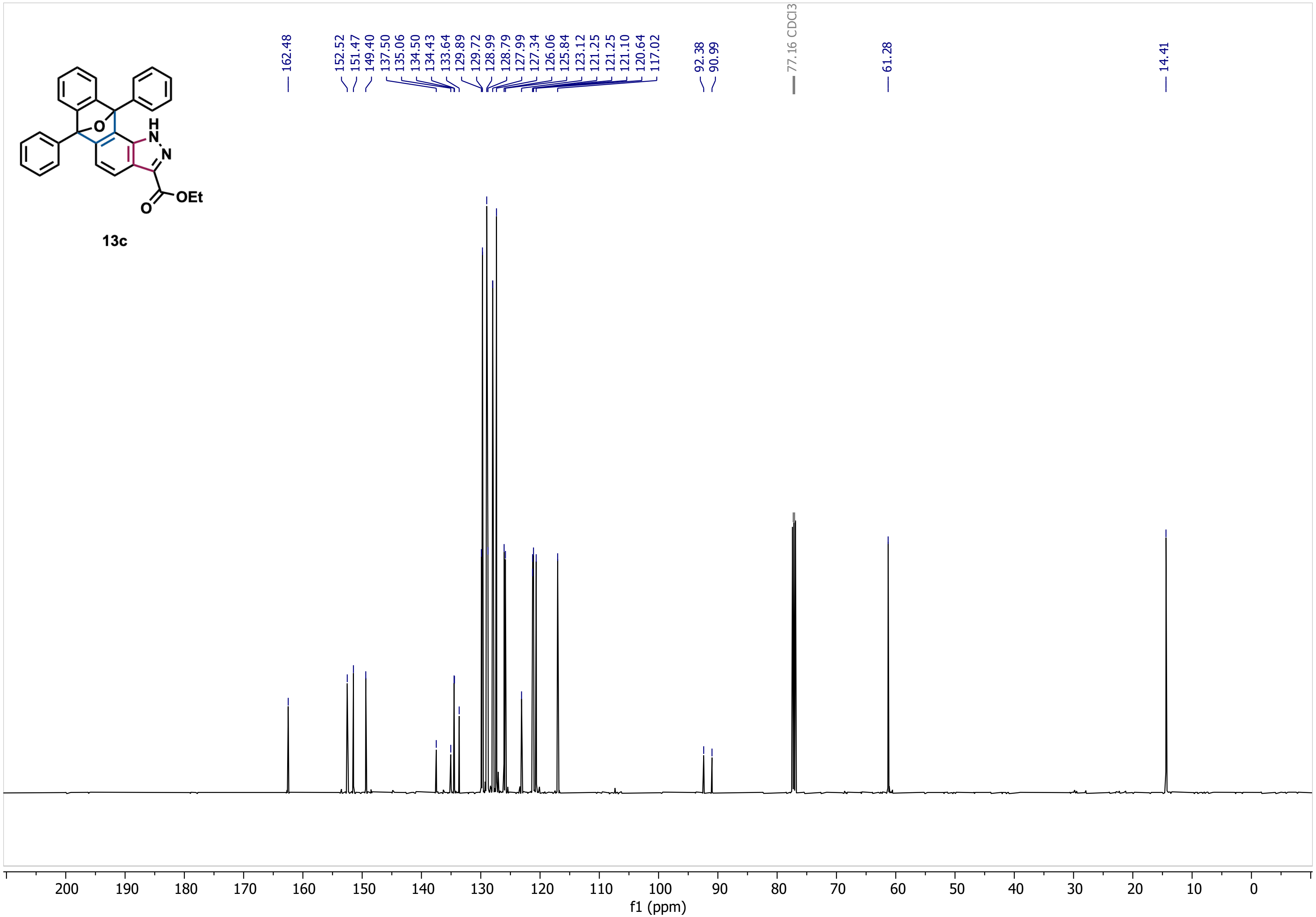

^{3}C{^{1}H} NMR (126 MHz, $CDCl_3$) spectrum of compound **13c**.

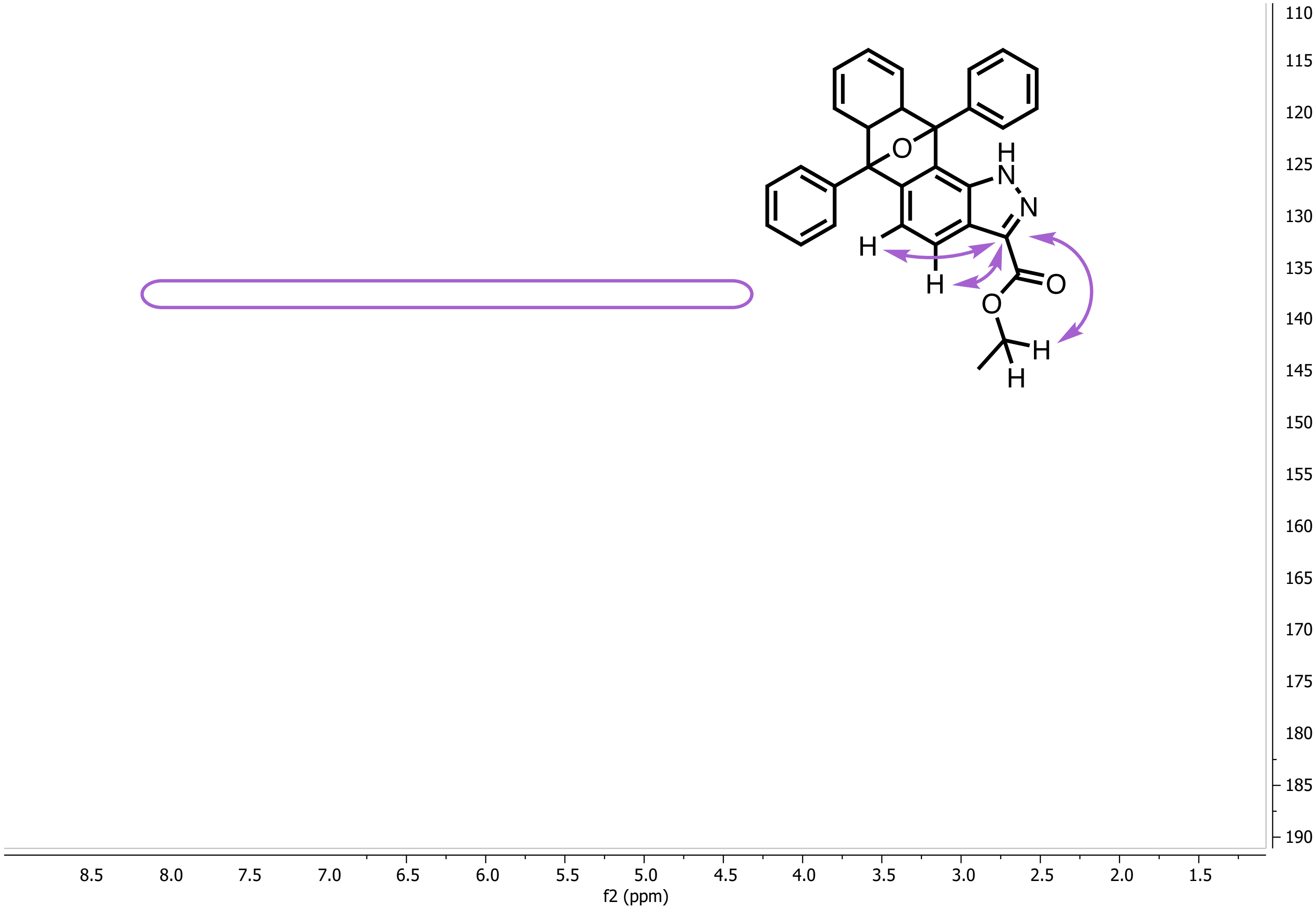

HMBC (J_{XCH} = 3 Hz) spectrum of **13c**, with correlations that establish the regioisomeric identity.

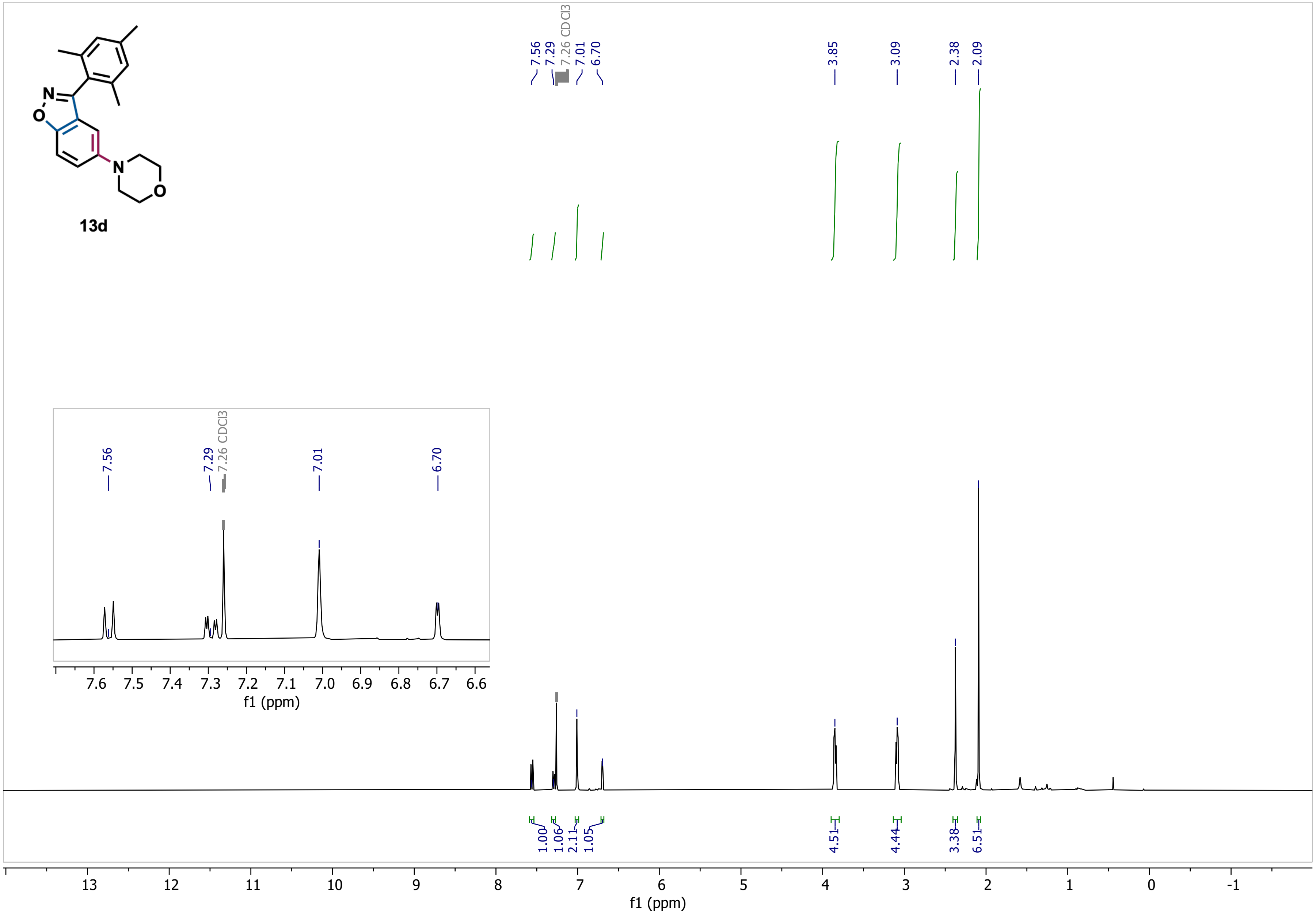

^{1}H NMR (400 MHz, $CDCl_3$) spectrum of compound **13d**.

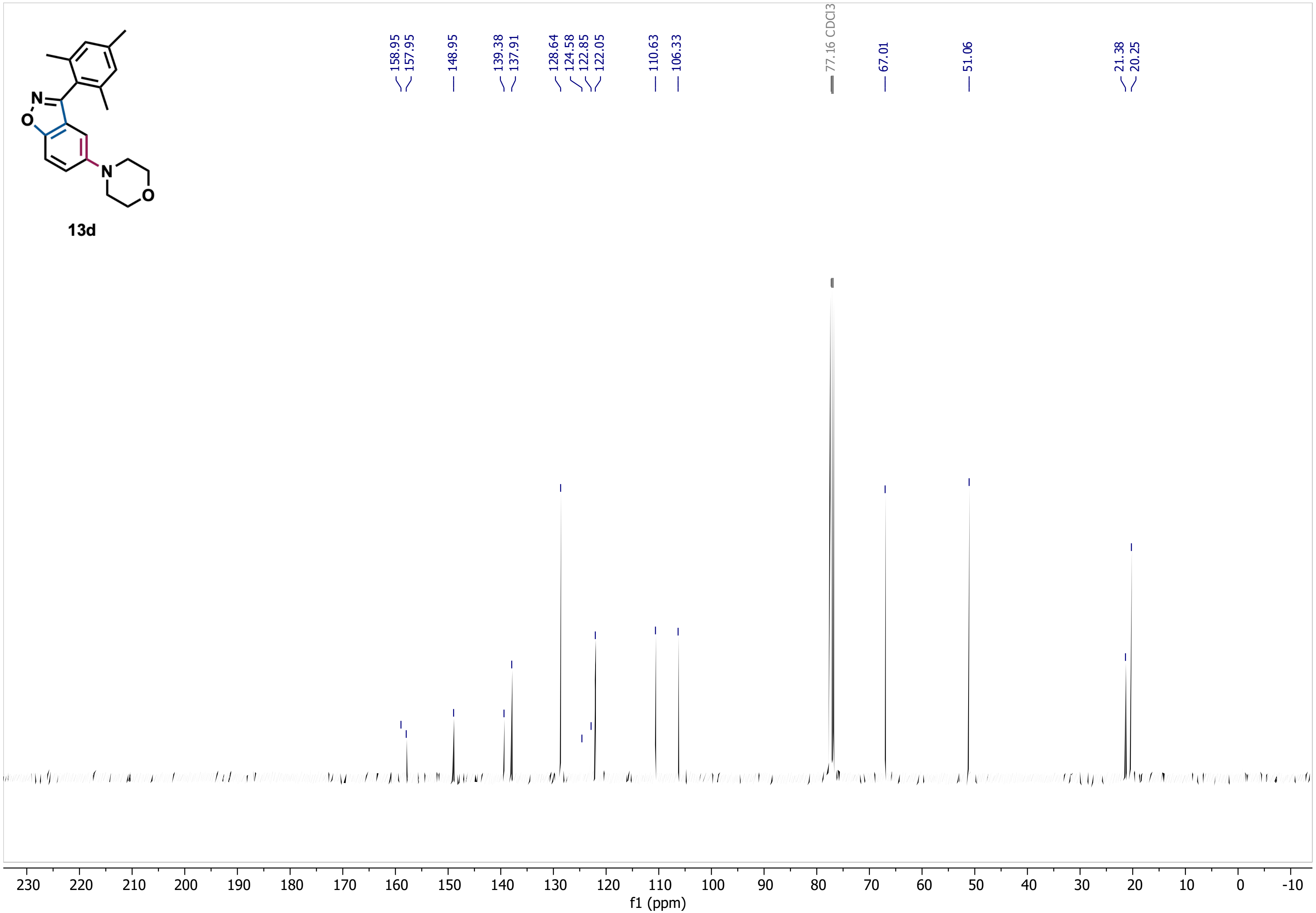

^{3}C{^{1}H} NMR (126 MHz, $CDCl_3$) spectrum of compound **13d**.

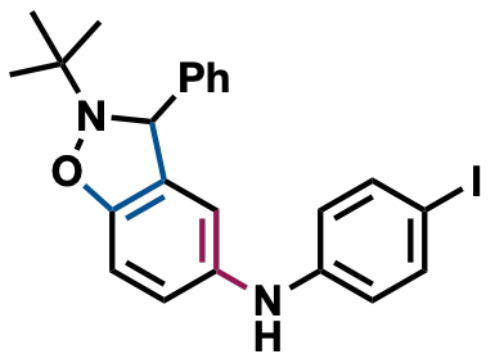

13e

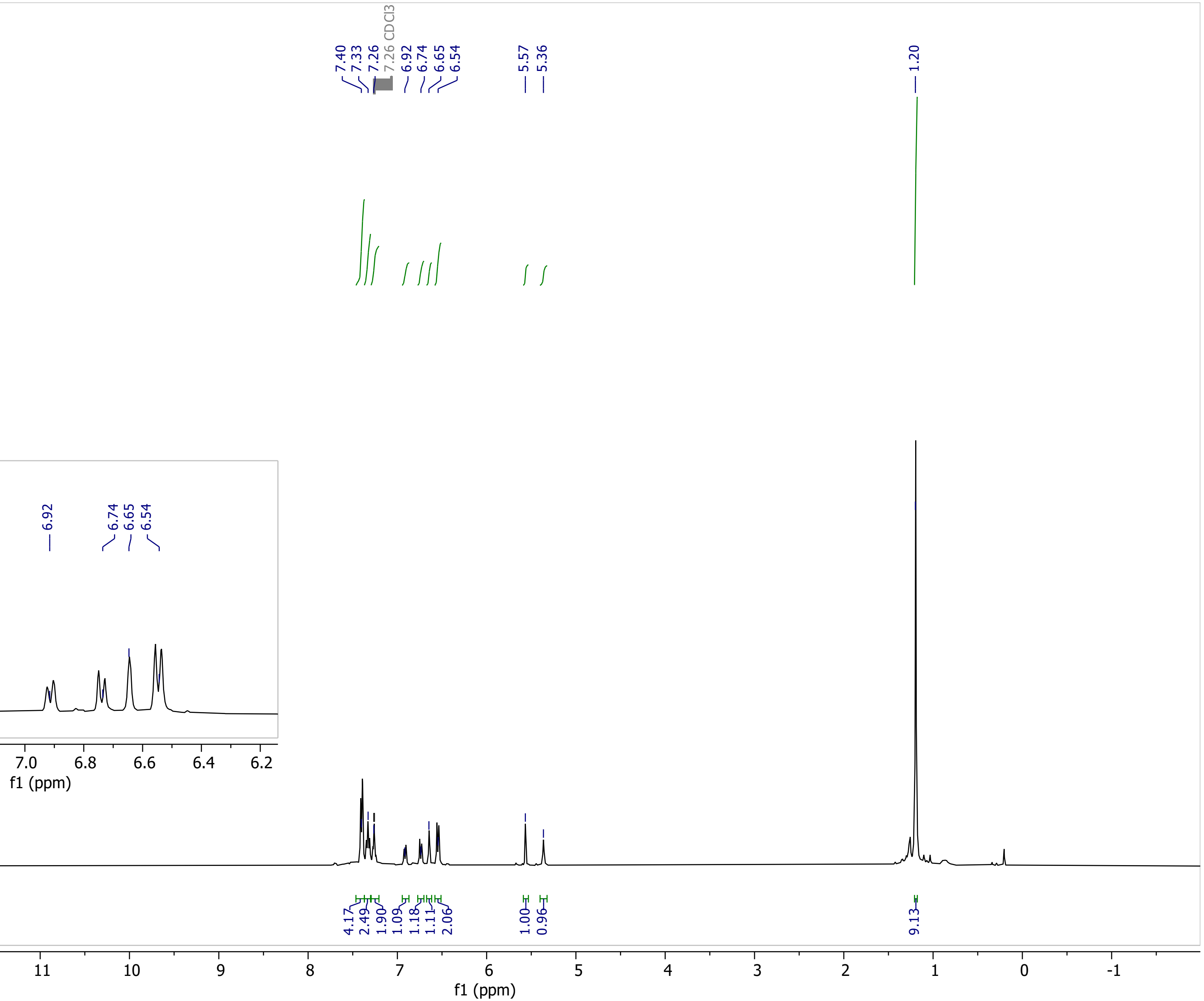

7.40
7.33
7.26
7.26 CDCl3
6.92
6.74
6.65
6.54
5.57
5.36
1.20
4.17
2.49
1.90
1.09
1.18
1.11
2.06
1.00
0.96
9.13
13
12
11
10
9
8
7
6
5
4
3
2
1
0
-1
f1 (ppm)
7.6
7.4
7.2
7.0
6.8
6.6
6.4
6.2

^{1}H NMR (400 MHz, $CDCl_3$) spectrum of compound **13e**.

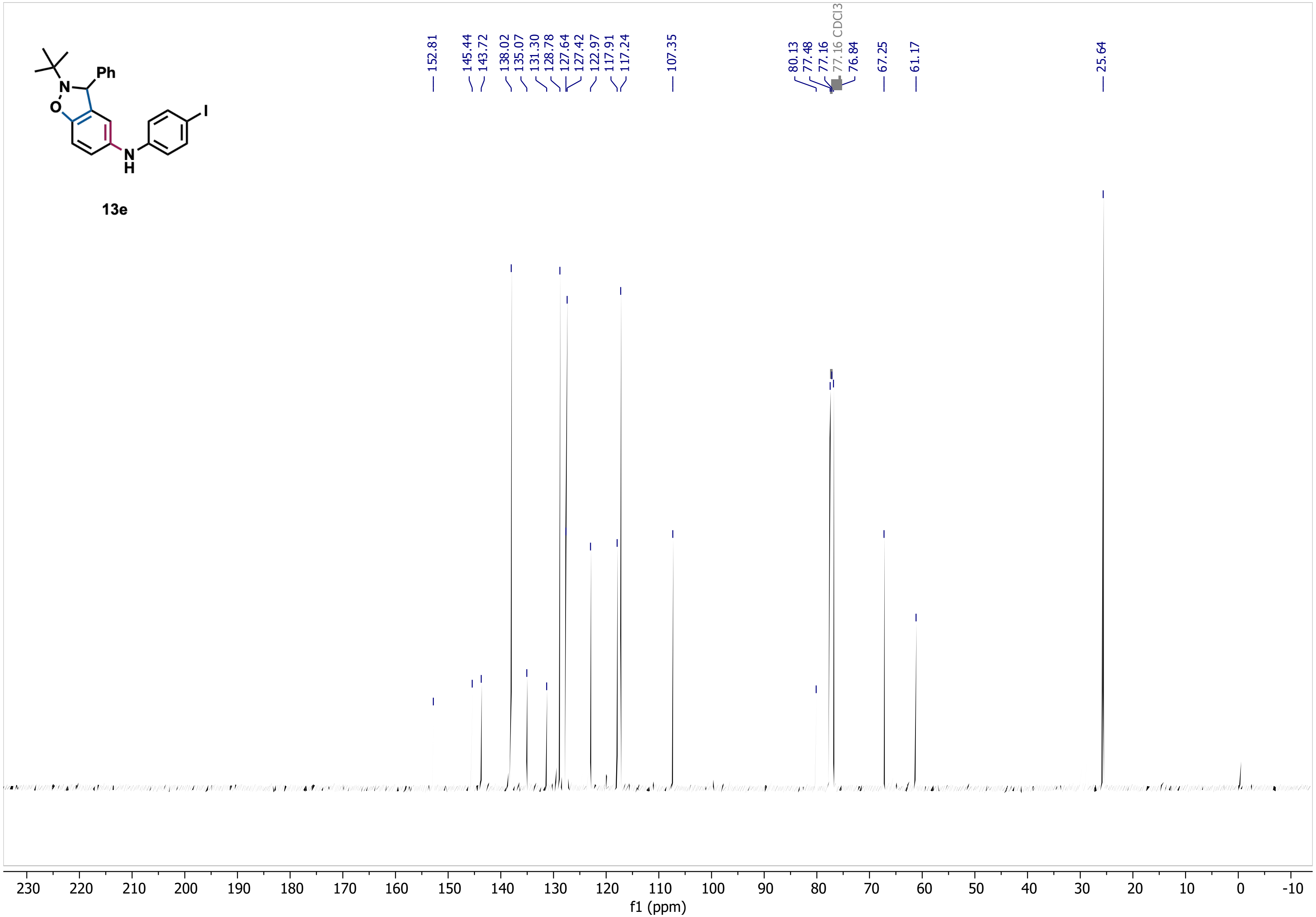

^{3}C{^{1}H} NMR (126 MHz, $CDCl_3$) spectrum of compound **13e**.

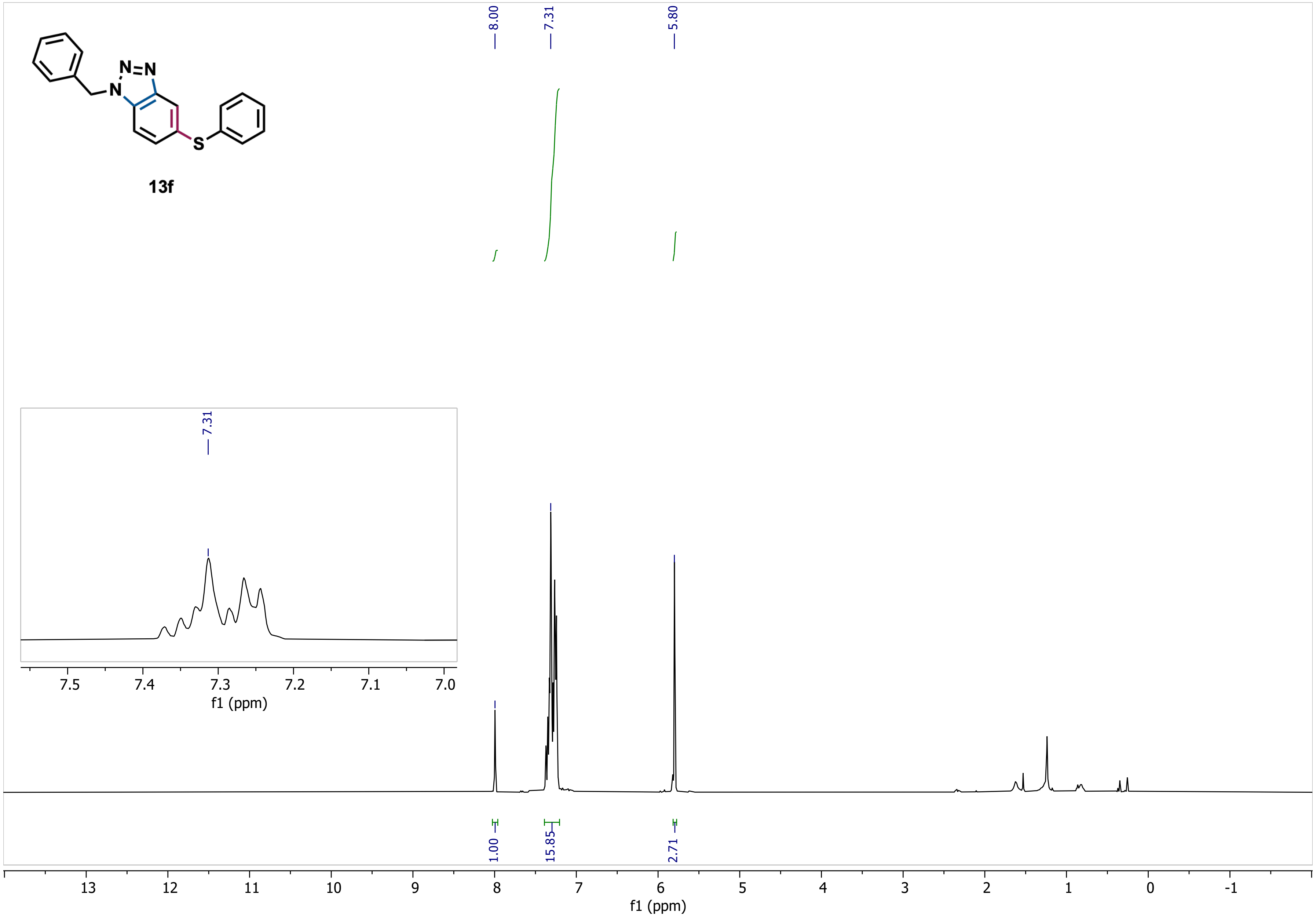

^{1}H NMR (400 MHz, C_6D_6) spectrum of compound **13f**.

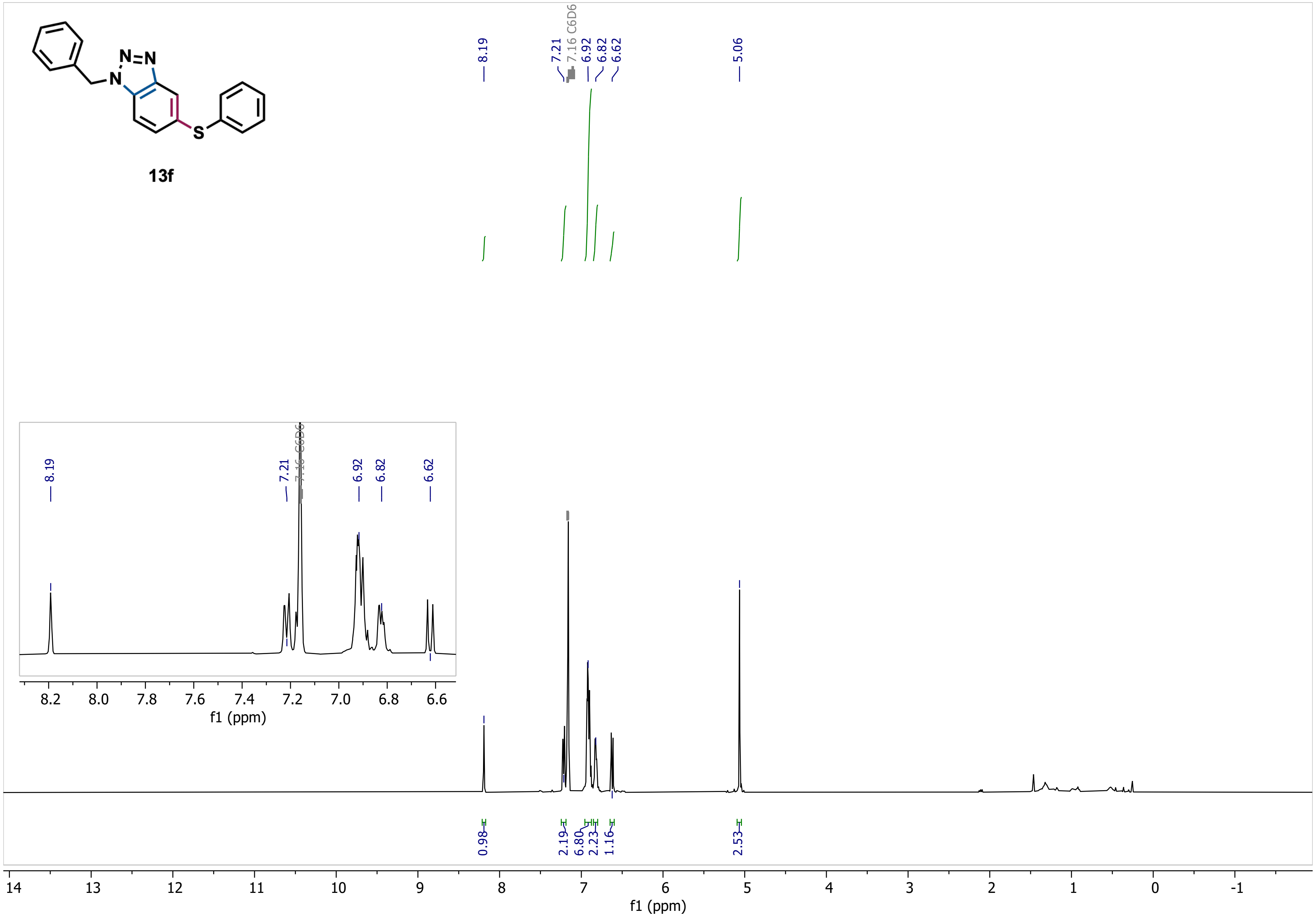

^{1}H NMR (400 MHz, C_6D_6) spectrum of compound **13f**.

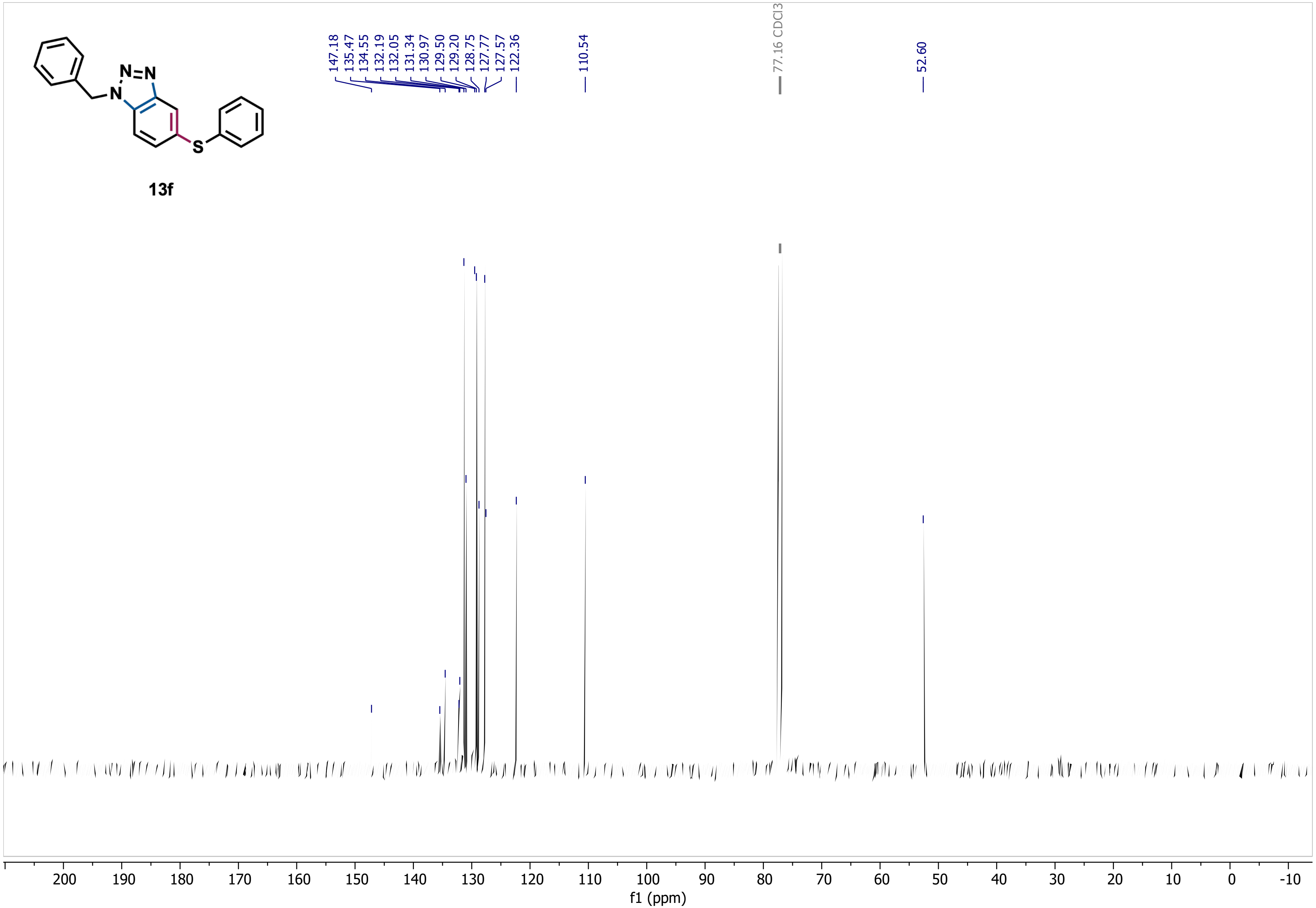

$^{3}C\{^{1}H\}$ NMR (126 MHz, $CDCl_3$) spectrum of compound **13f**.

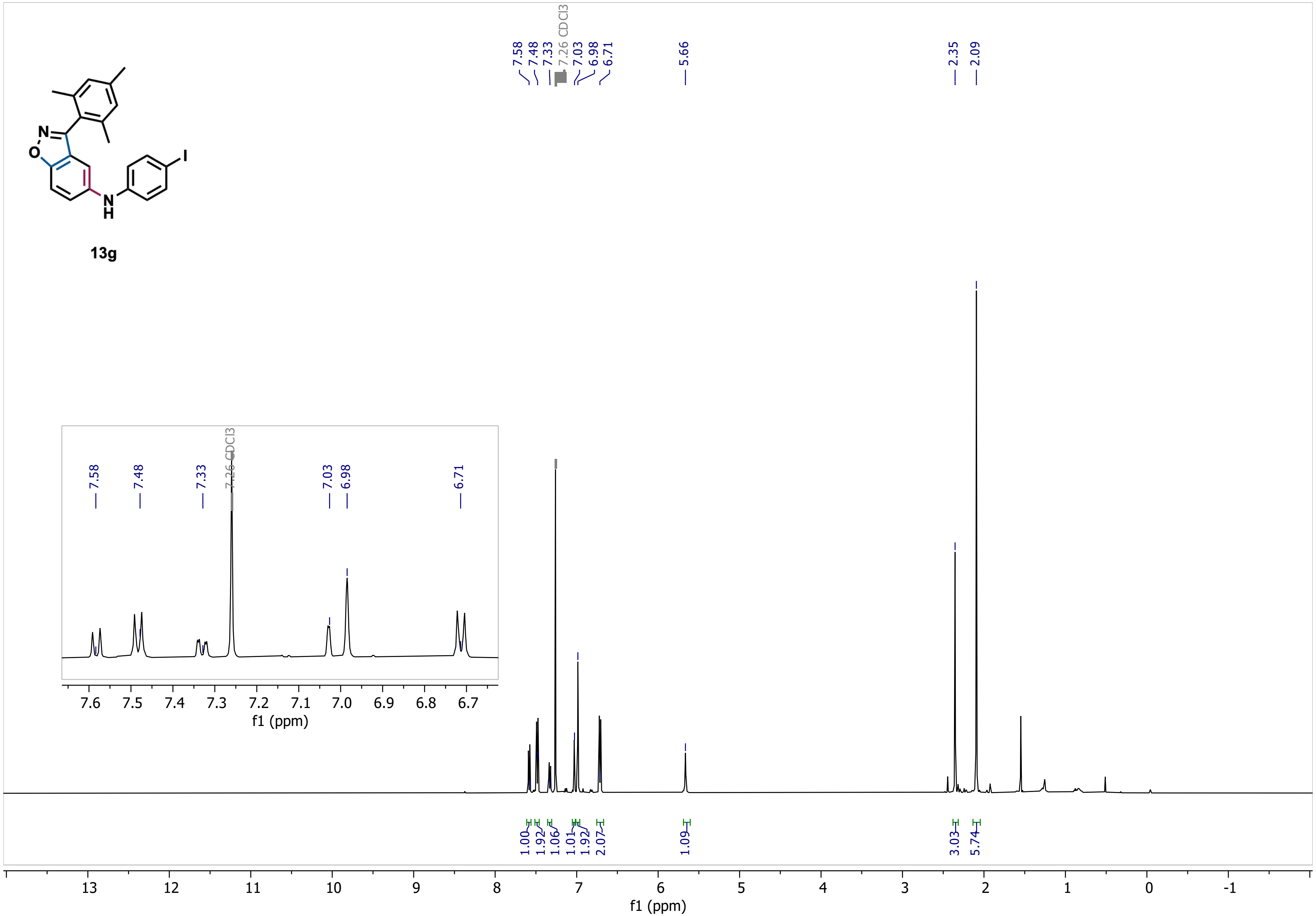

^{1}H NMR (500 MHz, $CDCl_3$) spectrum of compound **13g**.

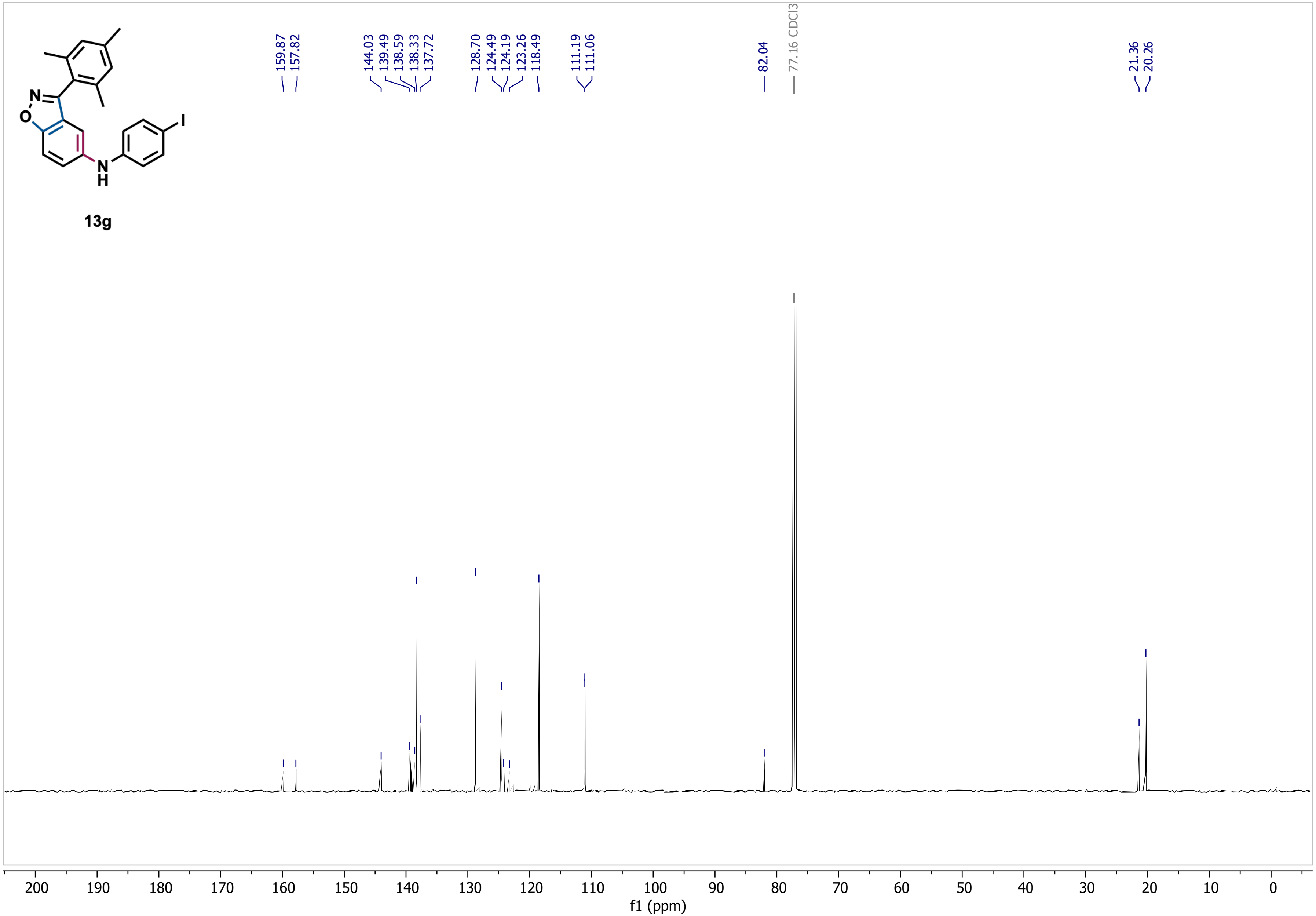

$^{3}C\{^{1}H\}$ NMR (126 MHz, $CDCl_3$) spectrum of compound **13g**.

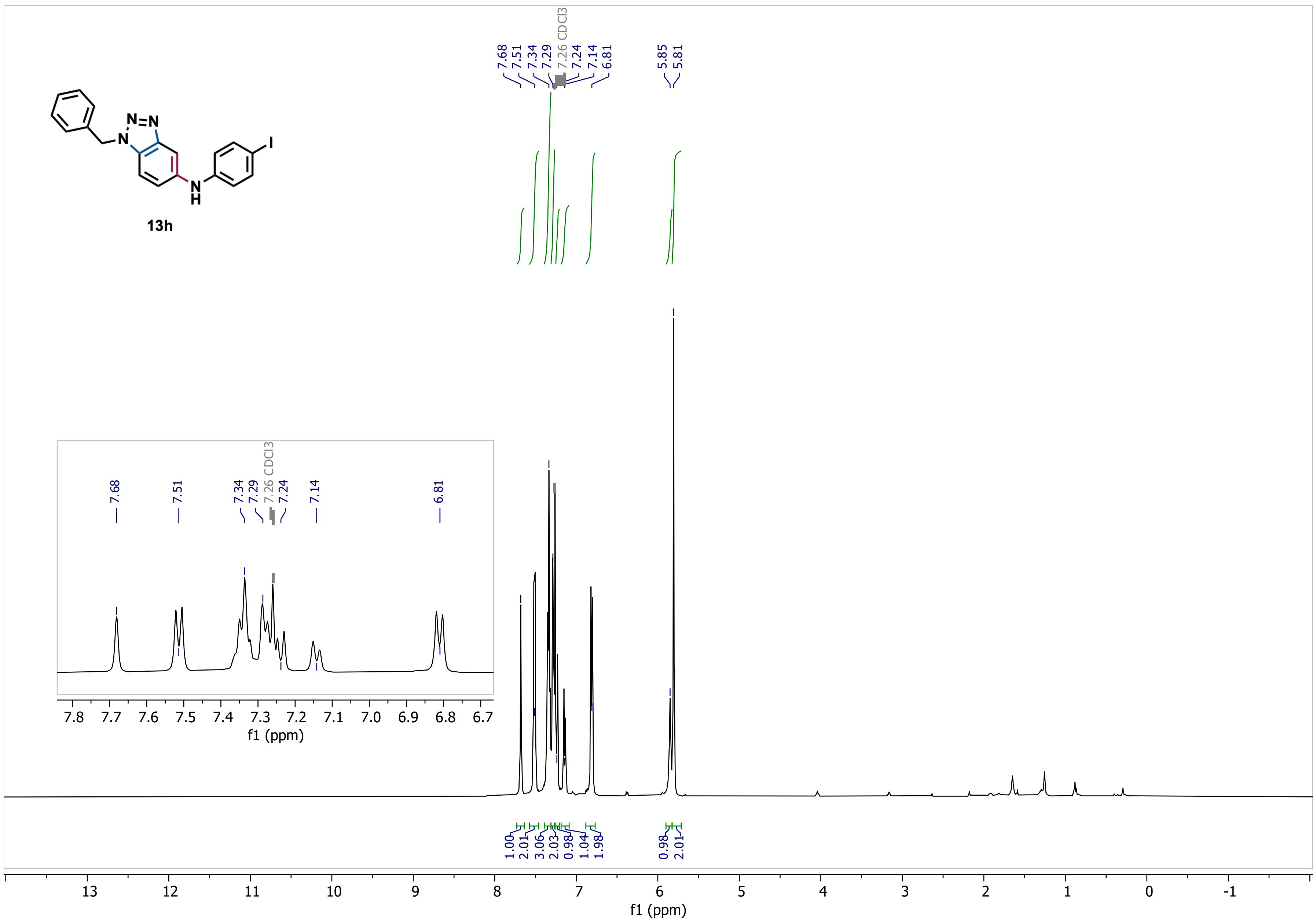

^{1}H NMR (500 MHz, $CDCl_3$) spectrum of compound **13h**.

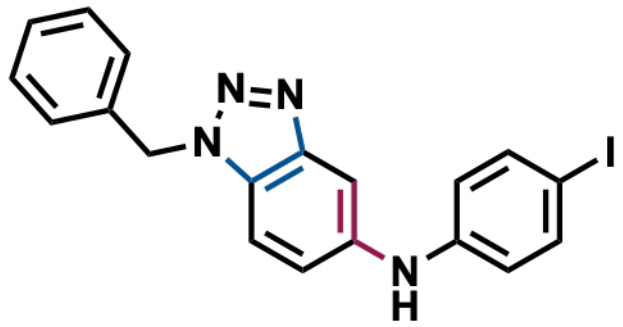

13h

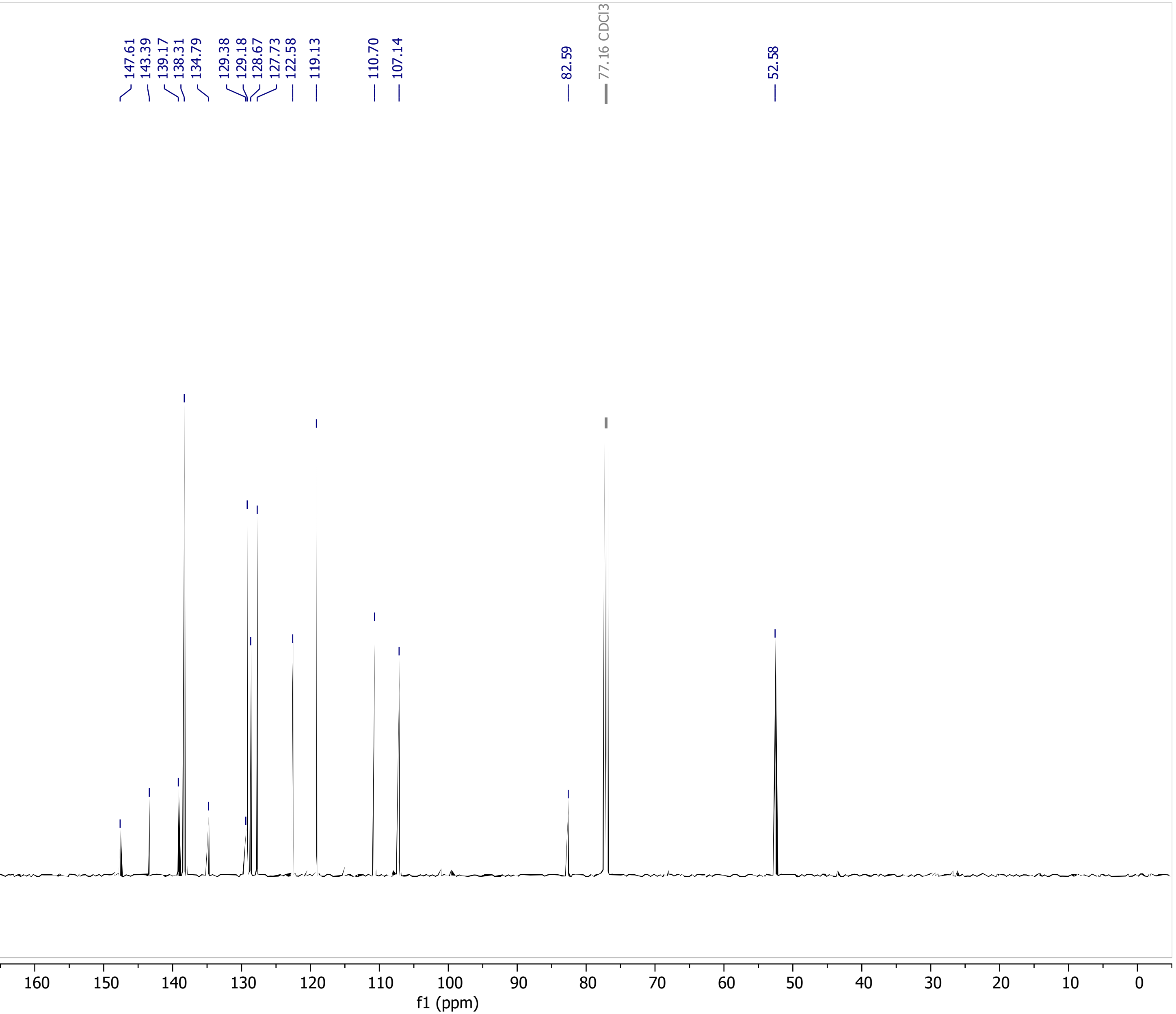

^{3}C{^{1}H} NMR (126 MHz, $CDCl_3$) spectrum of compound **13h**.
